# Design, synthesis, structure, in vitro cytotoxic activity evaluation and docking studies on target enzyme GSK-3β of new indirubin-3ʹ-oxime derivatives

**DOI:** 10.1038/s41598-020-68134-8

**Published:** 2020-07-10

**Authors:** Nguyen Trong Dan, Hoang Duc Quang, Vuong Van Truong, Do Huu Nghi, Nguyen Manh Cuong, To Dao Cuong, Tran Quoc Toan, Long Giang Bach, Nguyen Huu Thuan Anh, Nguyen Thi Mai, Ngo Thi Lan, Luu Van Chinh, Pham Minh Quan

**Affiliations:** 1 Institute of Natural Products Chemistry, Vietnam Academy of Science and Technology, 18 Hoang Quoc Viet, Cau Giay, Hanoi, 11307 Vietnam; 2 Graduate University of Science and Technology, Vietnam Academy of Science and Technology, 18 Hoang Quoc Viet, Cau Giay, Hanoi, 11307 Vietnam; 3 Vietnam-Russia Tropical Center, Nguyen Van Huyen, Nghia Do, Cau Giay, Hanoi, 11307 Vietnam; 4 Faculty of Pharmacy, Phenikaa University, Yen Nghia, Ha Dong, Hanoi, 12116 Vietnam; 5 A&A Green Phoenix Group JSC, Phenikaa Research and Technology Institute (PRATI), No.167 Hoang Ngan, Trung Hoa, Cau Giay, Hanoi, 11313 Vietnam; 6 NTT Hi-Tech Institute, Nguyen Tat Thanh University, Ho Chi Minh City, Vietnam; 7 Center of Excellence for Functional Polymers and NanoEngineering, Nguyen Tat Thanh University, Ho Chi Minh City, Vietnam; 8 Laboratory of Theoretical and Computational Biophysics, Ton Duc Thang University, Ho Chi Minh City, Vietnam; 9 Faculty of Applied Sciences, Ton Duc Thang University, Ho Chi Minh City, Vietnam; 10 Institute of Materials Science, Vietnam Academy of Science and Technology, 18 Hoang Quoc Viet, Cau Giay, Hanoi, 11307 Vietnam

**Keywords:** Analytical biochemistry, Computational biology and bioinformatics

## Abstract

The addition of chalcone and amine components into indirubin-3′-oxime resulted in 15 new derivatives with high yields. Structures of new derivatives were also elucidated through 1D, 2D-NMR and HR-MS(ESI) spectra and X-ray crystallography. All designed compounds were screened for cytotoxic activity against four human cancer cell lines (HepG2, LU-1, SW480 and HL-60) and one human normal kidney cell line (HEK-293). Compound **6f** exhibited the most marked cytotoxicity meanwhile cytotoxicity of compounds **6e**, **6h** and **6l** was more profound toward cancer cell lines than toward normal cell. These new derivatives were further analyzed via molecular docking studies on GSK-3β enzyme. Docking analysis shows that most of the derivatives exhibited potential inhibition activity against GSK-3β with characteristic interacting residues in the binding site. The fast pulling of ligand scheme was then employed to refine the binding affinity and mechanism between ligands and GSK-3β enzyme. The computational results are expected to contribute to predicting enzyme target of the trial inhibitors and their possible interaction, from which the design of new cytotoxic agents could be created in the future.

## Introduction

Cancer is ranked second globally as cause of death. The disease originated due to the inability of cells to control growth, often leading to the formation of cancerous tumors or liquid cancer. (i.e. leukemia and lymphoma cancer). Routines for cancer treatment consist of chemotherapy and radiotherapy where the former utilizes molecule-size drugs aiming at eradication and inhibition of cancer tumors. However, this treatment technique has been shown to suffer from several inherent shortcomings including the development of drug resistance, off-target toxicity and limited targeting capabilities^1,2^.

Glycogen synthase kinase-3 (GSK-3) is defined as a multifunctional serine/threonine protein kinase that regulates the phosphorylation of various cellular targets^3^. The function of GSK-3 is essential for the development of various diseases including cancers, diabetes, Alzheimer’s disease^4,5^. GSK-3 was found to have two isoforms, GSK-3α (51 kDa) and GSK-3β (47 kDa). Both of which were observed in mammalian tissues, and together present an 84% overall similarity. Their kinase catalytic domains share around 98% similarity and are only distinguished by an extra Gly-rich stretch in the N-terminal region of GSK-3α^6^. GSK-3β was recognized to play a significant role in the Wingless (Wnt) signaling pathway, suggesting that the inhibition of GSK-3β could lead to decreased cancer cell proliferation, triggering the p53-dependent apoptosis and stimulate the TRAIL-induced cell death^7^.

Indirubin and indirubin-3′-oxime are well known as potential anticancer agent^8,9^ and thus, a myriad of indirubin derivatives have been devised exhibiting valuable biological activities, high selectivity, and drug-likeness^10^. In addition, recent studies on interactions between indirubin-3′-oxime with the active site of GSK-3β (PDB ID: 1Q41) offered crucial insights into intermolecular interactions and the mechanism of specificity towards this kinase^11^.

In addition, to discover compounds with high biological activity for drug development, virtual screening has been considered as a convenient and economically efficient method. Previous investigations involving GSK-3β inhibitors have produced promising results in pharmacophore design^12,13^, docking^12,14,15^, and elaboration of structure–activity relationships^15,16^. However, the targeting behavior of GSK-3β toward indirubin, indirubin-3′-oxime and its derivatives has not been clearly substantiated. In addition, investigations regarding the relationship between structural differences and the affinity of GSK-3β have been lacking in the literature. The present work focuses on indirubin derivatives due to their promising inhibition activity toward GSK-3β. First, the synthesis of new derivatives by adding chalcone and amine components into indirubin-3′-oxime was attempted with the aim of improving bioactivity and solubility. The structures of obtained derivatives were elucidated through 1D, 2D-NMR and HR-MS(ESI) spectra and X-ray crystallography. Second, due to the essential role of GSK-3β in the development of various cancer types^3, 7^, cytotoxic and selectivity of the derivatives were also evaluated in vitro against four human tumor cell lines (HepG2, LU-1, SW480 and HL-60) and one human normal embryonic kidney cell line (HEK-293). Third, as indirubin and its derivatives are potential GSK-3β inhibitors^14,15^, molecular docking of studied compounds on GSK-3β enzyme were performed to explore the possible interaction within its active site. Fourth, the fast pulling of ligand (FPL) simulation was conducted to refine the binding affinity and mechanism of the ligand (molecule) to GSK-3β. Our results demonstrate the potential application of designed compounds in cancer treatment.

## Results and discussion

### Synthesis studies and structure determination

**Figure 1:**
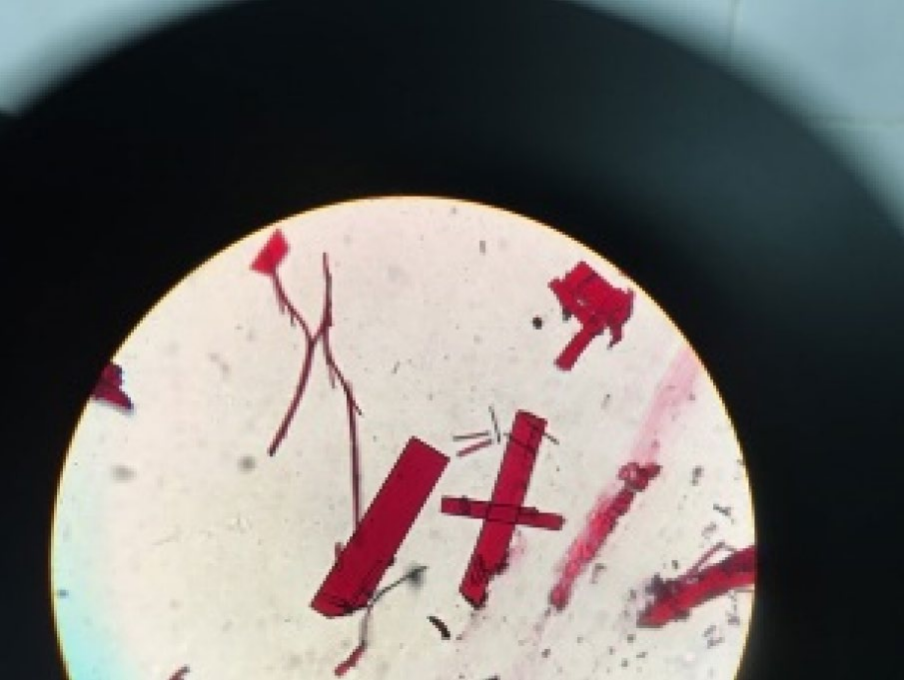
Single crystal **4**. *CCDC 1,917,485 contains the supplementary crystallographic data for this paper. The data can be obtained free of charge from The Cambridge Crystallographic Data Centre via www.ccdc.cam.ac.uk/structures.

**Figure 2:**
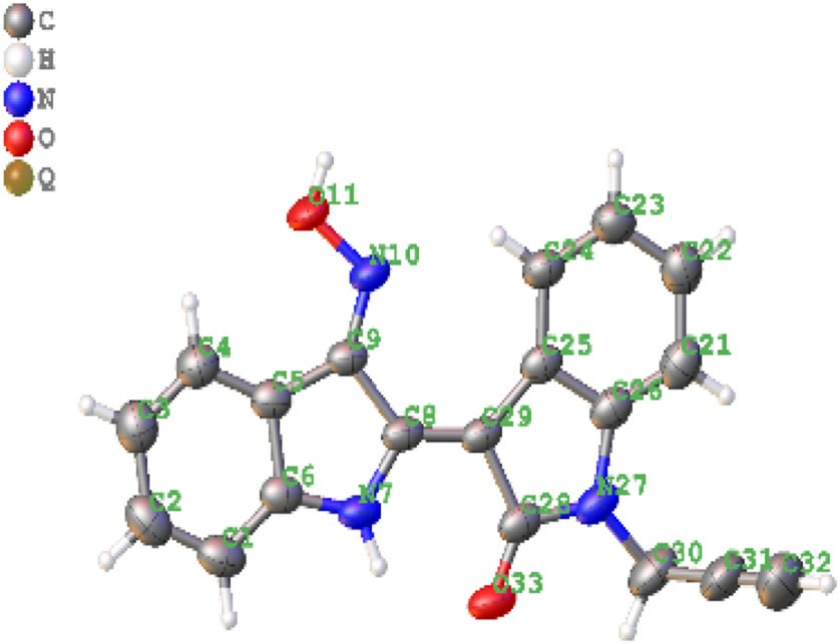
X-ray crystallographic structure of compound **4**. *CCDC 1,917,485 contains the supplementary crystallographic data for this paper. The data can be obtained free of charge from The Cambridge Crystallographic Data Centre via www.ccdc.cam.ac.uk/structures.

**Scheme 1:**
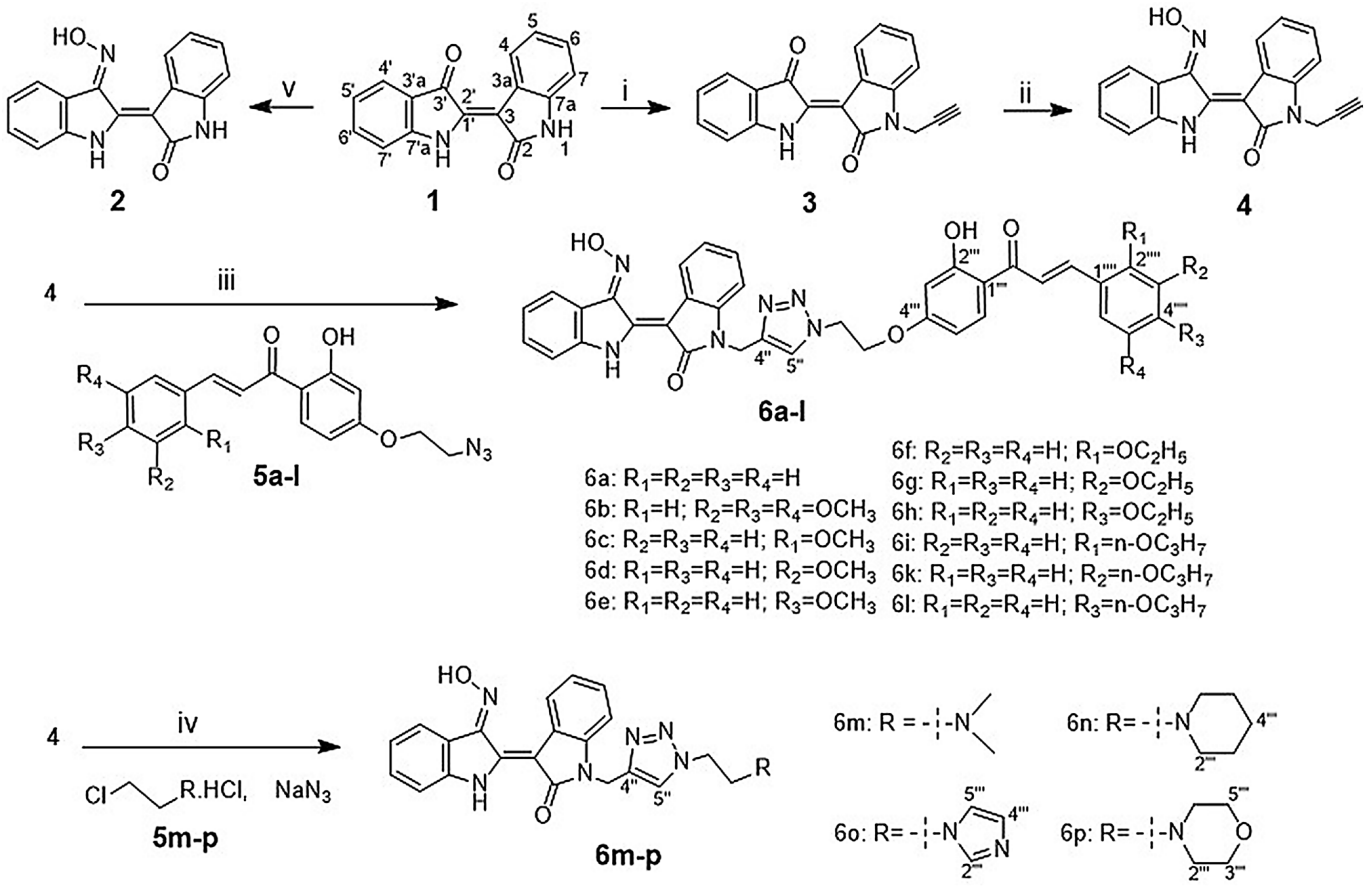
Preparation of new derivatives **6a–p**: Reagents and conditions: (i) propagyl bromide, DMF, K_2_CO_3_, KI, (1-butyl)triethylammonium, 48 h, rt, 59%; (ii) NH_2_OH.HCl, pyridine, 3 h, reflux, 79%; (iii) **5a–l**, CuI, DMSO, 24 h, rt, 57–70%; (iv) **5m–p**, NaN_3_, DMSO, K_2_CO_3_, CuI, 24 h, rt, 56–68%; (v) NH_2_OH·HCl, pyridine, 3 h, reflux, 93%.

The click chemistry reaction between compound **4** and azide chalcones through a 3-step process resulted in 11 new indirubin-3′-oxime derivatives (compounds **6a–l**). At first, compound **3** was obtained (yield 59%) by *N*-alkylation reaction between indirubin **1** and propagyl bromide at room temperature in anhydrous DMF solvent using K_2_CO_3_, KI, 1-(butyl)triethylammonium bromide as base catalyst. Then, compound **4** was formed with high yield (79%) by condensation reaction between **3** and hydroxylamine chlorohydric in reflux pyridine solvent. The structure of compound **4** was determined by NMR, HR-MS(ESI) method in combination with single crystal X-ray diffraction measurement which for the first time confirmed **4** with absolute configuration (*2*′*Z*_*indirubin*_*, 3*′*E*_*oxime*_) (Figs. 1, 2). This result also confirms that indirubin **1** has absolute configuration *2*′*Z*^17^. Finally, the azide-alkyne cyclisation reaction between compound **4** and various azide chalcones (**5a–l**) in DMSO at room temperature with CuI as catalyst afforded 11 new triazoles (**6a–l**) in 57–70% isolated yield (Scheme 1). Four new indirubin-3′-oxime derivatives **6m–p** were synthesized with yield 56 ÷ 68% by reaction between compound **4** and solution **A** using CuI as catalyst at room temperature (Scheme 1).

The structures of obtained compounds were determined by 1D, 2D-NMR and HR-MS(ESI) spectroscopic methods in which compounds **6a** was chosen as the representative molecule to determine the exact structure with the combination of COSY, HSQC and HMBC spectral evidence. In the ^1^H-NMR spectrum of **6a**, proton signals of the group OH oxime, OH chalcone and H-1′ indirubin appeared as singlet at 13.57, 13.35 and 11.78 ppm, respectively. A doublet signal at 8.7 ppm (*J* = 8 Hz) could be assigned to proton H-4. One triplet signal at 8.24 ppm is the interlacing proton signal of H-6‴ and H-4′*.* Singlet signal at 8.12 ppm corresponds to proton H-5″, characteristic of 1,2,3-triazole substituted at 1 and 4 position. Two doublet signals with the ‟roof effect” that characterize for the chalcone *E* configuration at 8.00 ppm (*J* = 15.5 Hz) and 7.83 ppm (*J* = 15.5 Hz) were assigned to two protons H-α and H-β, respectively. The remaining 3 protons of the H-4‴′, H-3‴′ and H-5‴′ aldehyde in chalcone appeared as multilet at 7.48 ppm. Another multilet at 7.42 ppm, next to H-3‴′, H-5‴′ was assigned to two protons H-6′, H-7′. Four remaining protons of indirubin component appearing as multilet at 7,17 ppm and 7,00 ppm were assigned to H-6, H-7 and H-5′, H-5, respectively. The proton signal of H-3‴, H-5‴ of ketone component in chalcone was confirmed at 6.50 ppm. Singlet signal at 5.15 ppm corresponded to 2 protons of CH_2_–C-4″. Two triplet at 4.74 ppm (*J* = 4.75 Hz) and 4.49 ppm (*J* = 4.75 Hz) were respectively assigned to proton signals of –CH_2_–CH_2_–O– and –CH_2_–CH_2_–O–. Based on HSQC spectra, carbon atoms are also precisely defined as follow: 122.8 ppm (C-4); 132.8 ppm (C-6‴); 127.9 ppm (C-4′); 123.9 ppm (C-5″); 121.2 ppm (C-α); 129.1 ppm (C-2‴′, C-6‴′); 144.3 ppm (C-β); 130.9 ppm (C-4‴′); 128.9 ppm (C-3‴′, C-5‴′); 132.1 ppm (C-6′); 111.6 ppm (C-7′); 125.7 ppm (C-6); 108.3 ppm (C-7); 121.7 ppm (C-5′); 121.0 ppm (C-5); 101.7 ppm (C-3‴); 107.6 ppm (C-5‴); 34.5 ppm (CH_2_–C-4″); 48.7 ppm (–CH_2_–CH_2_–O–) and 66.5 ppm (–CH_2_–CH_2_–O–). In addition, there are proton signal interaction between CH_2_–C-4″ and C-2 (168.6 ppm); C-4″ (142.8 ppm); C-7a (138.3 ppm) and C-5″ (123.9 ppm). The correlation signal between proton of –CH_2_–CH_2_–O– and C-5″ (123.9 ppm) in HMBC spectrum combined with HR-MS(ESI) method confirmed the exact structure of compound **6a**. The structure of the remaining derivatives are also completely consistent with NMR and HR-MS(ESI) data. Combining NMR, HR-MS(ESI) spectra of compounds **6a–p** with the absolute configuration of compound **4**, it is determined that compounds **6a–l** have absolute configuration (*2*′*Z*_*indirubin*_*, 3*′*E*_*oxime*_*, E*_*chalcone*_). Compounds **6m–p** have the absolute configuration (*2*′*Z*_*indirubin*_*, 3*′*E*_*oxime*_) similar to the absolute configuration of compound **4**. Detail spectroscopic datas of synthesized compounds are provided in Supplementary information S3 and S6.

### Cell viability assay

To gain better insight into the potential anticancer activities of the synthesized compounds, we performed in vitro cytotoxicity assay on four human cancer cell lines and one human normal kidney cell line. In the present study, starting material compounds including indirubin (**1**), compound **3**, compound **4** and indirubin-3′-oxime (**2**) along with other derivatives (**6a–p**) were tested for cytotoxicity against HepG2, LU-1, SW480, HL-60 and HEK-293 cell lines using Ellipticine and indirubin-3′-oxime as standard (Table 1). In the past, indole derivatives have been identified as GSK-3 inhibitors^18^. Indirubins are likewise indole derivatives and their biological activities have given rise to the widespread use, such as in remediation of leukemias, in traditional Chinese medicine^19^. According to obtained results, indirubin (**1**) was identified to have no activity due to their IC_50_ value being higher than 20 μM. This was also observed in some of its derivatives including compounds **3** and **4** which exhibited weak signals of cytotoxicity against four cancer cell lines.

**Table 1:** In vitro cytotoxic activity of starting material compounds and indirubin-3′-oxime derivatives **6a–p**. Indirubin-3′-oxime (compound **2**) and Ellipticine was used as positive control.

No.	Compounds	IC_50_ (μM)				
		HepG2	LU-1	SW480	HL-60	HEK-293
1	**1**	> 20	> 20	> 20	> 20	> 20
2	**3**	> 20	> 20	> 20	> 20	> 20
3	**4**	> 20	19.55 ± 1.23	18.59 ± 0.91	15.27 ± 0.35	16.22 ± 0.33
4	**2**	16.00 ± 0.62	16.36 ± 1.05	15.65 ± 0.74	16.50 ± 0.94	14.16 ± 0.71
5	**6a**	3.56 ± 0.75	2.26 ± 0.32	3.36 ± 0.64	2.39 ± 0.46	3.21 ± 0.43
6	**6b**	> 20	> 20	> 20	> 20	> 20
7	**6c**	3.38 ± 0.68	2.99 ± 0.55	3.8 ± 0.52	1.43 ± 0.22	1.19 ± 0.12
8	**6d**	3.76 ± 0.75	2.85 ± 0.74	4.46 ± 1.18	3.40 ± 0.76	2.01 ± 0.64
9	**6e**	4.62 ± 1.07	3.28 ± 0.48	4.66 ± 0.92	3.27 ± 0.37	6.98 ± 0.25
10	**6f**	2.01 ± 0.43	1.30 ± 0.14	2.54 ± 0.25	0.98 ± 0.12	1.03 ± 0.11
11	**6g**	5.68 ± 0.71	3.96 ± 0.62	4.10 ± 0.57	4.82 ± 0.46	1.80 ± 0.21
12	**6h**	14.23 ± 1.79	8.74 ± 0.55	14.13 ± 1.18	6.50 ± 0.98	9.74 ± 0.53
13	**6i**	2.28 ± 0.12	2.07 ± 0.26	3.43 ± 0.84	1.28 ± 0.34	0.85 ± 0.05
14	**6k**	2.95 ± 0.19	2.50 ± 0.34	2.91 ± 0.13	1.29 ± 0.14	1.92 ± 0.17
15	**6l**	3.28 ± 0.25	2.07 ± 0.31	3.03 ± 0.11	1.28 ± 0.16	7.12 ± 0.18
16	**6m**	15.31 ± 0.17	8.74 ± 1.06	14.36 ± 0.72	15.96 ± 0.38	11.93 ± 1.75
17	**6n**	11.70 ± 0.23	10.08 ± 0.44	12.54 ± 0.53	10.44 ± 0.84	9.52 ± 0.07
18	**6o**	11.68 ± 0.26	8.80 ± 0.38	11.16 ± 0.76	9.39 ± 0.45	5.89 ± 0.75
19	**6p**	10.61 ± 0.79	8.61 ± 0.57	11.71 ± 0.48	11.25 ± 1.04	7.72 ± 0.42
20	Ellipticine	1.93 ± 0.54	2.50 ± 0.82	1.76 ± 0.63	2.19 ± 1.12	0.32 ± 0.03

The chalcone component (**6a–l**) derivatives offer clear advantages in anti-proliferation activities in comparison to its parent compounds. Compound **6f** exhibited the strongest activity with IC_50_ values of 2.01 ± 0.43, 1.30 ± 0.14, 2.54 ± 0.25, 0.98 ± 0.12 and 1.03 ± 0.11 μM on HepG2, LU-1, SW480, HL-60 and HEK-293, respectively, which are considerably more toxic than indirubin-3′-oxime. In addition, structural data from compound **4** and series **6a–l** in combination with cytotoxic and anti-proliferation activities prompt the speculation that the presence of oxime group in the structure of studied compounds could play an important role in inhibition of cancer cell activity. High IC_50_ values of chalcone derivatives of compound **4** also suggest the role of OC_3_H_7_ group in triggering cytotoxic activities through conformational changes. The exception was observed with compound **6b** (IC_50_ > 20 μM against all tested cell lines), which is in line with poor interaction with GSK-3β, calculated from the following molecular docking studies. This result suggests the simultaneous intervention of methoxy groups at R_2_, R_3_, R_4_ position resulted in the decreased cytotoxicity. It is noteworthy that different substitution groups at R_3_ position resulted in toxicity selectivity between cancer and normal cell line of compounds **6e, 6h** and **6l**. The selectivity is evidenced by the slightly lower IC_50_ values of compounds **6e, 6h** and **6l** on HEK-293, at 6.98 ± 0.25, 9.74 ± 0.53 and 7.12 ± 0.18 μM, respectively, in comparison to IC_50_ values obtained with the other four cancer cell lines.

IC_50_ values of the series **6m–p** were slightly lower than those of **6a–l** and were approximately equivalent to each other. However, the addition of amine components into indirubin-3′-oxime, generating the series **6m–p**, seemed to result in better cytotoxicity and improved solubility properties and better cytotoxicity.

### Docking studies

Autodock 4.2.6 was utilized to predict the interaction of all molecules with GSK-3β enzyme model. The X-ray crystallographic structure complex with co-crystalized ligand (indirubin-3′-oxime) was collected from Protein Data Bank (RCSB). Based on literature studies, two known inhibitors of GSK-3β (CHIR-98014 and BIO-acetoxime) were chosen as standard ligands for docking validation^20,21^. Obtained dock score for these two inhibitors were − 11.82 kcal/mol and − 10.69 kcal/mol, respectively, thus, the threshold value of the docking energy also determined as − 10.69 kcal/mol and any molecules whose docking energies are close to this threshold would be viewed as potential inhibitors of GSK-3β in the virtual screening stage. In general, results from the docking procedures showed that the compound **6f** has the highest docking score, at − 14.09 kcal/mol, far exceeding than that of the standard ligand (Table 2). It is noted that the initial compounds including **1**, **3** and **4** had lower absolute dock score than indirubin-3′-oxime (**2**). However, higher absolute values of docking scores of chalcone-added components (**6a–l**) and amine-added components (**6m–p**) through triazole bridge into the compound **4** structure suggested better binding affinities toward GSK-3β. The dock score values were further converted to the prediction inhibition constants (K_i_,_pred_). Through comparing the K_i_ values in Table 2, high correlation between order of cytotoxic activity (IC_50_), dock score and K_i_ of tested compounds was observed. For example, compound **6f** was recorded to have the most negative value (46.6E−12M) and its IC_50_ and dock score was the most potential in comparision to those of others.

**Table 2:** Set of designed compounds with respective docking score (kcal/mol).

Designed compounds	Dock score (kcal/mol)	K_i,pred_ (M)	Designed compounds	Dock score (kcal/mol)	K_i,pred_ (M)
**1**	− 9.08	222.71E−09	**6h**	− 11.53	3.53E−09
**2**	− 9.51	107.82E−09	**6i**	− 12.85	380.41E−12
**3**	− 8.49	600.31E−09	**6k**	− 12.73	467.69E−12
**4**	− 9.30	151.80E−09	**6l**	− 13.78	79.11E−12
**6a**	− 13.64	99.73E−12	**6m**	− 9.88	57.51E−09
**6b**	− 9.34	1.39E−06	**6n**	− 11.55	3.43E−09
**6c**	− 13.17	222.30E−12	**6o**	− 11.19	6.27E−09
**6d**	− 11.82	2.18E−09	**6p**	− 11.25	5.71E−09
**6e**	− 13.18	219.20E−12	CHIR-98014	− 11.82	2.18E−09
**6f**	− 14.09	46.6E−12	Bio-acetoxime	− 10.69	14.06E−09
**6g**	− 13.72	87.29E−12			

**Figure 3:**
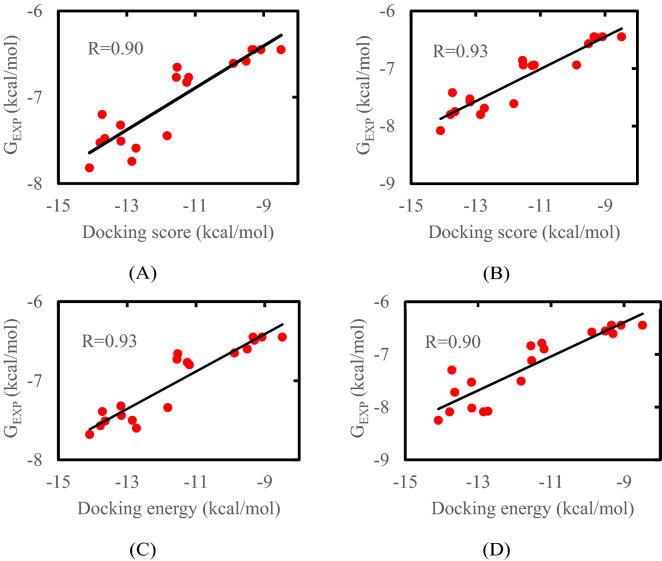
The correlation between dock score and experimental binding free energies on four cell line models. (**A**) HepG2; (**B**) LU-1; (**C**) SW480; (**D**) HL-60.

The relationship between dock score and experimental binding free energies on four cell lines was shown in Fig. 3. The experimental binding free energy was calculated from formula ΔG_exp_ = RT lnK_i_ where gas constant R = 1.987 × 10^−3^ kcal K^−1^ mol^−1^ and absolute temperature T = 300 K. Here, in this equation the IC_50_ was assumed as equal to the inhibition constant K_i_ and measured in moles. The obtained correlation coefficient implies the high accuracy of the computations for all four models HepG2, LU-1, SW480 and HL-60 with R = 0.90; 0.93; 0.93 and 0.90, respectively.

**Figure 4:**
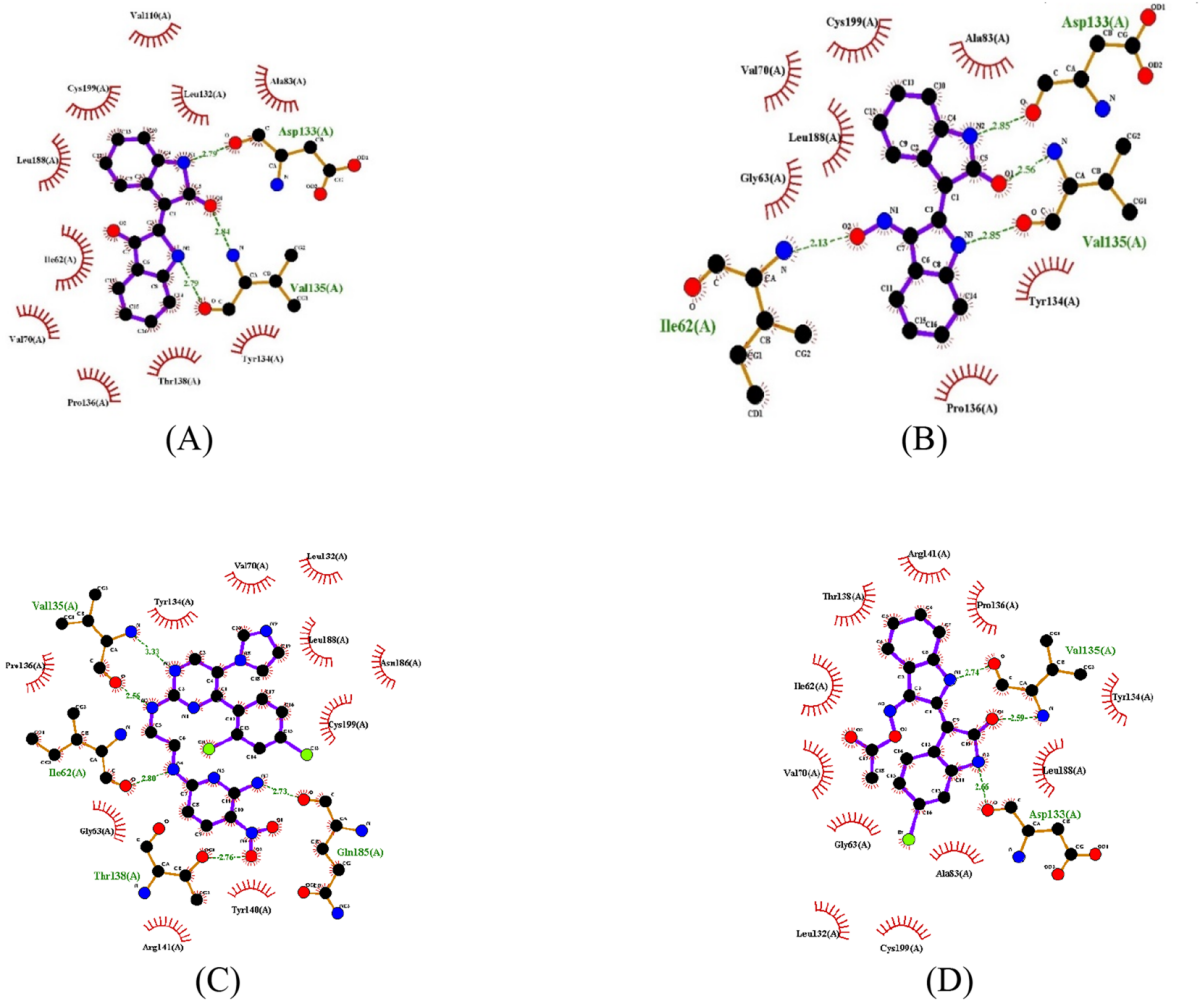
Hydrogen bonding patterns of compounds with GSK-3β protein (PDB ID: 1Q41). (**A**) Compound **1**; (**B**) Compound **2**; (**C**) CHIR-98014; (**D**) Bio-acetoxime.

The role of polar residue groups such as Asp133, Tyr134 and Val135 situating on the binding cavity in the ligand-ATP recognition and affinity has been confirmed and emphasized, due to the interaction of Asp200 with the phosphate hydroxyl group of ATP^22^. In addition, previous crystallographic evidence on GSK-3β interaction with various inhibitors has highlighted the role of Asp133 and Val135 in enhancing affinity to GSK-3β. Other residues such as Ile62, Gln185 and Cys199 were also shown to contribute to the interaction of the inhibitor to the backbone (Table 3). Docking validation analysis reveals that CHIR-98014 creates five H-bonds toward GSK-3β through four residues Ile62, Thr138, Val135 and Gln185. Bio-acetoxime, another known inhibitor, formed 3 hydrogen bonds with the enzyme target including amino acids Asp133 and Val135 (Fig. 4). In this study, the following compounds **6a**, **6c**, **6f**, **6i** with substituents changes in R_1_ position and **6b** were taken as the representatives to analyze the mechanism of actions. The potential interactions between the ligands and GSK-3β were further elaborated by evaluating residues within 5 Å of the ligands.

**Table 3:** Potential compounds displaying various H-bond interacting residues.

Designed compounds	No. of H-bonds	Interacting residues	Designed compounds	No. of H-bonds	Interacting residues
**1**	3	Asp133, Val135	**6c**	4	Ile62, Val135, Asn186, Cys199
**3**	1	Val135	**6f**	3	Val135, Thr138
**4**	1	Val135	**6i**	3	Asn64, Tyr134, Gln185
**2**	4	Ile62, Asp133, Val135	**6n**	3	Ile62, Val135, Cys199
**6a**	3	Ile62, Val135, Gln185	CHIR-98014	5	Ile62, Thr138, Val135, Gln185
**6b**	1	Ile62	Bio-acetoxime	3	Asp133, Val135

The carbonyl and two amine groups of compound **1** formed three hydrogen bonds toward carbonyl oxygen of Asp133 and Val135 in the hinge region however its docking score (− 9.08 kcal/mol) is lower than Indirubin-3′-oxime (**2**) (− 9.51 kcal/mol) thus excluding this compound as an appropriate inhibitor (Table 3 and Fig. 4, S3).

Compound **3** had slightly lower dock score (− 8.49 kcal/mol) and was recognized to form only one hydrogen bond with Val135 on the side that is opposite to the Val135-binding side in compound **1**. This is possibly due to a conformational change exhibited by alkyl group attached to amidine position (Figures S1, S3).

In compound **4**, the carbonyl group has been replaced with an oxime group, this modification could be the crux to ameliorate docking energy of compound (− 9.30 kcal/mol). Ile62, Asp133, Tyr134, Pro136, Gln185 and Cys199 were the key residues involved in hydrophobic interactions (Figure S2). The key hydrogen bond with Val135 appears to be unique to this oxime group which further stabilizes the interaction of compound and provides an interesting model to explain the biological activities observed for the designed compounds (Table 3 and Figures S1, S3). Therefore, this group should be kept in lead optimization process.

Removal of alkynyl group in compound **4** resulted in indirubin-3′-oxime (**2**). The absence of this group might have made the space, which possibly caused the two amine groups and carbonyl group to interact with residues Asp133, Val135 through 3 hydrogen bonds with distance of 2.85, 2.56 and 2.85 Å, respectively (Table 3 and Figures S1, S3). The interaction was also indicated by a significant docking score (− 9.51 kcal/mol). The oxime group in this case do not interact with Val135 but contribute one hydrogen bond with Ile62 to stabilize the ligand. In addition, an array of hydrophobic interactions was observed for Gly63, Val70, Ala83, Tyr134, Pro136, Leu188 and Cys199 (Figure S2).

Compounds **6a**, **6c**, **6f** and **6i** are chalcone-added components and are differentiated by the substituents at R_1_ position (H, OCH_3_, OC_2_H_5_, OC_3_H_7_). Binding energy values to GSK-3β of those ligands were reported to be − 13.64, − 13.17, − 14.09 and − 12.85 kcal/mol, respectively (Table 2). It is noted that the oxime group in compounds **6c** and **6f** were observed to initiate hydrogen bond with Val135. Compound **6a** formed H-bonding with two residues Ile62 and Val135 similar to those formed by indirubin-3′-oxime. The third H-bond interaction between hydroxyl group of **6a** and the carboxyl oxygen of Gln185 could be the key for its high bioactivity. Figure S1 showed the binding of **6c**, which was characterized by H-bonding with Ile62, Asn186 and Cys199. Hydrophobic bondings were also found to stabilize interaction of **6c** with GSK-3β through residues such as Val70, Lys85, Arg141, Gln185 and Leu188 (Figure S2). The substituent OC_2_H_5_ located at the R_1_ position of compound **6f** was found to interact with Thr138 active site region, which suggests the importance of this residue in the formation of ligand binding affinities. Besides, the hydrophobic pockets formed with **6f** including Ile62, Asn64, Val70, Ala83, Lys85, Asp133, Arg141, Cys199 also strengthen the interaction between ligand and the protein target (Figure S2). Being different from compounds **6c** and **6f**, compound **6i** showed the formation of the hydrogen bond between its oxime group and carbonyl oxygen of Gln185. Presumably, this phenomenon is due to conformational change caused by the bulky substituent OC_3_H_7_. The conformational change also causes the carbonyl and hydroxyl group of this ligand anchored to Asn64 and Tyr134 via two hydrogen bonds (Figures S1, S3).

The bulky substituents situated at R_2_, R_3_ and R_4_ position in compound **6b** could be the reason leading to drastically decreased docking energy (− 9.34 kcal/mol), and the formation of only one hydrogen bond with Ile62 at the oxime group (Table 3 and Figure S1). This result suggests that without simultaneous interaction with Ile62 and Val135, ligand could not trigger inhibition activity against GSK-3β.

The azide-alkyne cyclization reaction (click chemistry reaction) between compound **4** and solution **A** results in compound **6n**. Analysis of the dock pose shows that **6n** exhibited two H-bonds with common residues at Ile62 and Val135, which is similar with that shown by indirubin-3′-oxime (**2**) and **6a,** and another exclusive hydrogen bond with Cys199. Compound **6n** also formed eleven hydrophobic contacts with Asn64, Phe67, Val70, Tyr134, Pro136, Thr138, Lys183, Gln185, Asn186, Leu188, Asp200, thus, resulting in a high fit score (− 11.55 kcal/mol) (Figures S1, S2).

Calculated pharmacokinetic parameters including miLogP (octanol–water partition coefficient), TPSA (total molecular polar surface area), MW (molecular weight) and toxicity prediction of indirubin-3′-oxime derivatives are shown in Table 4.

**Table 4:** Pharmacokinetic parameters and toxicity prediction of research compounds.

Designed compounds	miLogP	TPSA (Å^2^)	MW (g/mol)	LD_50_ (mg/kg)	Toxicity prediction*
**1**	2.9	65.72	262.27	1,000	4
**2**	3.29	81.25	277.28	1,000	4
**3**	3.13	54.87	300.32	1,000	4
**4**	3.52	70.39	315.33	1,000	4
**6a**	6.58	147.64	624.66	1,000	4
**6b**	6.21	157.34	714.74	1,000	4
**6c**	6.41	156.87	654.68	1,000	4
**6d**	6.62	156.87	654.68	1,000	4
**6e**	6.64	156.87	654.68	1,000	4
**6f**	6.79	156.87	668.71	1,000	4
**6g**	6.99	156.87	668.71	1,000	4
**6h**	7.01	156.87	668.71	1,000	4
**6i**	7.29	156.87	682.74	1,000	4
**6k**	7.49	156.87	682.74	1,000	4
**6l**	7.52	156.87	682.74	1,000	4
**6m**	3.14	104.34	429.48	1,000	4
**6n**	4.05	104.34	469.55	1,000	4
**6o**	2.79	118.93	452.48	1,000	4
**6p**	2.99	113.58	471.52	1,000	4
Ellipticine	4.28	28.68	246.31	178	3

*Toxicity prediction class: 1 → 6 (High toxicity to non-toxic).

In general, although the molecular weights of designed compounds were high, their TPSA were significantly higher than that of Ellipticine, indicating that designed molecules might pass through membrane more easily, making them particularly suitable for oral use. In addition, the calculated miLogP of derivatives **6m–p** were lower than those of **6a–l**, indicating better solubility of these compounds than those of the others. Interestingly, predicted toxicities of all the designed derivatives were lower than that of the commercial drug Ellipticine. The LD_50_ value of Ellipticine was 178 mg/kg, classifying it as a toxic compound. Meanwhile, predicted LD_50_ values of all designed compounds was 1,000 mg/kg, indicating that they are ranked as moderately toxic compounds. All these results suggest the potential of indirubin-3′-oxime derivatives in the treatment of cancer.

### FPL simulation

Computer-aided drug design is often used to explore the probable inhibitor, resulting in a reduction of cost and time for therapeutic growth^23^. During the issue, the ligand-binding affinity is normally required to be calculated^24^ since a more efficient ligand often adopts a stronger binding affinity^25^. Numerous approaches were developed in order to resolve the problem^26–29^. Among these techniques, the fast pulling of ligand (FPL) is a very efficient method with low-required CPU time consumption^30^. In particular, a ligand is forced to travel from bound to unbound states via a harmonic-external force. The physical details during unbinding process reveal the binding affinity and mechanism of a ligand to GSK-3β enzyme.

The FPL technique was applied to 10 complexes as shown in Table S3 of the Supplementary (SI file). The relative binding affinity of a ligand to GSK-3β enzyme was estimated using 8 independent FPL calculations. The pulling force was recorded every 0.1 ps and other metrics were monitored every 10 ps. All of the computed values were averaged over 8 independent trajectories. The recorded pulling force and work were shown in Figures S4 and S6. The displacement of a ligand during FPL simulations was mentioned in Figure S5. The ligand-binding affinity is able to estimate via the difference of interaction energy between a ligand and GSK-3β enzyme over the FPL simulations referring to the previous work^30^.

**Figure 5:**
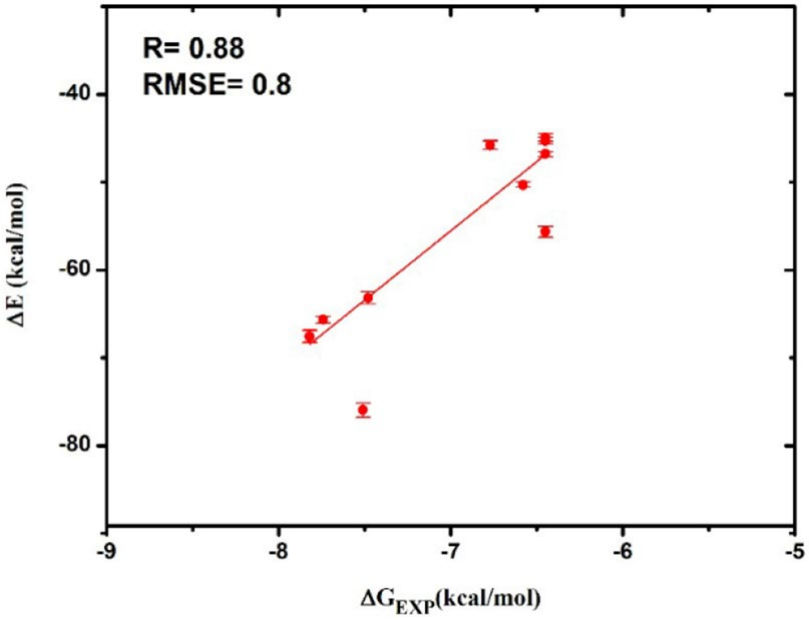
The correlation between the difference of interaction energy between a ligand and GSK-3β enzyme and the experimental value. RMSE was estimated with a linear regression.

The interaction energy term was computed via formula $\Delta E = \Delta E_{cou} + \Delta E_{vdW} = (E_{cou}^{full} - E_{cou}^{0} ) + (E_{vdW}^{full} - E_{vdW}^{0} )$, where $E_{cou}^{full}$ and $E_{cou}^{0}$ are electrostatic interaction energy between a ligand and GSK-3β enzyme at *bound* and *unbound* states, respectively; $E_{vdW}^{full}$ and $E_{vdW}^{0}$ are vdW interaction energy between a ligand and GSK-3β enzyme at *bound* and *unbound* states, respectively. The interaction energy curve was shown in Figure S7. The obtained results were described in Table S3. The total interaction energy difference adopts a high correlation with respected experiments ($R = 0.88$) shown in Fig. 5. The obtained correlation coefficient implies the high accuracy of the computations. Moreover, the precision of the calculated results is also high because the computed error is small, which was estimated using root-mean-square error (RMSE) with linear regression $RMSE = 0.8$ kcal/mol. In addition, the binding mechanism of a ligand to GSK-3β enzyme was also characterized. The vdW interaction energy controls the binding process of a ligand to GSK-3β enzyme. In particular, the vdW term accounts for ca. 79% of total interaction energy that is significantly stronger than the electrostatic term, which accounts for ca. 21% only. When designing new inhibitor for GSK-3β enzyme, the issue should be considered carefully.

## Conclusion

In conclusion, we have described an efficient pathway for the synthesis of novel indirubin-3′-oxime derivatives with advantages of simple operation conditions and high yields. Structures of compounds were elucidated through spectroscopic methods and crystal structure of compound **4** has been clearly determined using X-ray crystallography method. Testing of synthesized derivatives for in vitro cytotoxic activity towards four cancer cell lines (HepG2, LU-1, SW480, HL-60) and one human normal kidney cell line (HEK-293) demonstrated considerable cytotoxic and antiproliferative potential, especially for compound **6f**. Compounds **6e**, **6h** and **6l** with substitution groups at R_3_ position exhibited toxicity selectivity between cancer and normal cell lines. Molecular docking studies have shed light on the interaction of these compounds towards GSK-3β. The binding affinity of tested compounds are in high correlation with experimental binding free energy, besides, the probable binding poses proposed by the docking simulation could offer a plausible explanation for the impact of different substituent components to the bioactivity of derivatives. The FPL approach evaluates the ligand-binding affinity of GSK-3β enzyme with high accuracy (R = 0.88) and precision (RMSE = 0.8 kcal/mol). The vdW term dominates over the electrostatic ones during the binding process of a ligand to GSK-3β. When designing a new inhibitor for GSK-3β enzyme, these issues should be carefully considered. In general, our results suggest that the proposed routine is an important orientation for the design and synthesis of new anticancer agents from indirubin-3′-oxime.

## Materials and methods

### General synthesis procedures

All chemicals were purchased from Sigma-Aldrich (USA), VWR (EU) except for **2**, **5a–l** were prepared according to previous procedures^31,32^. The isolation of Indirubin (1) was carried out using *Strobilanthes cusia* plant materials growing in Vietnam in (2′Z) configuration according to the procedures of Cuong et al.^33^. Procedures of Perrin et al. was employed to prepare dry solvents^34^. Buchi 530 (Switzerland) melting point apparatus was utilized to measure melting points in open capillary tubes and was uncorrected. NMR spectra was recorded at 500 MHz for 1H and 125 MHz for 13C using Bruker Advance 500 MHz. Chemical shifts were reported in parts per million (ppm) with internal standard tetramethylsilane (TMS). Mass spectra was recorded using Agilent 6,530 Accurate-Mass Q-TOF LC/MS. The intensities for the X-ray determination of the compound were collected on a Bruker D8 QUEST instrument at 273 K with MoKα radiation (λ = 0.71073 Å) using a TRIUMPH monochromator. Thin-layer chromatography (TLC) using precoated TLC sheets (Merck 60F254) were selected to monitor progress of the reaction, and UV lamp was used at 254 nm to visualize spots. Multiplicities are shown as follows: s (singlet), s broad (singlet broad), d (doublet), t (triplet), m (multiplet), q (quartet). Column chromatography was conducted using silica gel 60 (0.04–0.06 mm). Solvents were commercially available materials of reagent grade. The detail of synthesis experiments are given in Supplementary information S1.

### Cell lines and cell culture

For Hepatocarcinoma (HepG2), human lung carcinoma (LU-1), human colorectal adenocarcinoma (SW480) and human normal embryonic kidney (HEK-293) cell lines, the culture medium was Dulbecco’s Modified Eagle’s Medium (DMEM) (Hyclone, USA). The medium supplementation included 10% fetal bovine serum (Hyclone), 100 units/ml penicillin, and 100 μg/ml streptomycin at 37 °C in an atmosphere with 5% CO2 and 95% humidity. For the human leukemia (HL-60) cell line, the maintenance was performed in RPMI-1640 medium (Life Technologies, Inc.) supplemented with 10% heat-inactivated fetal bovine serum (Hyclone) and 100 units/ml penicillin, and 100 μg/ml streptomycin.

### Cell viability assay

Cell viability assay was conducted using SRB method for HepG2, LU-1, SW480 and HEK-293 cell lines, based on the measurement of protein content^35^. Cells were plated with 180 μl growth medium into 96-microwell plates (4 × 10^4^ cells per well) and allowed to grow overnight. Test compounds at various concentrations including 20 μM; 4 μM; 0.8 μM; 0.16 μM with incubation medium were supplemented and cultivated for additional 48 h in the same conditions. Afterwards, the removal of the medium was performed and cold 20% (wt/vol) TCA was used to fix the cell monolayer attached to the wall for 1 h at 4 °C, followed by staining with 1X SRB staining solution at room temperature for 30 min and the removal of the residual dye via repeated washing with 1% (v/v) acetic acid. For OD determination at 515 nm, an ELISA Plate Reader (Bio-Rad) was utilized to analyze the protein-bound dye, which dissolved in 10 mM Tris base solution. The blank sample and the positive sample were DMSO 10% and indirubin-3′-oxime respectively. To represent cytotoxicity, half inhibition concentration (IC50) was calculated by Table Curve 4.0 using data from triplicate experiments at different concentrations of 20 μM; 4 μM; 0.8 μM and 0.16 μM. The cell survival rate (SR) was calculated as SR = [(ODt − OD_0_)/(ODc − OD_0_)] × 100, where the subscript 0, t and c represent time-zero, day three and the DMSO control sample.

For HL-60 cell line, 5 × 10^3^ cells/well were seeded in 96-multiwell plate and cultured overnight. The test compounds were serially diluted to the wells with concentrations ranging at 20 μM; 4 μM; 0.8 μM and 0.16 μM then incubated for 72 h. Thereafter, each well was added with 10 μl of MTT (5 mg/ml in 1X PBS), followed by incubation for 3 h and centrifugation at 1,500 rpm for 10 min. The media were discarded then added 150 μl DMSO to each well. Optical density was measured using a microplate reader at 540 nm. IC_50_ values were calculated as the concentrations that show 50% inhibition of proliferation on the HL-60 cell line.

### Ligands and receptor preparation

Crystal structure of GSK-3β (PDB ID: 1Q41) was employed for the docking studies^11,36^. The 3D structure of protein was obtained from the Protein Data Bank (PDB) and prepared using Autodock Tools (ADT). The designed molecules (ligands) were prepared using MarvinSketch version 19.27.0 and PyMOL version 2.2.2^37^. The energy minimization was carried out using Gabedit version 2.5.0. The Molinspiration and ProTox-II cheminformatic server were utilized to predict bioactivity and assess the toxicity of all research compounds.

### Docking using AutoDock4

The docking simulation procedure was performed by AutoDock 4.2.6 utilizing Lamarckian genetic algorithm and an empirical binding free energy function. Docking modes produced by this procedure are expected to agree with X-ray crystal structures^38,39^. In addition, the method also allows for simulation of interactions between candidate compounds and their three dimensional structures receptors, thereby improving ligand flexibility. The detail of molecular docking simulation is given in Supplementary information S2.

### FPL simulations

**Figure 6:**
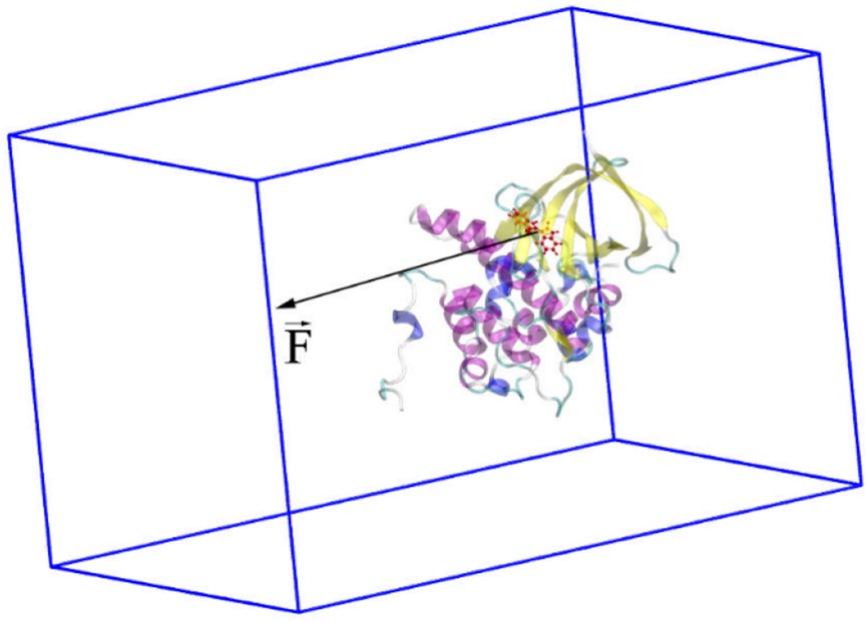
Computational modeling of FPL calculations. The pulling pathway is aligned along Z-axis. The solvation was hidden for clarifier view.

Structure of complexes was obtained via molecular docking method. Caver 2.1^40^ was employed to evaluate the disassociate direction of a ligand referring previous works^30^. All complexes were then aligned to be the disassociate pathway oriented to the Z-axis. The complex was inserted into a periodic boundary condition rectangular box (8.15 × 8.21 × 12.75 nm), which consists of an enzyme GSK-3β, a ligand, 26,000 water molecules and 9 Cl- ions as shown in Fig. 6. In particular, the protein was parameterized via the AMBER99SB-ILDN force field^41^, and the ligand was represented using general Amber force field^42^. The water molecule was topologized using TIP3P water model^43^.

GROMACS version 5.1.5^44^ was employed to carry out the molecular dynamics (MD) simulations. The simulation was performed according to the following four steps: energic minimization, NVT, NPT, and steered-MD (SMD) simulations. In particular, the non-covalent pair was affected within a range of 0.9 nm and the pair list was updated every 5 fs. The Particle mesh Ewald method^45^ was employed to mimic the electrostatic interaction with cut-off of 0.9 nm. The van der Waals interaction was affected in a range of 0.9 nm. Both of NVT and NPT simulations were carried out with the length of 100 ps at 300 K. During simulations, the GSK-3β C_α atoms were restrained by using a weak harmonic force of 1,000 kJ/mol nm^2^. The last snapshot of NPT simulations was used as initial structure of SMD simulations. In this scheme, a harmonic-external force with cantilever spring constant of k = 600 kJ/mol/nm^2^ and pulling speed v = 0.005 nm/ps was put on the ligand center of mass along the Z-direction. The ligand was disassociated out of the binding site of GSK-3β enzyme over SMD simulations. The data was recorded every 0.1 ps during SMD simulations. The calculations were independently repeated 8 times with the same initial conformation to guarantee the sampling of calculation.

### Statistical analysis

All data are presented as mean ± SD. Experiments were carried out in triplicate for the accuracy of data. Statistical significant differences were realized at *p* < 0.05 via Student’s *t* test.

## Supplementary information

Supplementary information

## Data Availability

Supplementary data in this article are provided in Supporting information. ^1^H NMR and ^13^C NMR data and spectrums of compounds **3**, **4**, **6a–p** were available free of charge via the Supporting Information.
