# Supplementary material for: Design, synthesis, structure, in vitro cytotoxic activity evaluation and docking studies on target enzyme GSK-3β of new indirubin-3ʹ-oxime derivatives
Source: Sci Rep. 2020 Jul 10;10:11429. doi: 10.1038/s41598-020-68134-8 (PMC7351726; doi:10.1038/s41598-020-68134-8)
Supplement: Supplementary file 1 — Supplementary information [file 41598_2020_68134_MOESM1_ESM.pdf]

Design, synthesis, structure, *in vitro* cytotoxic activity evaluation and docking studies on target enzyme GSK-3β of new indirubin-3′-oxime derivatives

Nguyen Trong Dan[1,2,3], Hoang Duc Quang[3], Vuong Van Truong[3], Do Huu Nghi[1,2], Nguyen Manh Cuong[1,2], To Dao Cuong[4,5], Tran Quoc Toan[1,2], Long Giang Bach[6,7], Nguyen Huu Thuan Anh[6,7], Nguyen Thi Mai[8,9], Ngo Thi Lan[2,10], Luu Van Chinh[1,2,], Pham Minh Quan[1,2*]*

[1]Institute of Natural Products Chemistry, Vietnam Academy of Science and Technology, 18 Hoang Quoc Viet, Cau Giay, Hanoi 11307, Vietnam
[2]Graduate University of Science and Technology, Vietnam Academy of Science and Technology, 18 Hoang Quoc Viet, Cau Giay, Hanoi 11307, Vietnam
[3]Vietnam-Russia Tropical Center, Nguyen Van Huyen, Nghia Do, Cau Giay, Hanoi 11307, Vietnam
[4]Faculty of Pharmacy, Phenikaa University, Yen Nghia, Ha Dong, Hanoi 12116, Vietnam
[5]Phenikaa Research and Technology Institute (PRATI), A&A Green Phoenix Group JSC, No.167 Hoang Ngan, Trung Hoa, Cau Giay, Hanoi 11313, Vietnam
[6]NTT Hi-Tech Institute, Nguyen Tat Thanh University, Ho Chi Minh City, Vietnam
[7]Center of Excellence for Functional Polymers and NanoEngineering, Nguyen Tat Thanh University, Ho Chi Minh City, Vietnam
[8]Laboratory of Theoretical and Computational Biophysics, Ton Duc Thang University, Ho Chi Minh City, Vietnam
[9]Faculty of Applied Sciences, Ton Duc Thang University, Ho Chi Minh City, Vietnam
[10]Institute of Materials Science, Vietnam Academy of Science and Technology, 18 Hoang Quoc Viet, Cau Giay, Hanoi 11307, Vietnam
**Corresponding authors: PHAM Minh Quan at Institute of Natural Products Chemistry – Vietnam Academy of Science and Technology*
E-mail: pham-minh.quan@inpc.vast.vn and chinhluuvan@gmail.com; Tel : +84987481589

S.No.	Contents	Page No.
**1.**	General synthesis procedures	2-8
**2.**	Docking using AutoDock4	9
**3.**	X-Ray crystallography data	10-12
**4.**	Detailed description for docking studies	13-18
**5.**	FPL Simulations	19-24
**6.**	Spectroscopic data	25-65
**7.**	References	66

S1. General synthesis procedures

Synthesis of (2′*Z*)-1-(prop-2-ynyl)indirubin (3): Solution of compound 1 (15.27 mmol, 4g), propagyl bromide (45.81 mmol, 3.47 mL), anhydrous K_2CO_3 (45.81 mmol, 6.33 mg), KI (1.53 mmol, 254 mg), (1-butyl)triethylammonium bromide (1.53 mmol, 365 mg) in dried DMF (250 mL) was stirred at room temperature for 48 hours. Upon completion of the reaction, the mixture was poured into 1.5 L of distilled water, stirred and let stand for 1 hour. Then, filter the crude product through a Buchner funnel, washed the crude product 3 times x 100 mL of distilled water. Dry the crude product at 80°C for 24 hours and chromatographed on silicagel 60, 0.04÷0.06 mm, eluting with n-hexane/ acetone (4/1, v/v) to give the corresponding compound **3**.

(2′*Z*)-1-(prop-2-ynyl)indirubin (3): Yield 59 %, dark violet crystal, m.p. 245÷246 ^{0}C. ^{1}H NMR (DMSO-d_6, 500 MHz), δ(ppm): 11.09 (s, 1H, H-1′); 8.82 (d, J = 7.5 Hz, 1H, H-4); 7.66 (d, J = 7.5 Hz, 1H, H-4′); 7.59 (t, J = 7.75 Hz, 1H, H-6′); 7.42 (d, J = 8 Hz, 1H, H-7′); 7.37 (t, J = 7.75 Hz, 1H, H-6); 7.17 (d, J = 8 Hz, 1H, H-7); 7.13 (t, J = 7.5 Hz, 1H, H-5); 7.04 (t, J = 7.5 Hz, 1H, H-5′); 4.68 (d, J = 2.5 Hz, 2H, CH_2); 3.29 (t, J = 2.25 Hz, 1H, $CH_{propagyl}$).^{13}C NMR (DMSO-d_6, 125 MHz), δ(ppm): 188.5 (C-3′); 168.2 (C-2); 152.5 (C-7′a); 140 (C-7a); 139.1 (C-2′); 137.3 (C-6′); 129 (C-6); 124.5 (C-4, C-4′); 122.2 (C-3a); 121.6 (C-5); 120.8 (C-5′); 119.1 (C-3′a); 113.6 (C-7′); 109 (C-7); 104.8 (C-3); 78.2 ($C_{propagyl}$); 74.3 ($CH_{propagyl}$); 28.6 (CH_2). HR-MS(ESI): caculated for $C_{19}H_{13}N_2O_2[M+H]^+$: 301.0977, found: 301.0969.

Synthesis of (2'*Z*, 3'*E*)-1-(prop-2-ynyl)indirubin-3′-oxime (4): Solution of compound 3 (4 mmol, 1.2 g), hydroxylamine chlohydric (12 mmol, 834 mg) in 25 mL pyridine was heated to reflux for 3 hours. At the end of the reaction, the mixture is cooled to 5°C, followed by stirring and slow addition of 330 mL of 0.1 M HCl. The precipitate was filtered on the Buchner funnel and recrystallized in the solvent system n-hexane acetone (2/1, v / v) to give the pure product 4.

(2'*Z*, 3'*E*)-1-(prop-2-ynyl)indirubin-3′-oxime (4): Yield 79 %, red crystal, m.p. 257÷258 ^{o}C. ^{1}H NMR (DMSO-d_6, 500 MHz), δ(ppm): 13.61 (s, 1H, OH_{oxime}); 11.71 (s, 1H, H-1′); 8.72 (d, J = 8 Hz, 1H, H-4); 8.24 (d, J = 8 Hz, 1H, H-4′); 7.44÷7.4 (m, 2H, H-6′, H-7′); 7.25 (t, J = 7.5 Hz, 1H, H-6); 7.15 (d, J = 8 Hz, 1H, H-7); 7.08÷7.04 (m, 2H, H-5′, H-5); 4.71 (d, J = 2.5 Hz, 2H, CH_2); 3.25 (t, J = 2 Hz, 1H, $CH_{propagyl}$).^{13}C NMR (DMSO-d_6, 125 MHz), δ(ppm): 168.1 (C-2); 151.2 (C-3′); 146.1 (C-2'); 144.7 (C-7′a); 137.6 (C-7a); 132.1 (C-6′); 127.9 (C-4′); 125.8 (C-6); 122.9 (C-4); 122 (C-3a); 121.8 (C-5′); 121.3 (C-5); 116.5 (C-3′a); 111.8 (C-7′); 108.3 (C-7); 97.3 (C-3); 78.7 ($C_{propagyl}$); 74 ($CH_{propagyl}$); 28.4 (CH_2). HR-MS(ESI): caculated for $C_{19}H_{14}N_3O_2[M+H]^+$: 316.1086, found: 316.1073. Crystal data for **4** were deposited at CCDC with reference CCDC 1917485: Empirical formula $C_{19}H_{13}N_3O_2$, Formual weight = 315.32, Temperature (K) = 273, Wavelength (Å) = 0.71073, Crystal system Orthorhombic, Space group Pbca, Unit cell dimensions a, b, c (Å) 16.5300(13), 9.3791(8), 19.7270(18) α, β, γ (^{o}) 90, 90, 90. Volume (Å^3) = 3058.4(5), Z = 8, Density (Mg/m^3) = 1.370, Absorption coefficient (mm^{-1}) = 0.092, F(000) = 1312, Crystal size (mm^3) = 0.138 × 0.060 × 0.035, Theta range for data collection from 3.216 to 25.732^o, Index ranges h = -20→20, k = -11→11, l = -24→24, Reflections collected 36804, Independent reflections 2915 [R(int)=0.0860], Max. and min. transmission 0.7453 and 0.6984, Refinement method Full-matrix least-squares on F^2, Final R indices [I>2sigma(I)] R1 = 0.0446, wR2 = 0.0958, R indices (all data) R1 =

0.0898, wR2 = 0.1178, Largest diff peak and hole (e Å^{-3}) 0.154 and -0.192 (Table S1). Selected bond distances and angles are listed in Table S2.

Synthesis of indirubin-3′-oxime derivatives (6a-l): Solution of compound **4** (0.5 mmol, 158 mg) and corresponding compound **5a-l** (0.6 mmol) and CuI (0.025 mmol, 4.8 mg) in 5 ml DMSO was stirred at room temperature for 24 hours. After the reaction was completed, the mixture is diluted with 100 mL of ethyl acetate, wash 3 times x 100 mL of Na_2SO_4 3%. The ethyl acetate solution was then dried with anhydrous Na_2SO_4 and solvent removed under reduced pressure, the crude product was then chromatographed on silicagel 60, 0.04÷0.06 mm, eluting with n-hexane/ acetone (3/2, v/v) and give the corresponding compounds **6a-l**.

(2’*Z*, 3’*E*)-1-((1-(2-((*E*)-2’’’-hydroxychalcon-4’’’-oxy)ethyl)-1*H*-1,2,3-triazol-4-yl)methyl)indirubin-3′-oxime (6a): Yield 63 %, red solid, m.p. 227÷228 °C. ^{1}H NMR (DMSO-d_6, 500 MHz), δ(ppm): 13.57 (s, 1H, OH_{oxime}); 13.35 (s, 1H, $OH_{chalcone}$); 11.78 (s, 1H, H-1′); 8.7 (d, *J* = 8 Hz, 1H, H-4); 8.23 (m, 2H, H-6‴, H-4′); 8.12 (s, 1H, H-5″); 8.00 (d, *J* = 15.5 Hz, 1H, H-α); 7.92 (m, 2H, H-2⁗, H-6⁗); 7.83 (d, *J* = 15.5 Hz, 1H, H-β); 7.48 (m, 3H, H-4⁗, H-3⁗, H-5⁗); 7.42 (m, 2H, H-6′, H-7′); 7.18÷7.13 (m, 2H, H-6, H-7); 7.05÷6.99 (m, 2H, H-5′, H-5); 6.51÷6.49 (m, 2H, H-3‴, H-5‴); 5.15 (s, 2H, CH_2-C-4″); 4.74 (t, *J* = 4.75 Hz, 2H, -C$\underline{H}_2$-CH_2-O-); 4.49 (t, *J* = 4.75 Hz, 2H, -CH_2-C$\underline{H}_2$-O-). ^{13}C NMR (DMSO-d_6, 125 MHz), δ(ppm): 191.9 (C=O); 168.6 (C-2); 165.5 (C-2‴); 164.4 (C-4‴); 151.3 (C-3′); 145.8 (C-2′); 144.7 (C-7′a); 144.3 (C-β); 142.8 (C-4″); 138.3 (C-7a); 134.5 (C-1⁗); 132.8 (C-6‴); 132.1 (C-6′); 130.9 (C-4⁗); 129.1 (C-2⁗, C-6⁗); 128.9 (C-3⁗, C-5⁗); 127.9 (C-4′); 125.7 (C-6); 123.9 (C-5″); 122.8 (C-4); 122 (C-3a); 121.7 (C-5′); 121.2 (C-α); 121.0 (C-5); 116.5 (C-3′a); 114.3 (C-1‴); 111.6 (C-7′); 108.3 (C-7); 107.6 (C-5‴); 101.7 (C-3‴); 97.7 (C-3); 66.5 (-CH_2-$\underline{C}H_2$-O-); 48.7 (-$\underline{C}H_2$-CH_2-O-); 34.5 ($\underline{C}H_2$ - C-4″). HR-MS(ESI): calculated for $C_{36}H_{29}N_6O_5[M+H]^+$: 625.2199, found: 625.2187.

(2’*Z*, 3’*E*)-1-((1-(2-((*E*)-2’’’-hydroxy-3’’’’,4’’’’, 5’’’’-trimethoxychalcon-4’’’-oxy)ethyl)-1*H*-1,2,3-triazol-4-yl)methyl)indirubin-3′-oxime (6b): Yield 70 %, red solid, m.p. 180÷181 °C. ^{1}H NMR (DMSO-d_6, 500 MHz), δ(ppm): 13.57 (s broad, 1H, OH_{oxime}); 13.49 (s, 1H, $OH_{chalcone}$); 11.77 (s, 1H, H-1′); 8.70 (d, *J* = 8 Hz, 1H, H-4); 8.27 (d, *J* = 9.5 Hz, 1H, H-6‴); 8.23 (d, *J* = 8 Hz, 1H, H-4′); 8.12 (s, 1H, H-5″); 7.93 (d, *J* = 15.5 Hz, 1H, H-α); 7.79 (d, *J* = 15.5 Hz, 1H, H-β); 7.41 (m, 2H, H-6′, H-7′); 7.26 (s, 2H, H-2⁗, H-6⁗); 7.16÷7.12 (m, 2H, H-6, H-7); 7.05÷6.99 (m, 2H, H-5′, H-5); 6.52÷6.5 (m, 2H, H-3‴, H-5‴); 5.15 (s, 2H, CH_2-C-4″); 4.74 (t, *J* = 5 Hz, 2H, -C$\underline{H}_2$-CH_2-O-); 4.49 (t, *J* = 4.75 Hz, 2H, -CH_2-C$\underline{H}_2$-O-); 3.87 (s, 6H, CH_3O-C-3⁗, CH_3O-C-5⁗); 3.72 (s, 3H, CH_3O-C-4⁗).^{13}C NMR (DMSO-d_6, 125 MHz), δ(ppm): 192 (C=O); 168.7 (C-2); 165.6 (C-2‴); 164.4 (C-4‴); 153.2 (C-3⁗, C-5⁗); 151.3 (C-3′); 145.8 (C-2′); 144.9 (C-β); 144.7 (C-7′a); 142.8 (C-4″); 140.1 (C-4⁗); 138.3 (C-7a); 132.8 (C-6‴); 132.1 (C-6′); 130.0 (C-1⁗); 128.0 (C-4′); 125.8 (C-6); 123.8 (C-5″); 122.8 (C-4); 122 (C-3a); 121.7 (C-5′); 121.1 (C-5); 120.2 (C-α); 116.5 (C-3′a); 114.2 (C-1‴); 111.6 (C-7′); 108.3 (C-7); 107.5 (C-5‴); 106.9 (C-2⁗, C-6⁗); 101.7 (C-3‴); 97.7 (C-3); 66.5 (-CH_2-$\underline{C}H_2$-O-); 60.2 ($\underline{C}H_3$O-C-4⁗); 56.2 ($\underline{C}H_3$O-C-3⁗, $\underline{C}H_3$O-C-5⁗); 48.8 (-$\underline{C}H_2$-CH_2-O-); 34.5 ($\underline{C}H_2$-C-4″). HR-MS(ESI): calculated for $C_{39}H_{35}N_6O_8[M+H]^+$: 715.2516, found: 715.2505.

(2’*Z*, 3’*E*)-1-((1-(2-((*E*)-2’’’-hydroxy-2’’’’-methoxychalcon-4’’’-oxy)ethyl)-1*H*-1,2,3-triazol-4-yl)methyl)indirubin-3′-oxime (6c): Yield 60 %, red solid, m.p. 235÷236 °C. ^{1}H NMR (DMSO-d_6, 500 MHz), δ(ppm): 13.57 (s, 1H, OH_{oxime}); 13.39 (s, 1H, $OH_{chalcone}$); 11.78 (s, 1H, H-1′); 8.7 (d, *J* = 8 Hz, 1H, H-4); 8.23 (d, *J* = 7.5 Hz, 1H, H-4′);

8.18 (d, J = 9 Hz, 1H, H-6‴); 8.15 (d, J = 15.5 Hz, 1H, H-α); 8.11 (s, 1H, H-5″); 8.01 (d, J = 8 Hz, 1H, H-3⁗); 7.93 (d, J = 15.5 Hz, 1H, H-β); 7.49÷7.39 (m, 3H, H-6′, H-7′, H-6⁗); 7.18÷7.12 (m, 3H, H-6, H-7, H-5⁗); 7.06÷6.99 (m, 3H, H-4⁗, H-5′, H-5); 6.50÷6.48 (m, 2H, H-3‴, H-5‴); 5.15 (s, 2H, CH_2-C-4″); 4.74 (t, J = 5 Hz, 2H, -C$\underline{H}_2$-CH_2-O-); 4.48 (t, J = 5 Hz, 2H, -CH_2-C$\underline{H}_2$-O-); 3.91 (s, 3H, CH_3). ^{13}C NMR (DMSO-d_6, 125 MHz), δ(ppm): 192 (C=O); 168.6 (C-2); 165.5 (C-2‴); 164.3 (C-4‴); 158.3 (C-2⁗); 151.3 (C-3′); 145.8 (C-2′); 144.7 (C-7′a); 142.8 (C-4″); 138.7 (C-β); 138.3 (C-7a); 132.7 (C-6‴); 132.6 (C-1⁗); 132.1 (C-6′); 128.6 (C-4⁗); 127.9 (C-4′); 125.7 (C-6); 123.8 (C-5″); 122.8 (C-4, C-6⁗); 122 (C-3a); 121.7 (C-5′); 121.0 (C-5); 120.7 (C-α, C-5⁗); 116.5 (C-3′a); 114.3 (C-1‴); 111.9 (C-3⁗); 111.6 (C-7′); 108.3 (C-7); 107.5 (C-5‴); 101.7 (C-3‴); 97.7 (C-3); 66.5 (-CH_2-$\underline{C}H_2$-O-); 55.8 (CH_3); 48.7 (-$\underline{C}H_2$-CH_2-O-); 34.5 ($\underline{C}H_2$-C-4″). HR-MS(ESI): calculated for $C_{37}H_{31}N_6O_6[M+H]^+$: 655.2305, found: 655.2288.

(2'*Z*, 3'*E*)-1-((1-(2-((*E*)-2'''-hydroxy-3''''-methoxychalcon-4'''-oxy)ethyl)-1*H*-1,2,3-triazol-4-yl)methyl)indirubin-3′-oxime (6d): Yield 65 %, red solid, m.p. 211÷212 ^{o}C. ^{1}H NMR (DMSO-d_6, 500 MHz), δ(ppm): 13.57 (s, 1H, OH_{oxime}); 13.36 (s, 1H, $OH_{chalcone}$); 11.78 (s, 1H, H-1′); 8.7 (d, J = 8 Hz, 1H, H-4); 8.27 (d, J = 9 Hz, 1H, H-6‴); 8.23 (d, J = 8 Hz, 1H, H-4′); 8.12 (s, 1H, H-5″); 8.00 (d, J = 15.5 Hz, 1H, H-α); 7.79 (d, J = 15,5 Hz, 1H, H-β); 7.51 (s, 1H, H-2⁗); 7.46 (d, J = 8 Hz, 1H, H-5⁗); 7.42÷7.37 (m, 3H, H-6′, H-7′, H-6⁗); 7.18÷7.12 (m, 2H, H-6, H-7); 7.06÷6.99 (m, 3H, H-4⁗, H-5′, H-5); 6.51÷6.49 (m, 2H, H-3‴, H-5‴); 5.15 (s, 2H, CH_2-C-4″); 4.74 (t, J = 4.75 Hz, 2H, -C$\underline{H}_2$-CH_2-O-); 4.49 (t, J = 5 Hz, 2H, -CH_2-C$\underline{H}_2$-O-); 3.84 (s, 3H, CH_3). ^{13}C NMR (DMSO-d_6, 125 MHz), δ(ppm): 192 (C=O); 168.6 (C-2); 165.5 (C-2‴); 164.4 (C-4‴); 159.7 (C-3⁗); 151.3 (C-3′); 145.8 (C-2′); 144.7 (C-7′a); 144.3 (C-β); 142.8 (C-4″); 138.3 (C-7a); 135.9 (C-1⁗); 132.9 (C-6‴); 132.1 (C-6′); 129.9 (C-5⁗); 127.9 (C-4′); 125.7 (C-6); 123.8 (C-5″); 122.8 (C-4); 122 (C-3a, C-6⁗); 121.7 (C-5′); 121.4 (C-α); 121.0 (C-5); 116.9 (C-4⁗); 116.5 (C-3′a); 114.2 (C-1‴); 113.6 (C-2⁗); 111.7 (C-7′); 108.3 (C-7); 107.6 (C-5‴); 101.7 (C-3‴); 97.7 (C-3); 66.5 (-CH_2-$\underline{C}H_2$-O-); 55.4 (CH_3); 48.7 (-$\underline{C}H_2$-CH_2-O-); 34.5 ($\underline{C}H_2$-C-4″). HR-MS(ESI): calculated for $C_{37}H_{31}N_6O_6[M+H]^+$: 655.2305, found: 655.2297.

(2'*Z*, 3'*E*)-1-((1-(2-((*E*)-2'''-hydroxy-4''''-methoxychalcon-4'''-oxy)ethyl)-1*H*-1,2,3-triazol-4-yl)methyl)indirubin-3′-oxime (6e): Yield 69 %, red solid, m.p. 256÷257 ^{o}C. ^{1}H NMR (DMSO-d_6, 500 MHz), δ(ppm): 13.57 (s, 1H, OH_{oxime}); 13.53 (s, 1H, $OH_{chalcone}$); 11.78 (s, 1H, H-1′); 8.7 (d, J = 8 Hz, 1H, H-4); 8.23 (m, 2H, H-6‴, H-4′); 8.12 (s, 1H, H-5″); 7.89÷7.85 (m, 3H, H-2⁗, H-6⁗, H-α); 7.8 (d, J = 15 Hz, 1H, H-β); 7.41 (m, 2H, H-6′, H-7′); 7.18÷7.13 (m, 2H, H-6, H-7); 7.06÷6.99 (m, 4H, H-3⁗, H-5⁗, H-5′, H-5); 6.49÷6.47 (m, 2H, H-3‴, H-5‴); 5.15 (s, 2H, CH_2-C-4″); 4.74 (t, J = 5 Hz, 2H, -C$\underline{H}_2$-CH_2-O-); 4.48 (t, J = 5 Hz, 2H, -CH_2-C$\underline{H}_2$-O-); 3.83 (s, 3H, CH_3). ^{13}C NMR (DMSO-d_6, 125 MHz), δ(ppm): 191.9 (C=O); 168.6 (C-2); 165.5 (C-2‴); 164.2 (C-4‴); 161.6 (C-4⁗); 151.3 (C-3′); 145.8 (C-2′); 144.8 (C-7′a); 144.5 (C-β); 142.8 (C-4″); 138.3 (C-7a); 132.6 (C-6‴); 132.1 (C-6′); 131.2 (C-2⁗, C-6⁗); 127.9 (C-4′); 127.2 (C-1⁗); 125.8 (C-6); 123.9 (C-5″); 122.8 (C-4); 122 (C-3a); 121.7 (C-5′); 121.1 (C-5); 118.4 (C-α); 116.5 (C-3′a); 114.5 (C-3⁗, C-5⁗); 114.2 (C-1‴); 111.7 (C-7′); 108.3 (C-7); 107.4 (C-5‴); 101.7 (C-3‴); 97.7 (C-3); 66.5 (-CH_2-$\underline{C}H_2$-O-); 55.5 (CH_3); 48.8 (-$\underline{C}H_2$-CH_2-O-); 34.5 ($\underline{C}H_2$-C-4″). HR-MS(ESI): calculated for $C_{37}H_{31}N_6O_6[M+H]^+$: 655.2305, found: 655.2283.

(2'*Z*, 3'*E*)-1-((1-(2-((*E*)-2''''-ethoxy-2'''-hydroxychalcon-4'''-oxy)ethyl)-1*H*-1,2,3-triazol-4-yl)methyl)indirubin-3′-oxime (6f): Yield 66 %, red solid, m.p. 213÷214 ^{o}C. ^{1}H NMR (DMSO-d_6, 500 MHz), δ(ppm): 13.56 (s, 1H, OH_{oxime}); 13.37 (s, 1H, $OH_{chalcone}$);

11.78 (s, 1H, H-1′); 8.70 (d, *J* = 8 Hz, 1H, H-4); 8.23 (d, *J* = 8 Hz, 1H, H-4′); 8.16÷8.11 (m, 3H, H-6‴, H-α, H-5″); 7.99 (d, *J* = 7.5 Hz, 1H, H-3⁗); 7.96 (d, *J* = 15.5 Hz, 1H, H-β); 7.46÷7.41 (m, 3H, H-6′, H-7′, H-6⁗); 7.16÷7.1 (m, 3H, H-6, H-7, H-5⁗); 7.05÷6.99 (m, 3H, H-4⁗, H-5′, H-5); 6.50÷6.49 (m, 2H, H-3‴, H-5‴); 5.15 (s, 2H, CH_2-C-4″); 4.74 (t, *J* = 4.75 Hz, 2H, -C$\underline{H}_2$-CH_2-O-); 4.48 (t, *J* = 4.75 Hz, 2H, -CH_2-C$\underline{H}_2$-O-); 4.16 (q, *J* = 6.75 Hz, 2H, -O-C$\underline{H}_2$-CH_3); 1.42 (t, *J* = 7 Hz, 3H, -O-CH_2-C$\underline{H}_3$). ^{13}C NMR (DMSO-d_6, 125 MHz), δ(ppm): 192 (C=O); 168.6 (C-2); 165.5 (C-2‴); 164.3 (C-4‴); 157.7 (C-2⁗); 151.2 (C-3′); 145.8 (C-2′); 144.7 (C-7′a); 142.8 (C-4″); 138.9 (C-β); 138.3 (C-7a); 132.6 (C-6‴); 132.4 (C-1⁗); 132.1 (C-6′); 128.9 (C-4⁗); 127.9 (C-4′); 125.7 (C-6); 123.8 (C-5″); 122.8 (C-4, C-6⁗); 122 (C-3a); 121.7 (C-5′); 121.0 (C-5); 120.7 (C-α); 120.6 (C-5⁗); 116.5 (C-3′a); 114.3 (C-1‴); 112.7 (C-3⁗); 111.6 (C-7′); 108.3 (C-7); 107.5 (C-5‴); 101.7 (C-3‴); 97.7 (C-3); 66.5 (-CH_2-$\underline{C}H_2$-O-); 63.8 (-O-$\underline{C}H_2$-CH_3); 48.7 (-$\underline{C}H_2$-CH_2-O-); 34.5 ($\underline{C}H_2$-C-4″); 14.6 (-O-CH_2-$\underline{C}H_3$). HR-MS(ESI): calculated for $C_{38}H_{33}N_6O_6[M+H]^+$: 669.2461, found: 669.2453.

(2′*Z*, 3′*E*)-1-((1-(2-((*E*)-3⁗-ethoxy-2‴-hydroxychalcon-4‴-oxy)ethyl)-1*H*-1,2,3-triazol-4-yl)methyl)indirubin-3′-oxime (6g): Yield 57 %, red solid, m.p. 195÷196 °C. ^{1}H NMR (DMSO-d_6, 500 MHz), δ(ppm): 13.56 (s, 1H, OH_{oxime}); 13.37 (s, 1H, $OH_{chalcone}$); 11.77 (s, 1H, H-1′); 8.7 (d, *J* = 8 Hz, 1H, H-4); 8.27 (d, *J* = 9 Hz, 1H, H-6‴); 8.23 (d, *J* = 7.5 Hz, 1H, H-4′); 8.12 (s, 1H, H-5″); 8.00 (d, *J* = 15.5 Hz, 1H, H-α); 7.78 (d, *J* = 15.5 Hz, 1H, H-β); 7.51 (s, 1H, H-2⁗); 7.44÷7.35 (m, 4H, H-5⁗, H-6⁗, H-6′, H-7′); 7.18÷7.12 (m, 2H, H-6, H-7); 7.05÷6.99 (m, 3H, H-4⁗, H-5′, H-5); 6.5÷6.49 (m, 2H, H-3‴, H-5‴); 5.15 (s, 2H, CH_2-C-4″); 4.74 (t, *J* = 4.75 Hz, 2H, -C$\underline{H}_2$-CH_2-O-); 4.49 (t, *J* = 5 Hz, 2H, -CH_2-C$\underline{H}_2$-O-); 4.11 (q, *J* = 6.75 Hz, 2H, -O-C$\underline{H}_2$-CH_3); 1.36 (t, *J* = 7 Hz, 3H, -O-CH_2-C$\underline{H}_3$). ^{13}C NMR (DMSO-d_6, 125 MHz), δ(ppm): 192 (C=O); 168.6 (C-2); 165.5 (C-2‴); 164.4 (C-4‴); 158.9 (C-3⁗); 151.2 (C-3′); 145.8 (C-2′); 144.7 (C-7′a); 144.4 (C-β); 142.8 (C-4″); 138.3 (C-7a); 135.8 (C-1⁗); 132.9 (C-6‴); 132.1 (C-6′); 129.9 (C-5⁗); 127.9 (C-4′); 125.7 (C-6); 123.8 (C-5″); 122.8 (C-4); 122 (C-3a, C-6⁗); 121.7 (C-5′); 121.4 (C-α); 121.0 (C-5); 117.2 (C-4⁗); 116.5 (C-3′a); 114.2 (C-1‴); 114.1 (C-2⁗); 111.6 (C-7′); 108.3 (C-7); 107.5 (C-5‴); 101.7 (C-3‴); 97.7 (C-3); 66.5 (-CH_2-$\underline{C}H_2$-O-); 63.2 (-O-$\underline{C}H_2$-CH_3); 48.7 (-$\underline{C}H_2$-CH_2-O-); 34.5 ($\underline{C}H_2$-C-4″); 14.6 (-O-CH_2-$\underline{C}H_3$). HR-MS(ESI): calculated for $C_{38}H_{33}N_6O_6[M+H]^+$: 669.2461, found: 669.2447.

(2′*Z*, 3′*E*)-1-((1-(2-((*E*)-4⁗-ethoxy-2‴-hydroxychalcon-4‴-oxy)ethyl)-1*H*-1,2,3-triazol-4-yl)methyl)indirubin-3′-oxime (6h): Yield 68 %, red solid, m.p. 196÷197 °C. ^{1}H NMR (DMSO-d_6, 500 MHz), δ(ppm): 13.57 (s, 1H, OH_{oxime}); 13.53 (s, 1H, $OH_{chalcone}$); 11.78 (s, 1H, H-1′); 8.7 (d, *J* = 7.5 Hz, 1H, H-4); 8.23 (d, *J* = 9 Hz, 2H, H-6‴, H-4′); 8.12 (s, 1H, H-5″); 7.88÷7.84 (m, 3H, H-2⁗, H-6⁗, H-α); 7.8 (d, *J* = 15 Hz, 1H, H-β); 7.42 (m, 2H, H-6′, H-7′); 7.18÷7.13 (m, 2H, H-6, H-7); 7.06÷6.99 (m, 4H, H-3⁗, H-5⁗, H-5′, H-5); 6.49÷6.47 (m, 2H, H-3‴, H-5‴); 5.15 (s, 2H, CH_2-C-4″); 4.74 (t, *J* = 4.75 Hz, 2H, -C$\underline{H}_2$-CH_2-O-); 4.48 (t, *J* = 4.75 Hz, 2H, -CH_2-C$\underline{H}_2$-O-); 4.11 (q, *J* = 7 Hz, 2H, -O-C$\underline{H}_2$-CH_3); 1.35 (t, *J* = 6.75 Hz, 3H, -O-CH_2-C$\underline{H}_3$). ^{13}C NMR (DMSO-d_6, 125 MHz), δ(ppm): 191.9 (C=O); 168.6 (C-2); 165.5 (C-2‴); 164.2 (C-4‴); 160.9 (C-4⁗); 151.2 (C-3′); 145.8 (C-2′); 144.7 (C-7′a); 144.5 (C-β); 142.8 (C-4″); 138.3 (C-7a); 132.6 (C-6‴); 132.1 (C-6′); 131.2 (C-2⁗, C-6⁗); 127.9 (C-4′); 127 (C-1⁗); 125.7 (C-6); 123.8 (C-5″); 122.8 (C-4); 122 (C-3a); 121.7 (C-5′); 121 (C-5); 118.3 (C-α); 116.5 (C-3′a); 114.8 (C-3⁗, C-5⁗); 114.2 (C-1‴); 111.6 (C-7′); 108.3 (C-7); 107.4 (C-5‴); 101.7 (C-3‴); 97.7 (C-3); 66.5 (-CH_2-$\underline{C}H_2$-O-

); 63.4 (-O-CH_2-CH_3); 48.7 (-CH_2-CH_2-O-); 34.5 (CH_2-C-4″); 14.5 (-O-CH_2-CH_3). HR-MS(ESI): calculated for $C_{38}H_{33}N_6O_6[M+H]^+$: 669.2461, found: 669.2448.

(2′*Z*, 3′*E*)-1-((1-(2-((*E*)-2‴-hydroxy-2⁗-propoxychalcon-4‴-oxy)ethyl)-1*H*-1,2,3-triazol-4-yl)methyl)indirubin-3′-oxime (6i): Yield 68 %, red solid, m.p. 216÷217 °C. ^{1}H NMR (DMSO-d_6, 500 MHz), δ(ppm): 13.58 (s broad, 1H, OH_{oxime}); 13.32 (s, 1H, $OH_{chalcone}$); 11.75 (s, 1H, H-1′); 8.68 (d, *J* = 8 Hz, 1H, H-4); 8.22 (d, *J* = 7.5 Hz, 1H, H-4′); 8.13 (d, *J* = 15.5 Hz, 1H, H-α); 8.11÷8.10 (m, 2H, H-6‴ , H-5″); 7.96÷7.91 (m, 2H, H-3⁗, H-β); 7.45÷7.38 (m, 3H, H-6′, H-7′, H-6⁗); 7.17÷7.09 (m, 3H, H-6, H-7, H-5⁗); 7.05÷6.98 (m, 3H, H-4⁗, H-5′, H-5); 6.49÷6.48 (m, 2H, H-3‴, H-5‴); 5.14 (s, 2H, CH_2-C-4″); 4.73 (t, *J* = 5 Hz, 2H, -CH_2-CH_2-O-); 4.46 (t, *J* = 5 Hz, 2H, -CH_2-CH_2-O-); 4.05 (t, *J* = 6.5 Hz, 2H, -O-CH_2-CH_2-CH_3); 1.81 (m, 2H, -O-CH_2-CH_2-CH_3); 1.03 (t, *J* = 7.25 Hz, 3H, -O-CH_2-CH_2- CH_3). ^{13}C NMR (DMSO-d_6, 125 MHz), δ(ppm): 192.1 (C=O); 168.7 (C-2); 165.5 (C-2‴); 164.3 (C-4‴); 158.0 (C-2⁗); 151.3 (C-3′); 145.9 (C-2′); 144.8 (C-7′a); 142.9 (C-4″); 139.1 (C-β); 138.4 (C-7a); 132.7 (C-6‴); 132.4 (C-1⁗); 132.2 (C-6′); 129.2 (C-4⁗); 128.0 (C-4′); 125.8 (C-6); 123.9 (C-5″); 122.9 (C-4, C-6⁗); 122.0 (C-3a); 121.8 (C-5′); 121.2 (C-5); 120.9 (C-5⁗); 120.7 (C-α); 116.6 (C-3′a); 114.4 (C-1‴); 112.8 (C-3⁗); 111.7 (C-7′); 108.3 (C-7); 107.6 (C-5‴); 101.8 (C-3‴); 97.8 (C-3); 69.7 (-O-CH_2-CH_2-CH_3); 66.6 (-CH_2-CH_2-O-); 48.8 (-CH_2-CH_2-O-); 34.5 (CH_2-C-4″); 22.1 (-O-CH_2-CH_2-CH_3); 10.6 (-O-CH_2-CH_2-CH_3). HR-MS(ESI): calculated for $C_{39}H_{35}N_6O_6[M+H]^+$: 683.2618, found: 683.2607.

(2′*Z*, 3′*E*)-1-((1-(2-((*E*)-2‴-hydroxy-3⁗-propoxychalcon-4‴-oxy)ethyl)-1*H*-1,2,3-triazol-4-yl)methyl)indirubin-3′-oxime (6k): Yield 59 %, red solid, m.p. 193÷194 °C. ^{1}H NMR (DMSO-d_6, 500 MHz), δ(ppm): 13.61 (s broad, 1H, OH_{oxime}); 13.32 (s broad, 1H, $OH_{chalcone}$); 11.75 (s, 1H, H-1′); 8.68 (d, *J* = 8 Hz, 1H, H-4); 8.24÷8.22 (m, 2H, H-6‴, H-4′); 8.10 (s, 1H, H-5″); 7.95 (d, *J* = 15.5 Hz, 1H, H-α); 7.77 (d, *J* = 15.5 Hz, 1H, H-β); 7.48 (d, *J* = 1.5 Hz, 1H, H-2⁗); 7.42÷7.34 (m, 4H, H-5⁗, H-6⁗, H-6′, H-7′); 7.17÷7.11 (m, 2H, H-6, H-7); 7.05÷6.98 (m, 3H, H-4⁗, H-5′, H-5); 6.49÷6.47 (m, 2H, H-3‴, H-5‴); 5.14 (s, 2H, CH_2-C-4″); 4.73 (t, *J* = 4.75 Hz, 2H, -CH_2-CH_2-O-); 4.47 (t, *J* = 5 Hz, 2H, -CH_2-CH_2-O-); 4.00 (t, *J* = 6.5 Hz, 2H, -O-CH_2-CH_2-CH_3); 1.74 (m, 2H, -O-CH_2-CH_2-CH_3); 1.00 (t, *J* = 7.5 Hz, 3H, -O-CH_2-CH_2- CH_3). ^{13}C NMR (DMSO-d_6, 125 MHz), δ(ppm): 192.1 (C=O); 168.8 (C-2); 165.6 (C-2‴); 164.5 (C-4‴); 159.2 (C-3⁗); 151.3 (C-3′); 145.9 (C-2′); 144.8 (C-7′a); 144.5 (C-β); 142.9 (C-4″); 138.4 (C-7a); 135.9 (C-1⁗); 132.9 (C-6‴); 132.2 (C-6′); 130.1 (C-5⁗); 128.0 (C-4′); 125.9 (C-6); 123.9 (C-5″); 122.9 (C-4); 122.1 (C-3a, C-6⁗); 121.8 (C-5′); 121.5 (C-α); 121.2 (C-5); 117.4 (C-4⁗); 116.6 (C-3′a); 114.4 (C-1‴); 114.2 (C-2⁗); 111.7 (C-7′); 108.4 (C-7); 107.6 (C-5‴); 101.8 (C-3‴); 97.8 (C-3); 69.3 (-O-CH_2-CH_2-CH_3); 66.6 (-CH_2-CH_2-O-); 48.9 (-CH_2-CH_2-O-); 34.6 (CH_2-C-4″); 22.1 (-O-CH_2-CH_2-CH_3); 10.5 (-O-CH_2-CH_2-CH_3). HR-MS(ESI): caculated for $C_{39}H_{35}N_6O_6[M+H]^+$: 683.2618, found: 683.2602.

(2′*Z*, 3′*E*)-1-((1-(2-((*E*)-2‴-hydroxy-4⁗-propoxychalcon-4‴-oxy)ethyl)-1*H*-1,2,3-triazol-4-yl)methyl)indirubin-3′-oxime (6l): Yield 61 %, red solid, m.p. 185÷186 °C. ^{1}H NMR (DMSO-d_6, 500 MHz), δ(ppm): 13.59 (s broad, 1H, OH_{oxime}); 13.48 (s, 1H, $OH_{chalcone}$); 11.74 (s, 1H, H-1′); 8.68 (d, *J* = 7.5 Hz, 1H, H-4); 8.23÷8.18 (m, 2H, H-6‴, H-4′); 8.10 (s, 1H, H-5″); 7.85 (d, *J* = 9 Hz, 2H, H-2⁗, H-6⁗); 7.82 (d, *J* = 15.5 Hz, 1H, H-α); 7.78 (d, *J* = 15.5 Hz, 1H, H-β); 7.42÷7.38 (m, 2H, H-6′, H-7′); 7.17÷7.11 (m, 2H, H-6, H-7); 7.05÷6.98 (m, 4H, H-3⁗, H-5⁗, H-5′, H-5); 6.48÷6.46 (m, 2H, H-3‴, H-5‴); 5.14 (s, 2H, CH_2-C-4″); 4.73 (t, *J* = 5 Hz, 2H, -CH_2-CH_2-O-); 4.46 (t, *J* = 5 Hz, 2H, -CH_2-CH_2-

O-); 4.00 (t, *J* = 6.5 Hz, 2H, -O-C$\underline{H}_2$-CH$_2$-CH$_3$); 1.74 (m, 2H, -O-CH$_2$-C$\underline{H}_2$-CH$_3$); 0.98 (t, *J* = 7.25 Hz, 3H, -O-CH$_2$-CH$_2$- C$\underline{H}_3$). ^{13}C NMR (DMSO-d_6, 125 MHz), δ(ppm): 192 (C=O); 168.8 (C-2); 165.6 (C-2‴); 164.3 (C-4‴); 161.2 (C-4⁗); 151.4 (C-3′); 145.9 (C-2′); 144.8 (C-7′a); 144.6 (C-β); 142.9 (C-4″); 138.4 (C-7a); 132.7 (C-6‴); 132.2 (C-6′); 131.2 (C-2⁗, C-6⁗); 128.0 (C-4′); 127.1 (C-1⁗); 125.9 (C-6); 124.0 (C-5″); 122.9 (C-4); 122.1 (C-3a); 121.8 (C-5′); 121.2 (C-5); 118.4 (C-α); 116.6 (C-3′a); 115.0 (C-3⁗, C-5⁗); 114.3 (C-1‴); 111.7 (C-7′); 108.4 (C-7); 107.5 (C-5‴); 101.8 (C-3‴); 97.8 (C-3); 69.3 (-O-$\underline{C}$H$_2$-CH$_2$-CH$_3$); 66.6 (-CH$_2$-$\underline{C}$H$_2$-O-); 48.9 (-$\underline{C}$H$_2$-CH$_2$-O-); 34.6 ($\underline{C}$H$_2$-C-4″); 22.0 (-O-CH$_2$-$\underline{C}$H$_2$-CH$_3$); 10.4 (-O-CH$_2$-CH$_2$-$\underline{C}$H$_3$). HR-MS(ESI): caculated for $C_{39}H_{35}N_6O_6[M+H]^+$: 683.2618, found: 683.2607.

Synthesis of indirubin-3′-oxime derivatives (6m-p): Solution of corresponding compounds **5m-p** (0.6 mmol), sodium azide (0.63 mmol, 41 mg) in 5 mL DMSO was stirred at a temperature of 60°C in 12 hours. At the end of reaction, potassium carbonate (1.2 mmol, 166 mg) was added to the mixture and stirred for 1 hour at room temperature. Then, the solid was removed by centrifugation for 5 minutes at 12000 rpm to yield solution **A**. Compound **4** (0.5 mmol, 158 mg) and CuI (0.025 mmol, 4.8 mg) was then added to solution **A**, followed by stirring for 24 hours at room temperature. After the reaction is completed, the mixture was diluted with 100 mL of ethyl acetate and washed 3 times x100 mL with Na_2SO_4 3%. The ethyl acetate solution was then dried with anhydrous Na_2SO_4 and had the solvent removed under reduced pressure. The obtained crude extract was then chromatographed on silicagel 60, 0.04÷0.06 mm, eluting with n-hexane/ acetone (1/1, v/v) to give pure compounds **6m-p**.

(2’*Z*,3’*E*)-1-((1-(2-dimethylaminoethyl)-1*H*-1,2,3-triazol-4-yl)methyl) indirubin-3′-oxime (6m): Yield 56 %, red solid, m.p. 259÷260 °C. ^{1}H NMR (DMSO-d_6, 500 MHz), δ(ppm): 13.54 (s broad, 1H, OH$_{oxime}$); 11.77 (s, 1H, H-1′); 8.69 (d, *J* = 8 Hz, 1H, H-4); 8.24 (d, *J* = 7.5 Hz, 1H, H-4′); 7.99 (s, 1H, H-5″); 7.42 (m, 2H, H-6′, H-7′); 7.18 (m, 1H, H-6); 7.13 (d, *J* = 7.5 Hz, 1H, H-7); 7.06÷7.00 (m, 2H, H-5′, H-5); 5.12 (s, 2H, CH$_2$-C-4″); 4.38 (t, *J* = 6.25 Hz, 2H, N$_{triazole}$-C$\underline{H}_2$-CH$_2$-); 2.62 (t, *J* = 6.25 Hz, 2H, N$_{triazole}$-CH$_2$-C$\underline{H}_2$-); 2.12 (s, 6H, 2CH$_3$). ^{13}C NMR (DMSO-d_6, 125 MHz), δ(ppm): 168.6 (C-2); 151.3 (C-3′); 145.8 (C-2′); 144.7 (C-7′a); 142.5 (C-4″); 138.3 (C-7a); 132.1 (C-6′); 127.9 (C-4′); 125.8 (C-6); 123.4 (C-5″); 122.8 (C-4); 122 (C-3a); 121.7 (C-5′); 121.1 (C-5); 116.5 (C-3′a); 111.7 (C-7′); 108.3 (C-7); 97.7 (C-3); 58.2 (N$_{triazole}$-CH$_2$-$\underline{C}$H$_2$-); 47.3 (N$_{triazole}$-$\underline{C}$H$_2$-CH$_2$-); 44.9 ($\underline{C}$H$_3$); 34.5 ($\underline{C}$H$_2$-C-4″). HR-MS(ESI): calculated for $C_{23}H_{24}N_7O_2[M+H]^+$: 430.1991, found: 430.1987.

(2’*Z*,3’*E*)-1-((1-(2-piperidinoethyl)-1*H*-1,2,3-triazol-4-yl)methyl)indirubin-3′-oxime (6n): Yield 62 %, red solid, m.p. 246÷247 °C. ^{1}H NMR (DMSO-d_6, 500 MHz), δ(ppm): 13.56 (s broad, 1H, OH$_{oxime}$); 11.78 (s, 1H, H-1′); 8.69 (d, *J* = 8 Hz, 1H, H-4); 8.24 (d, *J* = 8 Hz, 1H, H-4′); 7.96 (s, 1H, H-5″); 7.42 (m, 2H, H-6′, H-7′); 7.17 (m, 1H, H-6); 7.12 (d, *J* = 7.5 Hz, 1H, H-7); 7.06÷7.00 (m, 2H, H-5′, H-5); 5.13 (s, 2H, CH$_2$-C-4″); 4.38 (t, *J* = 6.5 Hz, 2H, N$_{triazole}$-C$\underline{H}_2$-CH$_2$-); 2.60 (t, *J* = 6.25 Hz, 2H, N$_{triazole}$-CH$_2$-C$\underline{H}_2$-); 2.29 (s broad, 4H, 2H-2‴, 2H-6‴); 1.36 (m, 4H, 2H-3‴, 2H-5‴); 1.29 (m, 2H, 2H-4‴). ^{13}C NMR (DMSO-d_6, 125 MHz), δ(ppm): 168.6 (C-2); 151.3 (C-3′); 145.8 (C-2′); 144.7 (C-7′a); 142.4 (C-4″); 138.3 (C-7a); 132.1 (C-6′); 127.9 (C-4′); 125.7 (C-6); 123.6 (C-5″); 122.8 (C-4); 122 (C-3a); 121.7 (C-5′); 121 (C-5); 116.5 (C-3′a); 111.6 (C-7′); 108.3 (C-7); 97.7 (C-3); 57.6 (N$_{triazole}$-CH$_2$-$\underline{C}$H$_2$-); 53.7 (C-2‴, C-6‴); 46.9 (N$_{triazole}$-$\underline{C}$H$_2$-CH$_2$-); 34.6

($\underline{C}H_2$-C-4″); 25.4 (C-3‴, C-5‴); 23.9 (C-4‴). HR-MS(ESI): calculated for $C_{26}H_{28}N_7O_2[M+H]^+$: 470.2304, found: 470.2297.

(2’*Z*,3’*E*)-1-((1-(2-(1*H*-imidazol-1-yl)ethyl)-1*H*-1,2,3-triazol-4-yl)methyl) indirubin-3′-oxime (6o): Yield 65 %, red solid, m.p. decomposition 296 ºC. ^{1}H NMR (DMSO-d_6, 500 MHz), δ(ppm): 13.58 (s, 1H, OH_{oxime}); 11.76 (s, 1H, H-1′); 8.69 (d, *J* = 8 Hz, 1H, H-4); 8.24 (d, *J* = 7.5 Hz, 1H, H-4′); 7.83 (s, 1H, H-5″); 7.42 (m, 2H, H-6′, H-7′); 7.38 (s, 1H, H-2‴); 7.18 (m, 1H, H-6); 7.06÷7.00 (m, 3H, H-7, H-5′, H-5); 6.9 (s, 1H, H-5‴); 6.77 (s, 1H, H-4‴); 5.1 (s, 2H, CH_2-C-4″); 4.67 (t, *J* = 6 Hz, 2H, $N_{triazole}$-CH_2-$C\underline{H}_2$-); 4.42 (t, *J* = 6 Hz, 2H, $N_{triazole}$-$C\underline{H}_2$-CH_2-). ^{13}C NMR (DMSO-d_6, 125 MHz), δ(ppm): 168.6 (C-2); 151.3 (C-3′); 145.8 (C-2′); 144.8 (C-7′a); 142.9 (C-4″); 138.2 (C-7a); 137.3 (C-2‴); 132.1 (C-6′); 128.5 (C-5‴); 127.9 (C-4′); 125.8 (C-6); 123.4 (C-5″); 122.8 (C-4); 122 (C-3a); 121.7 (C-5′); 121.1 (C-5); 119.2 (C-4‴); 116.5 (C-3′a); 111.7 (C-7′); 108.3 (C-7); 97.7 (C-3); 50.0 ($N_{triazole}$-CH_2-$\underline{C}H_2$-); 45.6 ($N_{triazole}$-$\underline{C}H_2$-CH_2-); 34.5 ($\underline{C}H_2$-C-4″). HR-MS(ESI): caculated for $C_{24}H_{21}N_8O_2[M+H]^+$: 453.1787, found: 453.1779.

(2’*Z*,3’*E*)-1-((1-(2-morpholinoethyl)-1*H*-1,2,3-triazol-4-yl)methyl)indirubin-3′-oxime (6p): Yield 68 %, red solid, m.p. 174÷175 ºC. ^{1}H NMR (DMSO-d_6, 500 MHz), δ(ppm): 13.57 (s broad, 1H, OH_{oxime}); 11.78 (s, 1H, H-1′); 8.69 (d, *J* = 8 Hz, 1H, H-4); 8.24 (d, *J* = 7.5 Hz, 1H, H-4′); 7.99 (s, 1H, H-5″); 7.42 (m, 2H, H-6′, H-7′); 7.17 (m, 1H, H-6); 7.11 (d, *J* = 7,5 Hz, 1H, H-7); 7.06÷7.00 (m, 2H, H-5′, H-5); 5.14 (s, 2H, CH_2-C-4″); 4.41 (t, *J* = 6.25 Hz, 2H, $N_{triazole}$-$C\underline{H}_2$-CH_2-); 3.46 (t, *J* = 5.5 Hz, 4H, 2H-3‴, 2H-5‴); 2.66 (t, *J* = 6,5 Hz, 2H, $N_{triazole}$-CH_2-$C\underline{H}_2$-); 2.33 (s broad, 4H, 2H-2‴, 2H-6‴). ^{13}C NMR (DMSO-d_6, 125 MHz), δ(ppm): 168.6 (C-2); 151.3 (C-3′); 145.8 (C-2′); 144.7 (C-7′a); 142.5 (C-4″); 138.3 (C-7a); 132.1 (C-6′); 127.9 (C-4′); 125.7 (C-6); 123.6 (C-5″); 122.8 (C-4); 122 (C-3a); 121.7 (C-5′); 121.1 (C-5); 116.5 (C-3′a); 111.7 (C-7′); 108.3 (C-7); 97.7 (C-3); 66.1 (C-3‴, C-5‴); 57.3 ($N_{triazole}$-CH_2-$\underline{C}H_2$-); 52.9 (C-2‴, C-6‴); 46.5 ($N_{triazole}$-$\underline{C}H_2$-CH_2-); 34.6 ($\underline{C}H_2$-C-4″). HR-MS(ESI): caculated for $C_{25}H_{26}N_7O_3[M+H]^+$: 472.2097, found: 472.2093.

S2. Docking using AutoDock4

Autodock Tools (MGLTools) was utilizied to prepare protein for docking simulations. To turn the protein molecule into a free receptor, water molecules were removed. The receptor molecule was added with Polar hydrogen atoms, solvation parameters and Kollman charges. Due to the nature of ligands of not being peptides, Gasteiger charge was assigned and then non-polar hydrogens were combined. The assignment of rigid roots to the ligand was carried out automatically by the software.

The position and size of the grid box were selected to ensure that the complex in which the amino acids domain involved in binding with CHIR-98014, BIO-acetoxime and indirubin-3′-oxime were contained within. The box in which the energy scoring grid was contained had 70 × 70 × 66 grid points. The spacing between grid points was 0.375 Å so that the active site residues namely , LYS85, GLU97, LEU130, LEU132, ASP133, VAL135, PRO136, GLU137, ARG141, GLU185 and ASP200 could be included.

During the docking process, Lamarckian genetic algorithm[1], in which a maximum number of conformers was set to 50 for each compound, was employed as the search protocol. Initiation of individuals was performed randomly. The population size and the maximum number of energy evaluations were 300 and 50,000,000 respectively. Other parameters including maximum number of top individuals that automatically survived, mutation rate, crossover rate and root-mean-square cluster tolerance was set to 1, 0.02, 0.8 and 2.0 Å respectively. Default parameters for selected for step sizes for translations, quaternions and torsions.

The configuration of the computer system used for docking simulations consisted of Intel®CoreTM i7-9700K CPU @ 3.60 GHz, with 32 GB DDR4 RAM. Ubuntu-Linux 14.04.6 LTS acted as the operating system for compiling and running AutoDock 4.2.6. Model outputs were subject to further analysis via PyMOL, LigPlot+, Discovery Studio Visualizer bioinformatics tool. LigPlus and PyMOL were used to approximate hydrogen bonds distances and its binding partner residues[2].

S3. X-Ray crystallography data

Table S1. Crystallographic data and details of the structure determination for compound **4**

Empirical formula	C19 H13 N3 O2	
Formula weight	315.32	
Temperature	273(2) K	
Wavelength	0.71073 Å	
Crystal system	Orthorhombic	
Space group	P b c a	
Unit cell dimensions	a = 16.5300(13) Å	α= 90°
	b = 9.3791(8) Å	β= 90°
	c = 19.7270(18) Å	γ= 90°
Volume	3058.4(5) $Å^3$	
Z	8	
Density (calculated)	1.370 Mg/m^3	
Absorption coefficient	0.092 mm^{-1}	
F(000)	1312	
Crystal size	0.138 x 0.060 x 0.035 mm^3	
Theta range for data collection	3.216 to 25.732°	
Index ranges	-20<=h<=20, -11<=k<=11, -24<=l<=24	
Reflections collected	36804	
Independent reflections	2915 [R(int) = 0.0860]	
Completeness to theta = 25.242°	99.9 %	
Absorption correction	Semi-empirical from equivalents	
Max. and min. transmission	0.7453 and 0.6984	
Refinement method	Full-matrix least-squares on F^2	
Data / restraints / parameters	2915 / 0 / 218	
Goodness-of-fit on F^2	1.081	
Final R indices [I>2sigma(I)]	R1 = 0.0446, wR2 = 0.0958	
R indices (all data)	R1 = 0.0898, wR2 = 0.1178	
Extinction coefficient	n/a	
Largest diff. peak and hole	0.154 and -0.192 e.$Å^{-3}$	

Table S2. Selected bond lengths (Å) and bond angles (°) of compound **4**

Atoms	Bond lengths (Å)	Atoms	Bond angles (°)
O(33)-C(28)	1.251(2)	N(10)-O(11)-H(11)	109.5
O(11)-N(10)	1.390(2)	C(8)-N(7)-C(6)	112.62(16)
O(11)-H(11)	0.82	C(8)-N(7)-H(7)	123.7
N(7)-C(8)	1.364(2)	C(6)-N(7)-H(7)	123.7
N(7)-C(6)	1.389(3)	C(9)-N(10)-O(11)	111.24(16)
N(7)-H(7)	0.86	C(28)-N(27)-C(26)	110.50(16)
N(10)-C(9)	1.287(2)	C(28)-N(27)-C(30)	124.28(19)

N(27)-C(28)	1.368(3)
N(27)-C(26)	1.410(3)
N(27)-C(30)	1.458(3)
C(5)-C(4)	1.388(3)
C(5)-C(6)	1.398(3)
C(5)-C(9)	1.458(3)
C(9)-C(8)	1.489(3)
C(8)-C(29)	1.373(3)
C(25)-C(24)	1.392(3)
C(25)-C(26)	1.408(3)
C(25)-C(29)	1.459(3)
C(29)-C(28)	1.461(3)
C(6)-C(1)	1.378(3)
C(26)-C(21)	1.370(3)
C(24)-C(23)	1.386(3)
C(24)-H(24)	0.93
C(4)-C(3)	1.382(3)
C(4)-H(4)	0.93
C(23)-C(22)	1.384(3)
C(23)-H(23)	0.93
C(1)-C(2)	1.381(3)
C(1)-H(1)	0.93
C(21)-C(22)	1.386(3)
C(21)-H(21)	0.93
C(22)-H(22)	0.93
C(3)-C(2)	1.381(3)
C(3)-H(3)	0.93
C(2)-H(2)	0.93
C(30)-C(31)	1.459(3)
C(30)-H(30A)	0.97
C(30)-H(30B)	0.97
C(31)-C(32)	1.164(4)
C(32)-H(32)	0.93

C(26)-N(27)-C(30)	124.6(2)
C(4)-C(5)-C(6)	119.4(2)
C(4)-C(5)-C(9)	134.22(18)
C(6)-C(5)-C(9)	106.39(17)
N(10)-C(9)-C(5)	132.34(18)
N(10)-C(9)-C(8)	120.62(18)
C(5)-C(9)-C(8)	107.02(16)
N(7)-C(8)-C(29)	122.60(17)
N(7)-C(8)-C(9)	104.96(17)
C(29)-C(8)-C(9)	132.43(17)
C(24)-C(25)-C(26)	117.50(19)
C(24)-C(25)-C(29)	135.45(19)
C(26)-C(25)-C(29)	107.05(17)
C(8)-C(29)-C(25)	134.17(17)
C(8)-C(29)-C(28)	119.50(18)
C(25)-C(29)-C(28)	106.33(17)
O(33)-C(28)-N(27)	123.66(18)
O(33)-C(28)-C(29)	128.7(2)
N(27)-C(28)-C(29)	107.61(18)
C(1)-C(6)-N(7)	128.89(19)
C(1)-C(6)-C(5)	122.1(2)
N(7)-C(6)-C(5)	109.00(18)
C(21)-C(26)-C(25)	123.63(19)
C(21)-C(26)-N(27)	127.88(19)
C(25)-C(26)-N(27)	108.49(18)
C(23)-C(24)-C(25)	119.1(2)
C(23)-C(24)-H(24)	120.4
C(25)-C(24)-H(24)	120.4
C(3)-C(4)-C(5)	118.7(2)
C(3)-C(4)-H(4)	120.7
C(5)-C(4)-H(4)	120.7
C(22)-C(23)-C(24)	121.9(2)
C(22)-C(23)-H(23)	119.1
C(24)-C(23)-H(23)	119.1
C(6)-C(1)-C(2)	117.5(2)
C(6)-C(1)-H(1)	121.3
C(2)-C(1)-H(1)	121.3
C(26)-C(21)-C(22)	117.7(2)
C(26)-C(21)-H(21)	121.1
C(22)-C(21)-H(21)	121.1
C(23)-C(22)-C(21)	120.1(2)
C(23)-C(22)-H(22)	119.9

C(21)-C(22)-H(22)	119.9
C(2)-C(3)-C(4)	120.9(2)
C(2)-C(3)-H(3)	119.5
C(4)-C(3)-H(3)	119.5
C(1)-C(2)-C(3)	121.5(2)
C(1)-C(2)-H(2)	119.3
C(3)-C(2)-H(2)	119.3
N(27)-C(30)-C(31)	113.0(2)
N(27)-C(30)-H(30A)	109
C(31)-C(30)-H(30A)	109
N(27)-C(30)-H(30B)	109
C(31)-C(30)-H(30B)	109
H(30A)-C(30)-H(30B)	107.8
C(32)-C(31)-C(30)	177.7(3)
C(31)-C(32)-H(32)	180

S4. Detailed description for docking studies

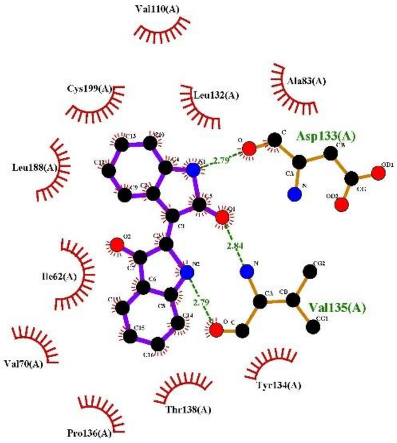

(A)

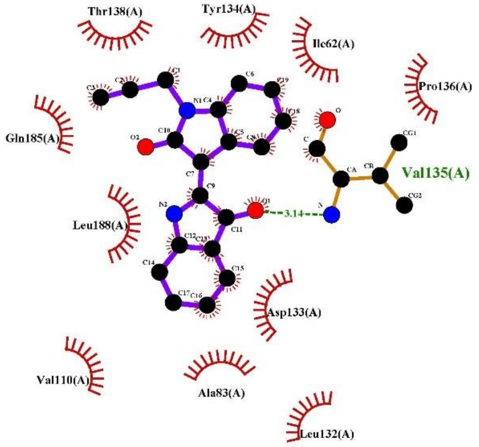

(B)

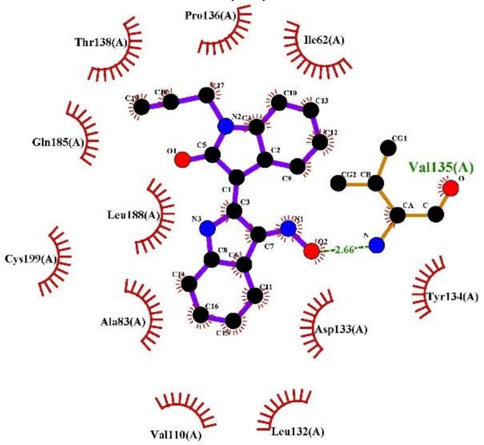

(C)

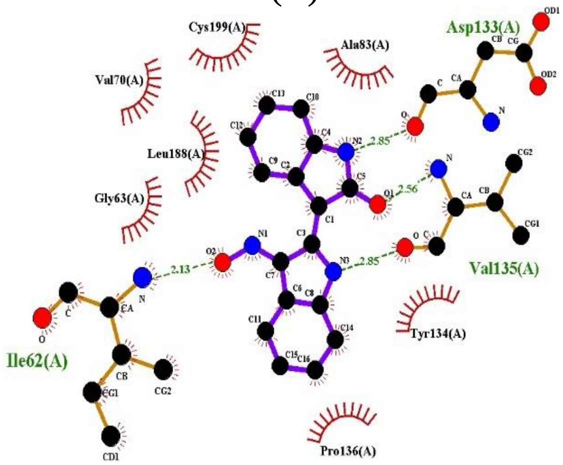

(D)

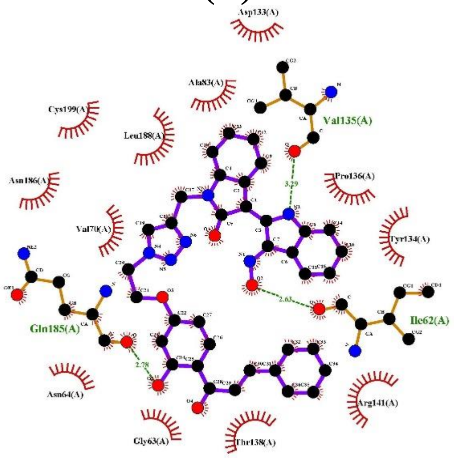

(E)

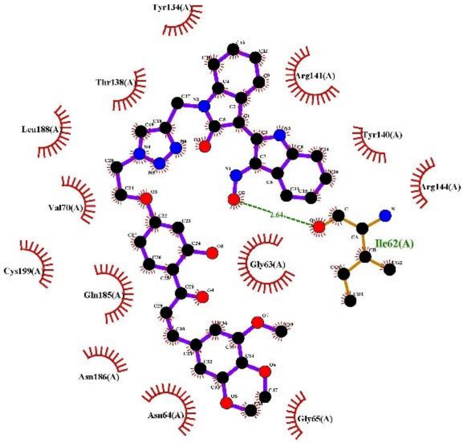

(F)

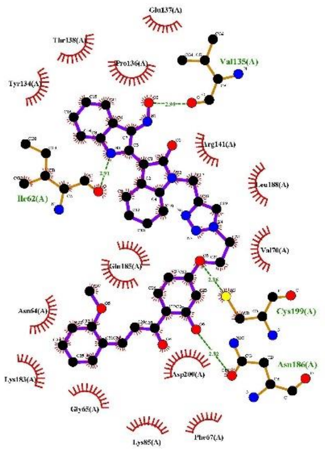

(G)

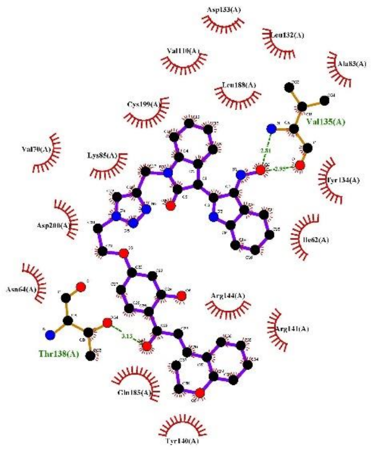

(H)

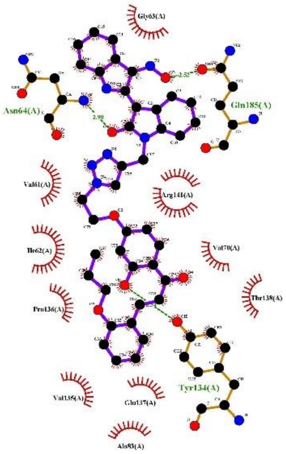

(I)

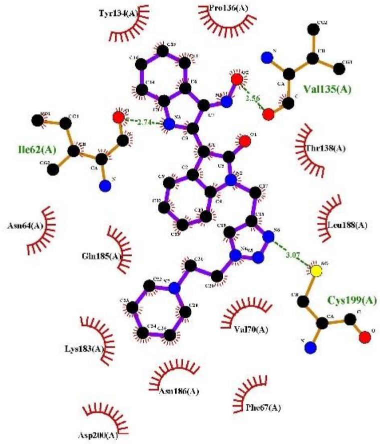

(K)

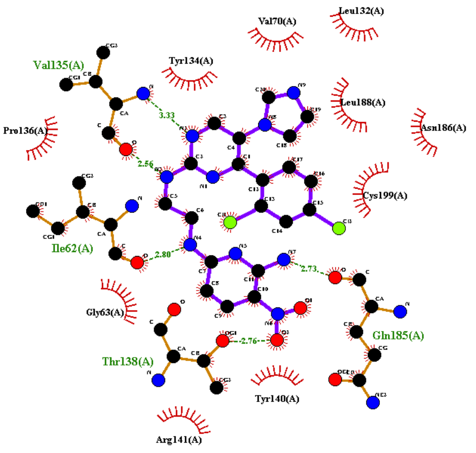

(L)

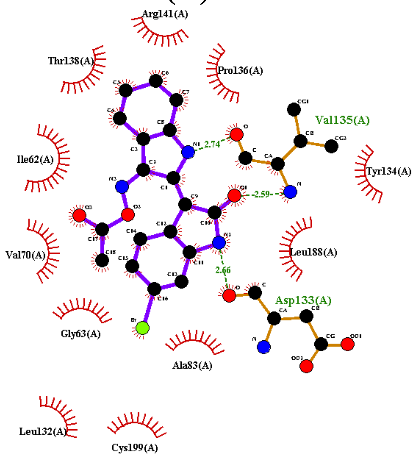

(M)

Figure S1. Hydrogen bonding patterns of compounds with GSK-3β protein (PDB ID: 1Q41). (A) Compound 1; (B) Compound 3; (C) Compound 4; (D) Indirubin-3′-oxime (Compound 2); (E) Compound 6a; (F) Compound 6b; (G) Compound 6c; (H) Compound 6f; (I) Compound 6i; (K) Compound 6n; (L) CHIR-98014; (M) BIO-acetoxime

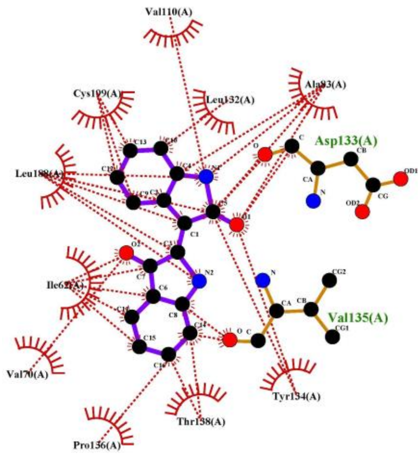
Val110(A)
Cys199(A)
Leu132(A)
Ala83(A)
Asp133(A)
Leu188(A)
Ile62(A)
Val135(A)
Val70(A)
Tyr134(A)
Thr138(A)
Pro136(A)

(A)

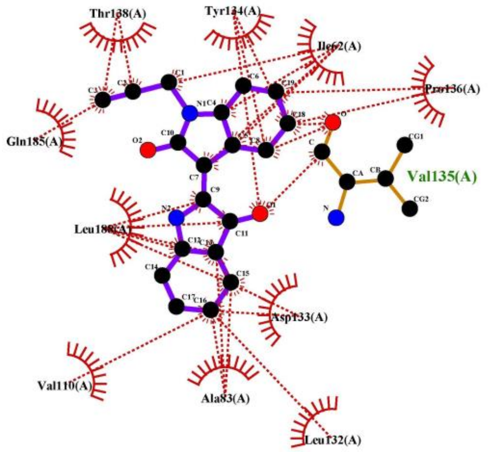
Thr138(A)
Tyr134(A)
Ile62(A)
Pro136(A)
Gln185(A)
Val135(A)
Leu188(A)
Asp133(A)
Val110(A)
Ala83(A)
Leu132(A)

(B)

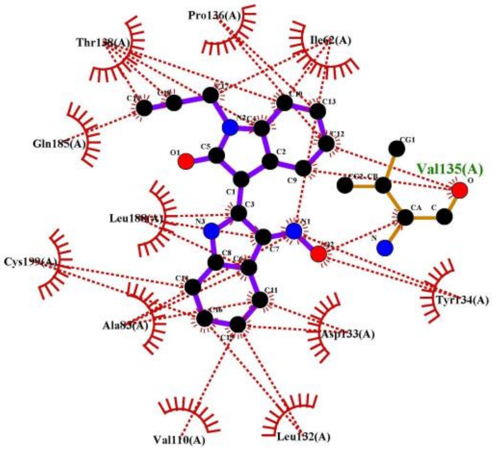
Pro136(A)
Thr138(A)
Ile62(A)
Gln185(A)
Val135(A)
Leu188(A)
Cys199(A)
Tyr134(A)
Ala83(A)
Asp133(A)
Val110(A)
Leu132(A)

(C)

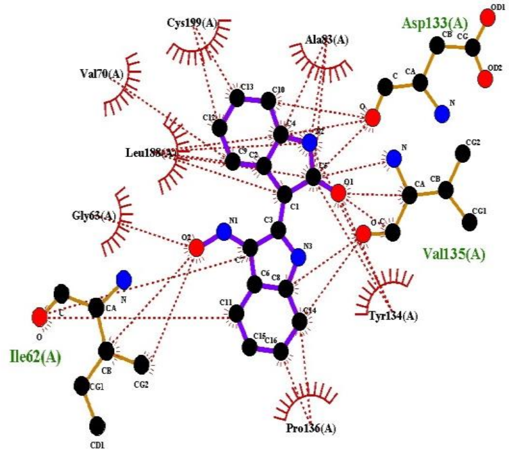
Cys199(A)
Ala83(A)
Asp133(A)
Val70(A)
Leu188(A)
Val135(A)
Gly63(A)
Tyr134(A)
Ile62(A)
Pro136(A)

(D)

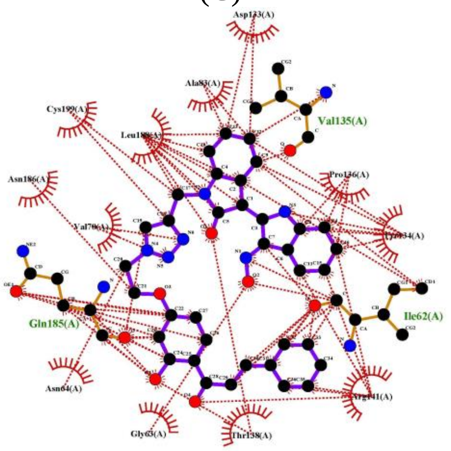
Asp133(A)
Ala83(A)
Val135(A)
Cys199(A)
Asn186(A)
Pro136(A)
Val70(A)
Gln185(A)
Ile62(A)
Asn64(A)
Gly65(A)
Thr138(A)

(E)

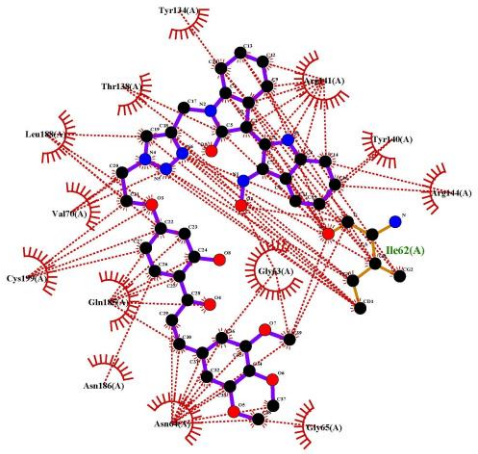
Tyr134(A)
Thr138(A)
Tyr140(A)
Val70(A)
Ile62(A)
Cys199(A)
Asn186(A)
Gly65(A)

(F)

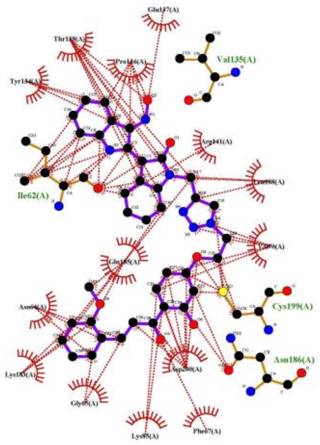

(G)

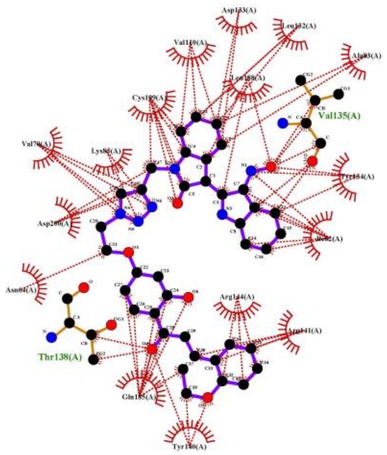

(H)

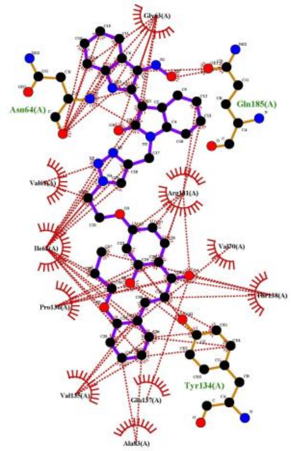

(I)

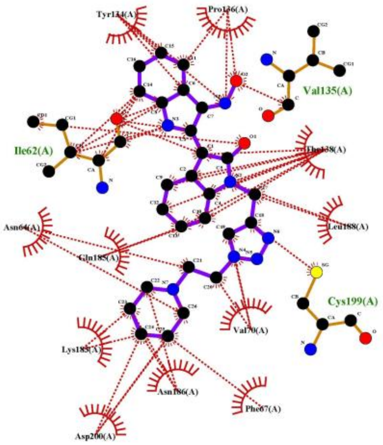

(K)

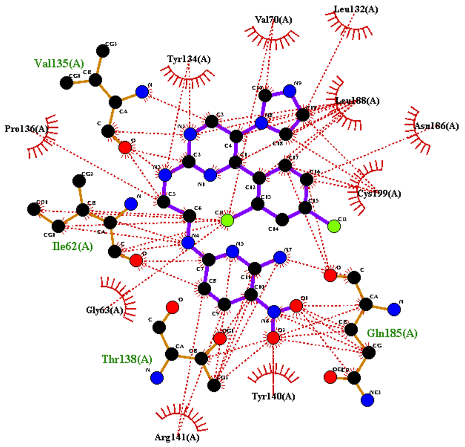

(L)

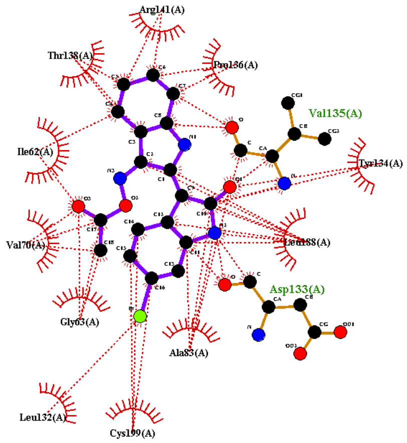

(M)

Figure S2. Hydrophobic interactions. (A) Compound 1; (B) Compound 3; (C) Compound 4; (D) Indirubin-3'-oxime (Compound 2); (E) Compound 6a; (F) Compound 6b; (G) Compound 6c; (H) Compound 6f; (I) Compound 6i; (K) Compound 6n; (L) CHIR-98014; (M) BIO-acetoxime

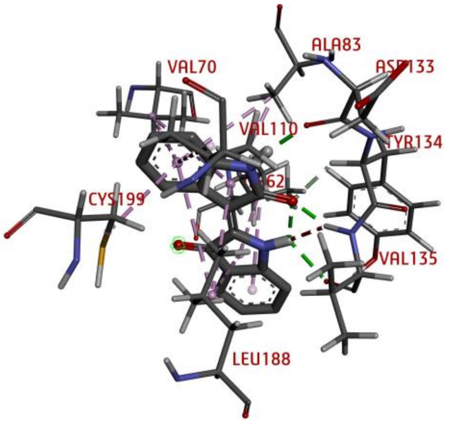
ALA83
ASP133
VAL70
VAL110
TYR134
CYS199
VAL135
LEU188

(A)

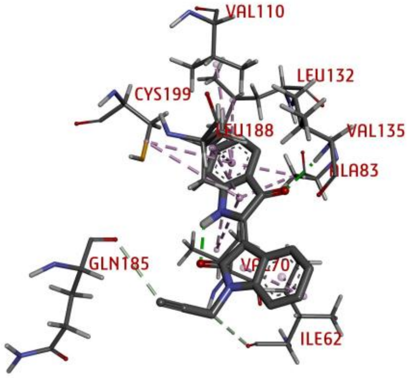
VAL110
LEU132
CYS199
LEU188
VAL135
ALA83
GLN185
VAL70
ILE62

(B)

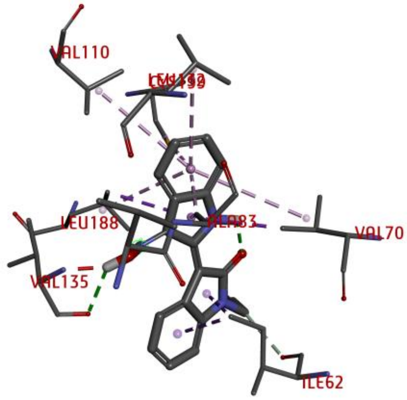
VAL110
LEU188
ALA83
VAL70
VAL135
ILE62

(C)

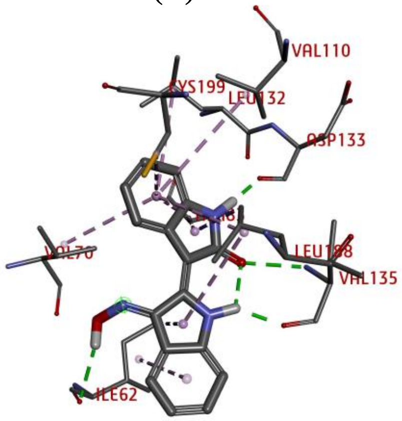
VAL110
CYS199
LEU132
ASP133
LEU188
VAL135
ILE62

(D)

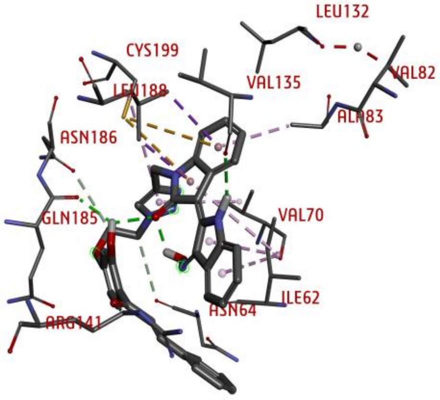
LEU132
CYS199
VAL82
VAL135
LEU188
ALA83
ASN186
GLN185
VAL70
ILE62
ASN64
ARG141

(E)

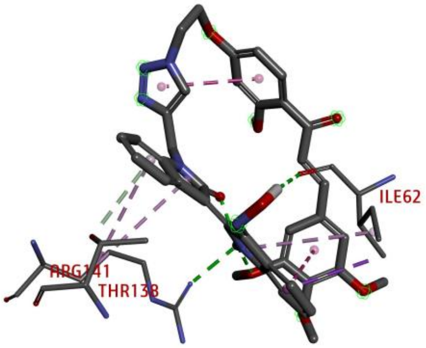
ILE62
ARG141
THR138

(F)

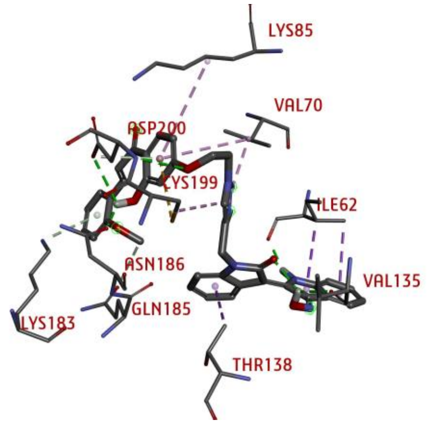

(G)

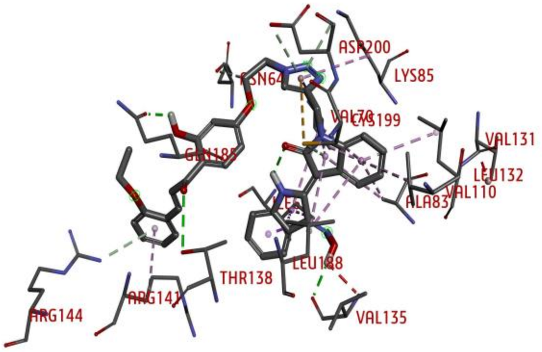

(H)

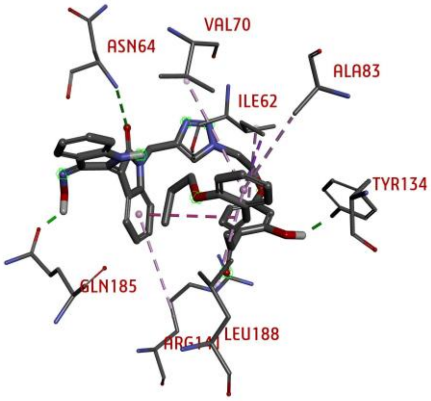

(I)

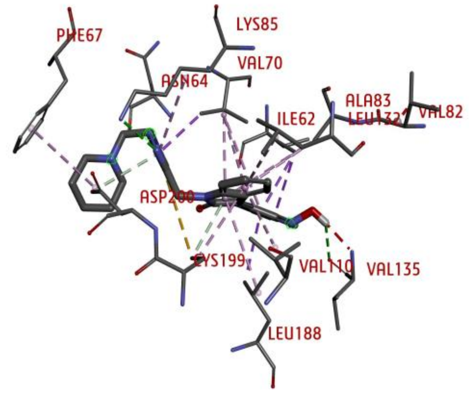

(K)

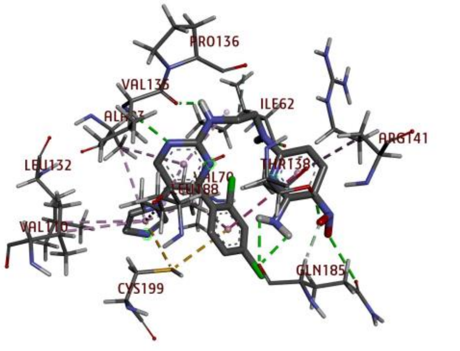

(L)

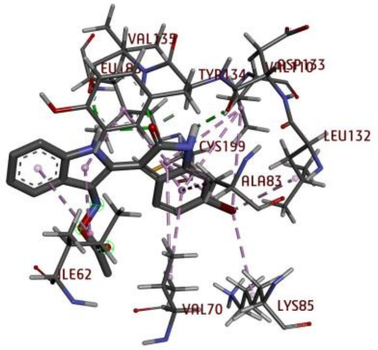

(M)

Figure S3. Stereoview of the binding mode of designed compounds in the ATP-binding site of GSK-3β, suggested by molecular docking studies. Molecules and important interacting residues are represented in stick and line model, H-bonding interactions are presented with green lines. (A) Compound 1; (B) Compound 3; (C) Compound 4; (D) Indirubin-3′-oxime (Compound 2); (E) Compound 6a; (F) Compound 6b; (G) Compound 6c; (H) Compound 6f; (I) Compound 6i; (K) Compound 6n; (L) CHIR-98014; (M) BIO-acetoxime

S5. FPL Simulations

Table S3. The mean rupture force F_{max} and pulling work W was evaluated over 8 independent SMD trajectories in various FFs. The experimental affinity ΔG_{exp} was estimated from the IC50, which was approximated that IC50 ~ K_i. The error was the standard error of the mean.

N^0	Ligand	ΔG_{EXP}	ΔE_{cou}	ΔE_{vdW}	ΔE
1	1	-6.45	-12.8 ± 0.3	-34.0 ± 0.3	-46.8 ± 0.3
2	2	-6.58	-14.0 ± 0.3	-36.3 ± 0.2	-50.3 ± 0.3
3	3	-6.45	-5.4 ± 0.4	-39.5 ± 0.3	-44.9 ± 0.4
4	4	-6.45	-9.2 ± 0.3	-36.1 ± 0.4	-45.3 ± 0.4
5	6a	-7.48	-9.3 ± 0.4	-53.8 ± 0.6	-63.1 ± 0.7
6	6b	-6.45	-10.5 ± 0.5	-45.1 ± 0.5	-55.6 ± 0.6
7	6c	-7.51	-24.7 ± 0.9	-51.2 ± 0.5	-75.9 ± 0.8
8	6f	-7.82	-5.2 ± 0.3	-62.3 ± 0.6	-67.5 ± 0.7
9	6i	-7.74	-20.3 ± 0.4	-45.4 ± 0.3	-65.7 ± 0.4
10	6n	-6.77	-2.4 ± 0.2	-43.4 ± 0.5	-45.8 ± 0.5

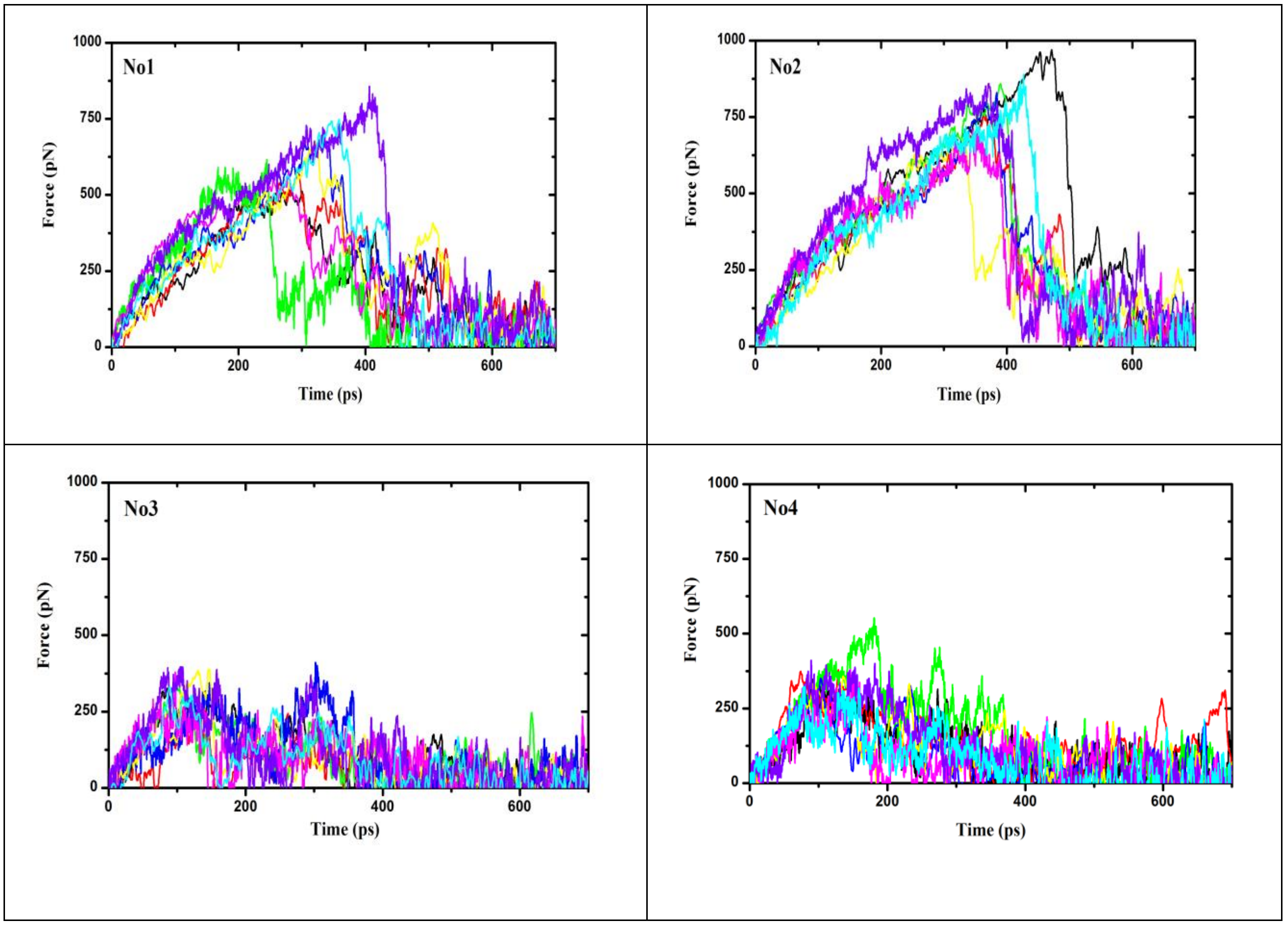

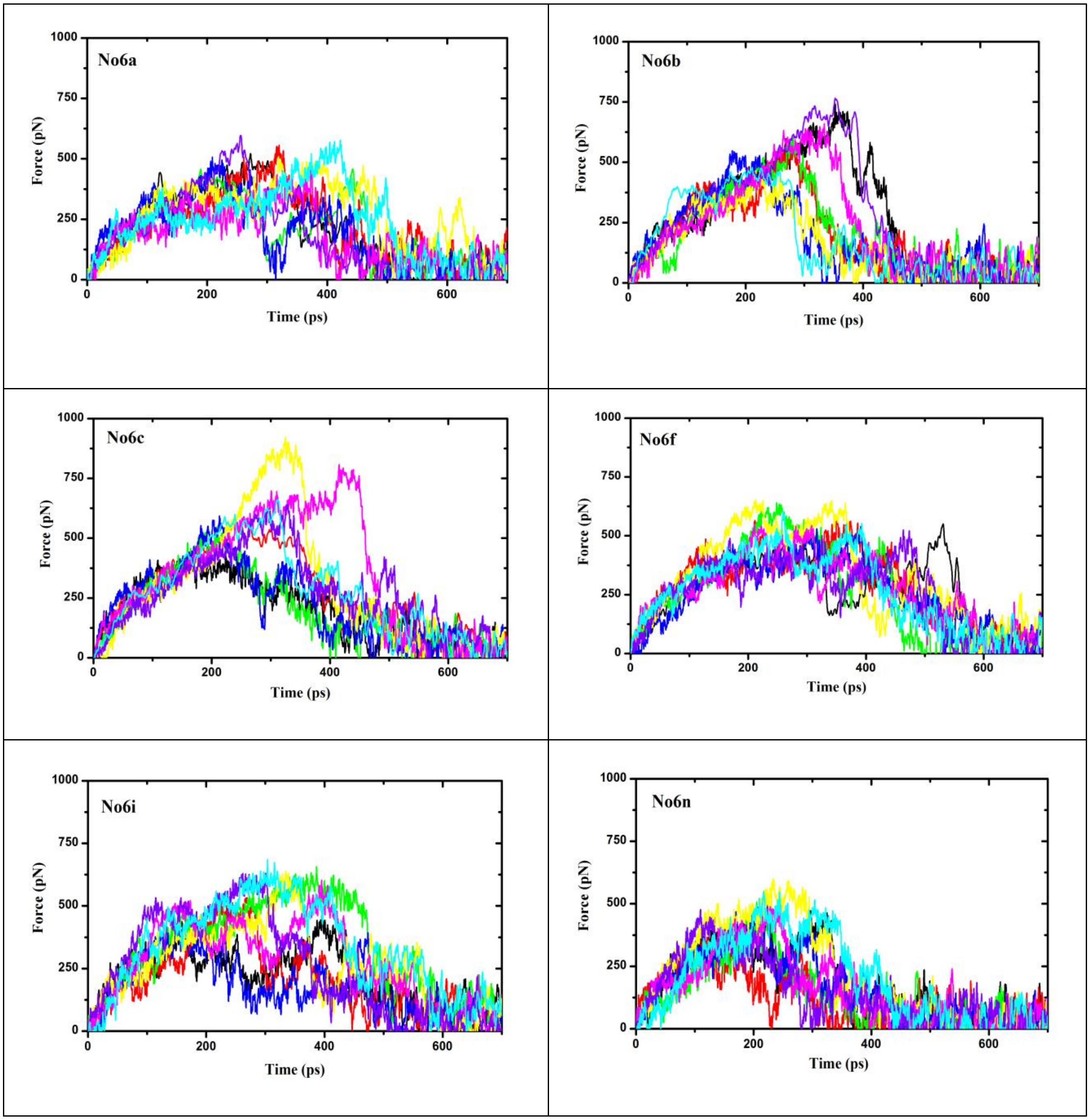

Figure S4. The mean force of 10 complexes over 8 independent SMD trajectories. The data was monitored every 0.1 ps

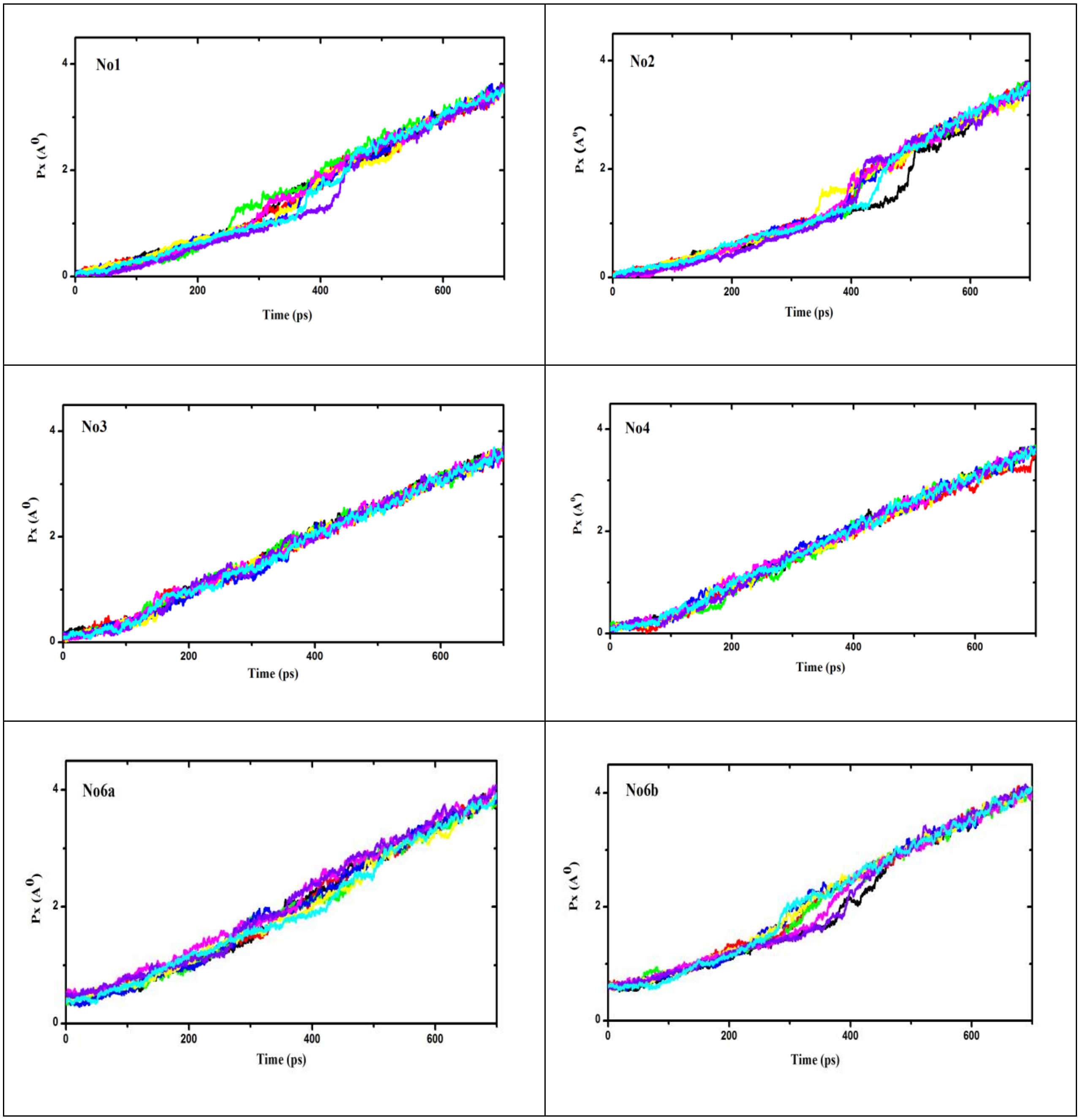
No1
No2
No3
No4
No6a
No6b
Px (A0)
Time (ps)
0
2
4
0
200
400
600

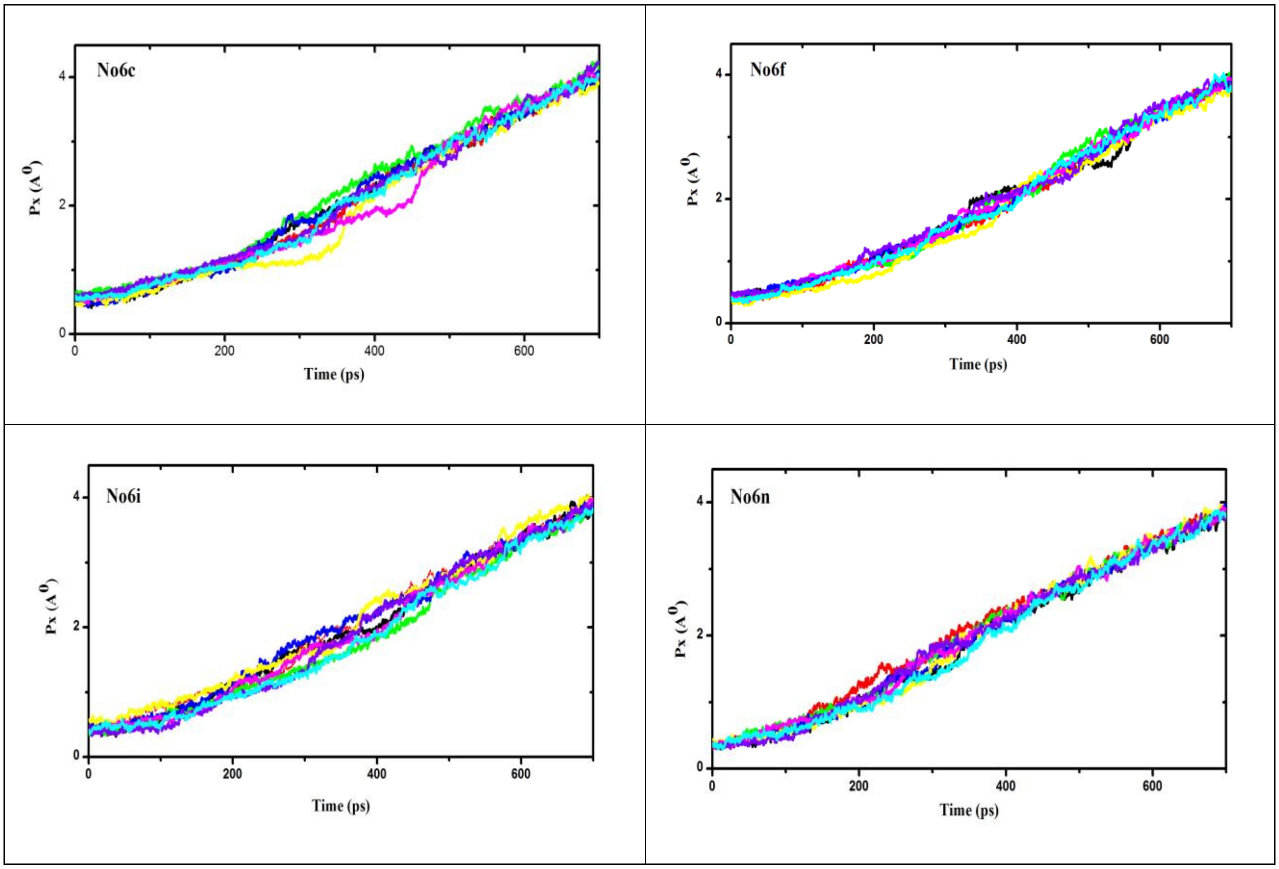

Figure S5. The mean displacement of 10 complexes during 8 independent SMD trajectories

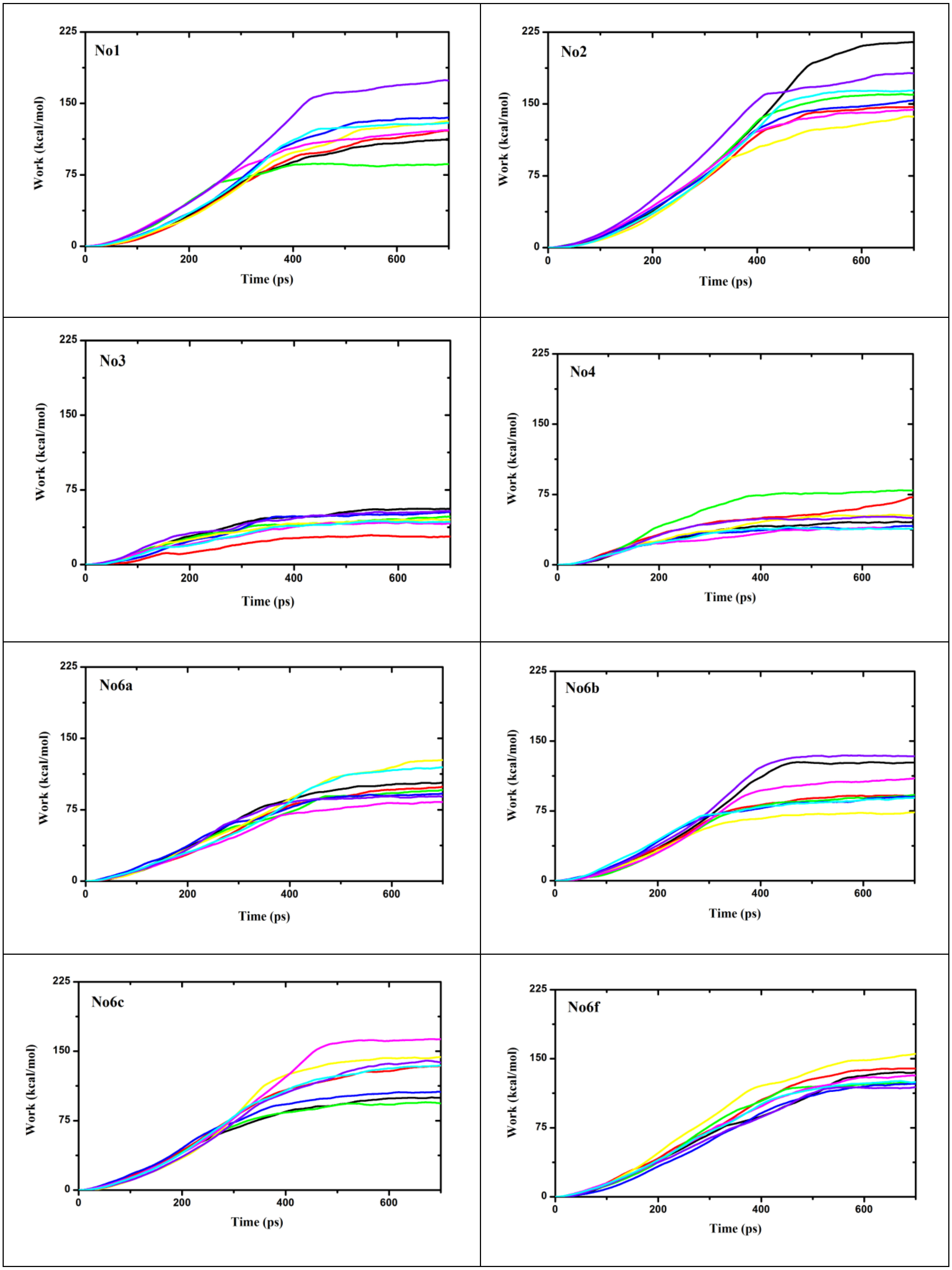
No1
No2
No3
No4
No6a
No6b
No6c
No6f
Work (kcal/mol)
Time (ps)
0
75
150
225
0
200
400
600

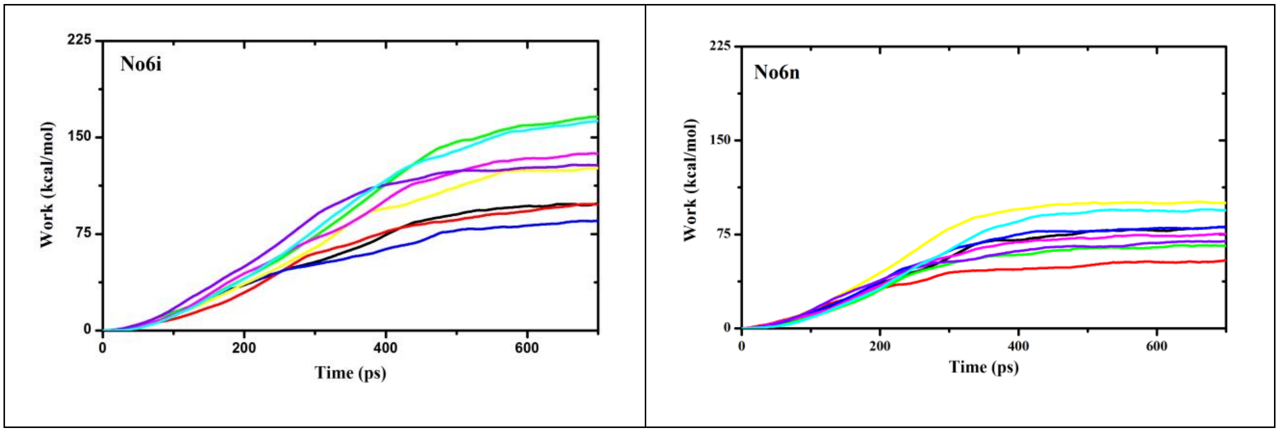

Figure S6. The mean work of 10 complexes during 8 independent SMD trajectories.

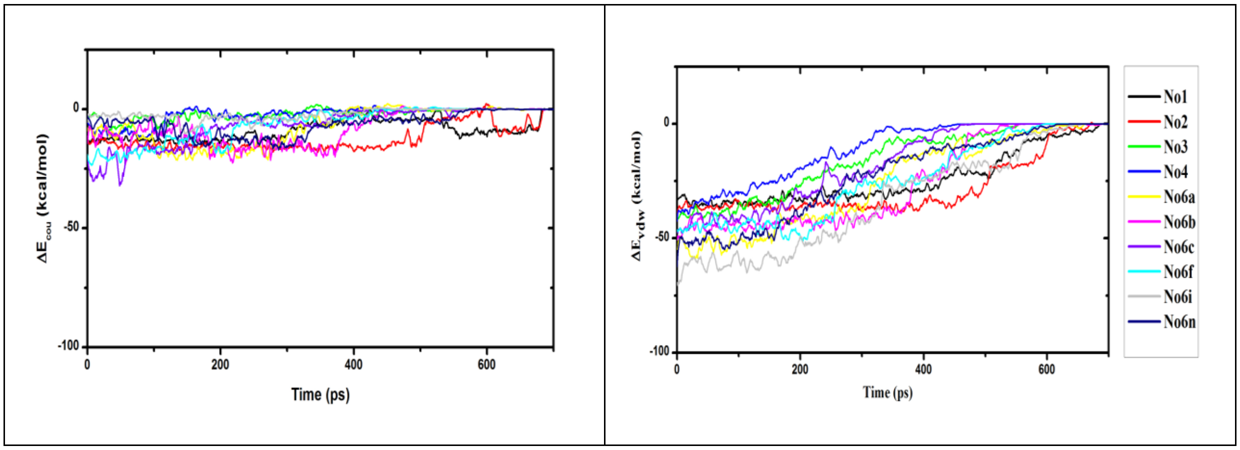

Figure S7. The means of electrostatics ΔE_{cou} (A) and vdW ΔE_{vdw} (B) total interaction energy difference between 10 complexes and protein during the SMD simulations.

S6. Spectroscopic data

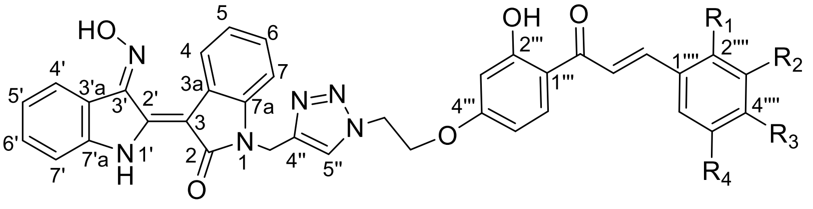

6a-l

6a: R_1=R_2=R_3=R_4=H
6b: R_1=H; R_2=R_3=R_4=OCH_3
6c: R_2=R_3=R_4=H; R_1=OCH_3
6d: R_1=R_3=R_4=H; R_2=OCH_3
6e: R_1=R_2=R_4=H; R_3=OCH_3
6f: R_2=R_3=R_4=H; R_1=OC_2H_5
6g: R_1=R_3=R_4=H; R_2=OC_2H_5
6h: R_1=R_2=R_4=H; R_3=OC_2H_5
6i: R_2=R_3=R_4=H; R_1=n-OC_3H_7
6k: R_1=R_3=R_4=H; R_2=n-OC_3H_7
6l: R_1=R_2=R_4=H; R_3=n-OC_3H_7

Figure S8. General structure of derivatives **6a-l**

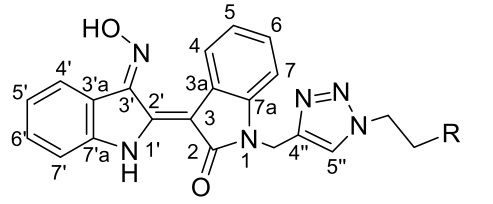

6m-p

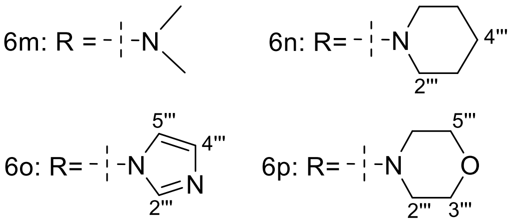

Figure S9. General structure of derivatives **6m-p**

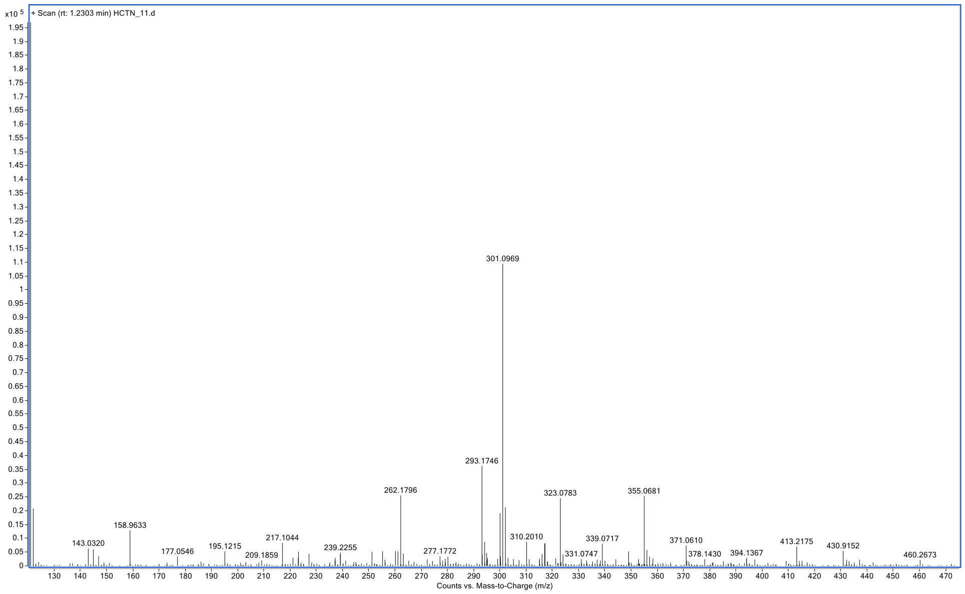

HR-MS(ESI) spectrum of compound **3**

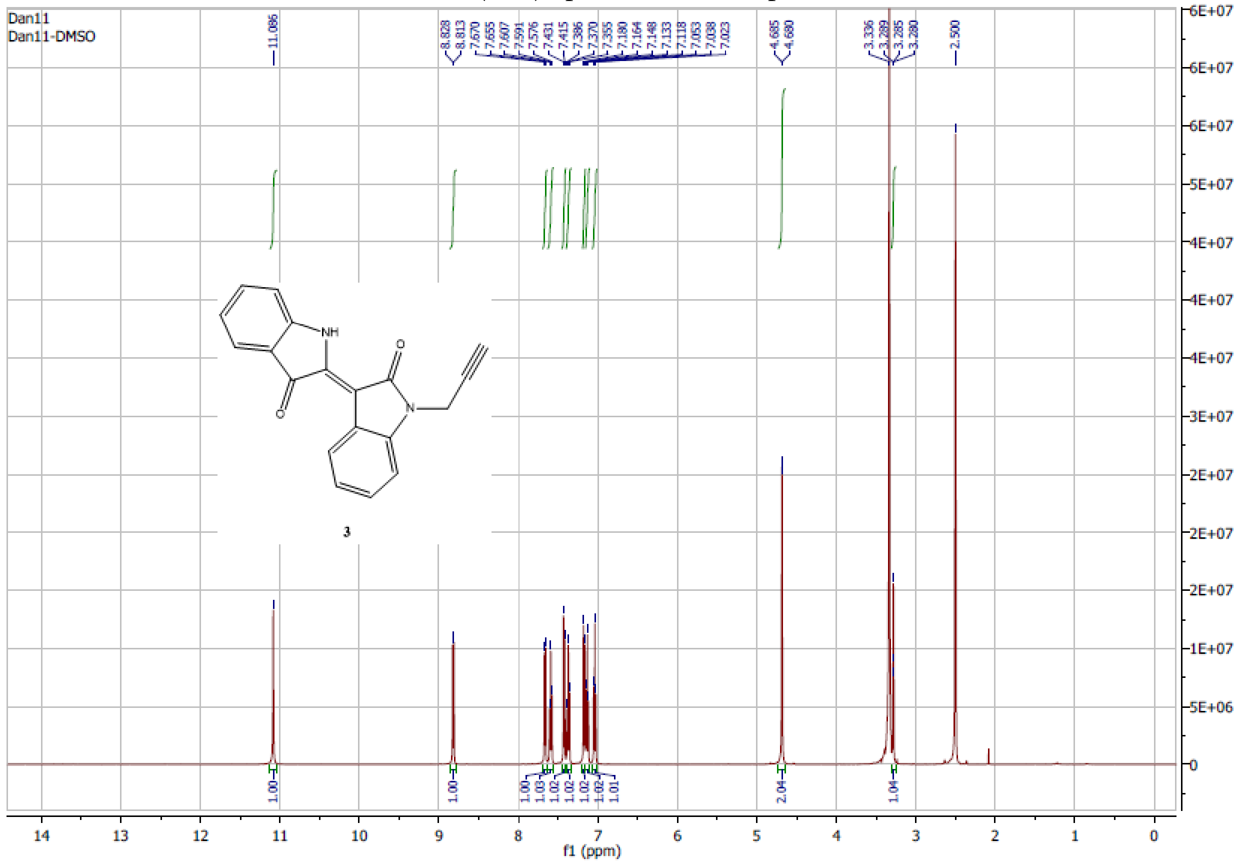

^{1}H-NMR spectrum of compound **3**

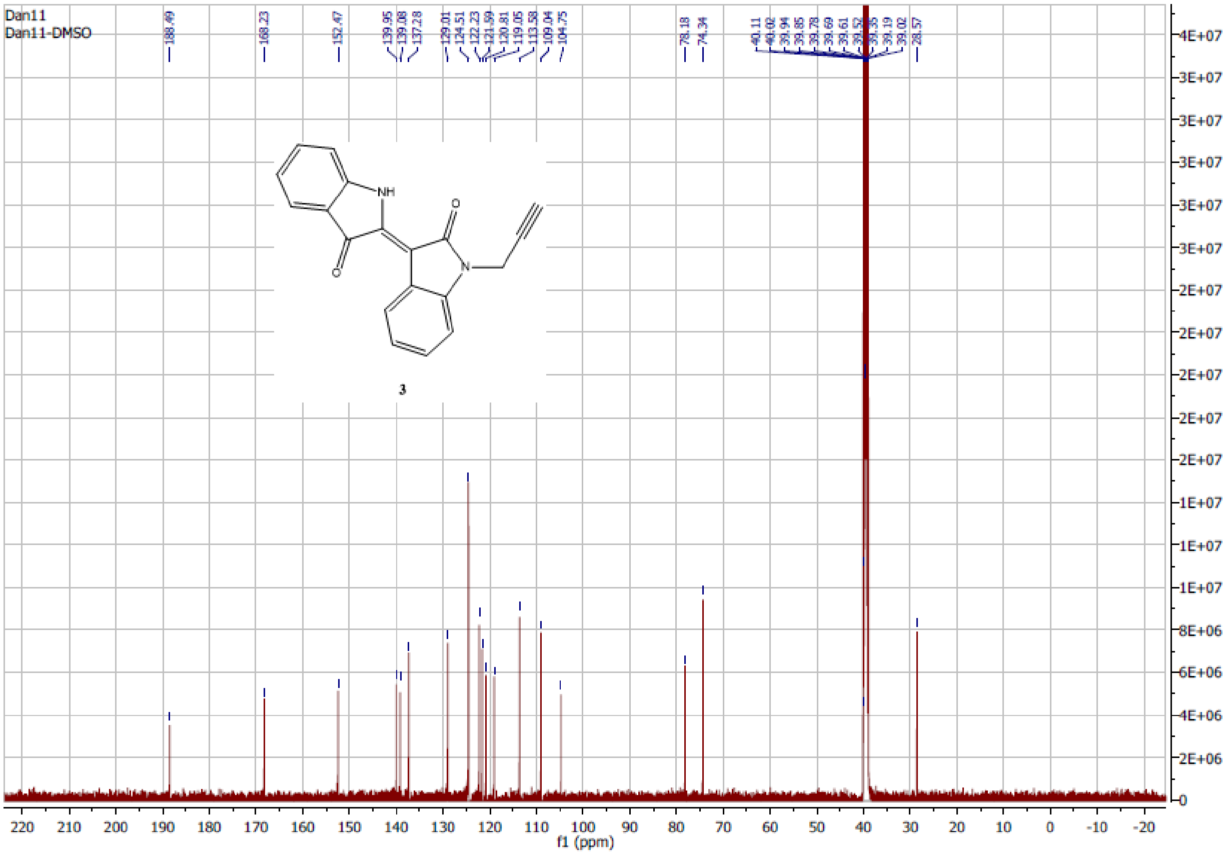

^{13}C-NMR spectrum of compound **3**

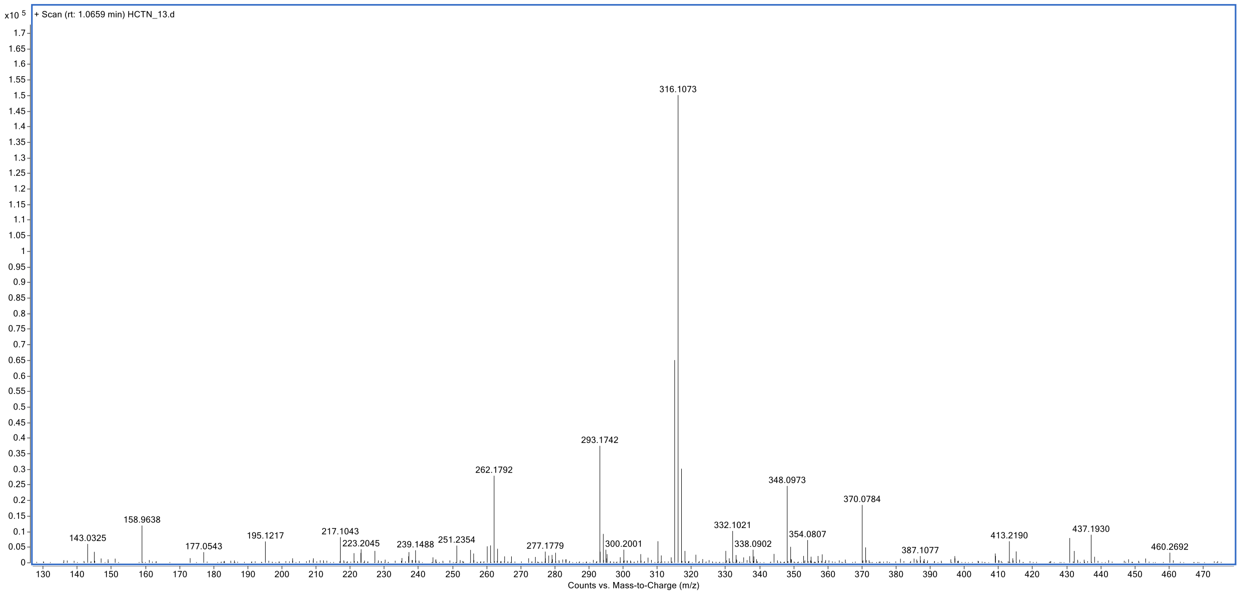

HR-MS(ESI) spectrum of compound **4**

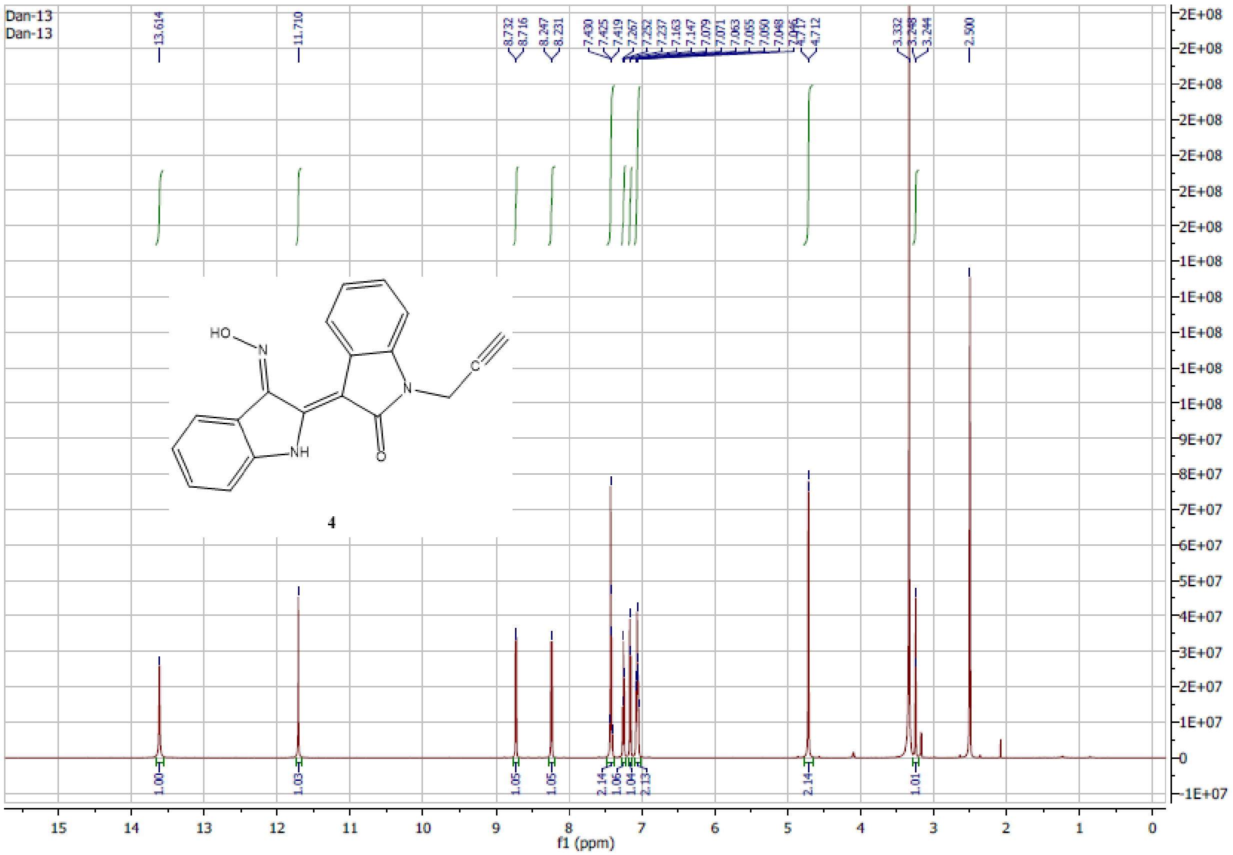

^{1}H-NMR spectrum of compound **4**

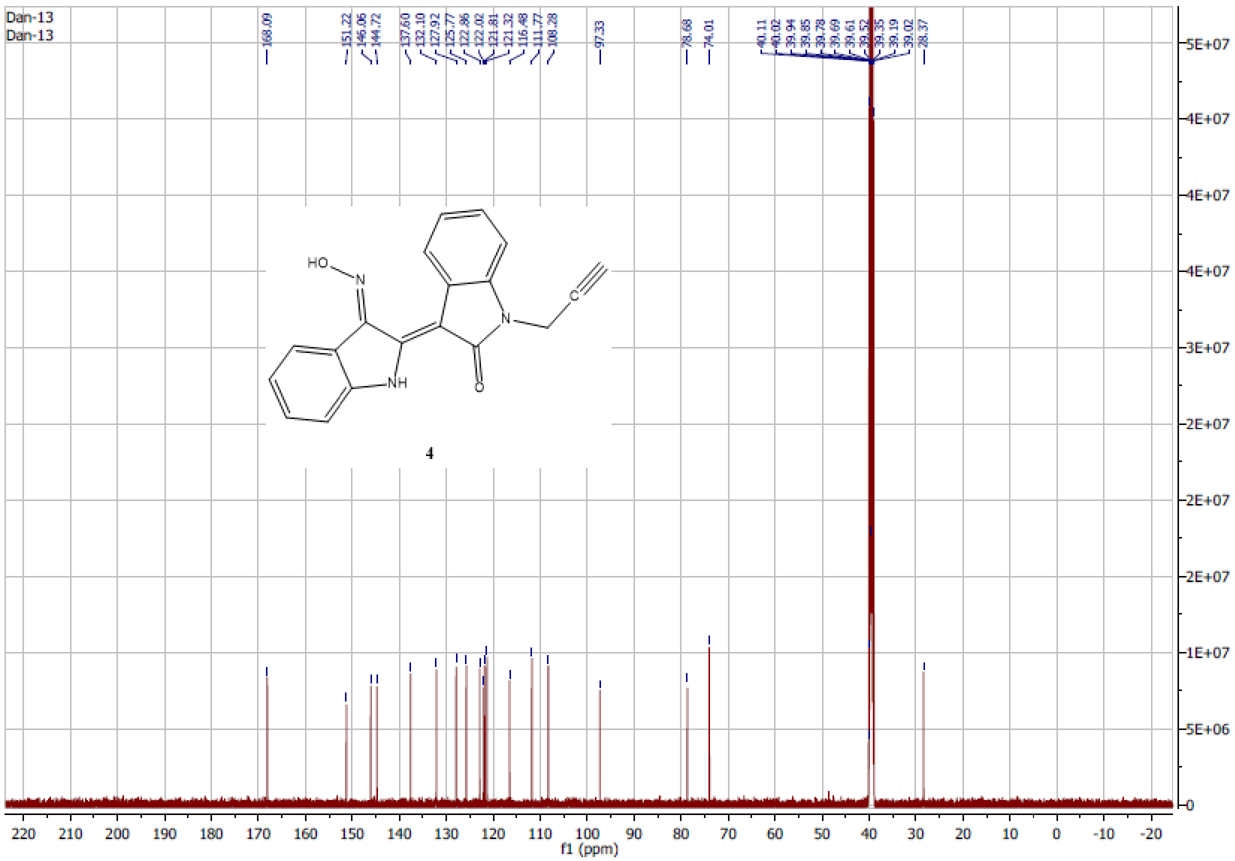

^{13}C-NMR spectrum of compound **4**

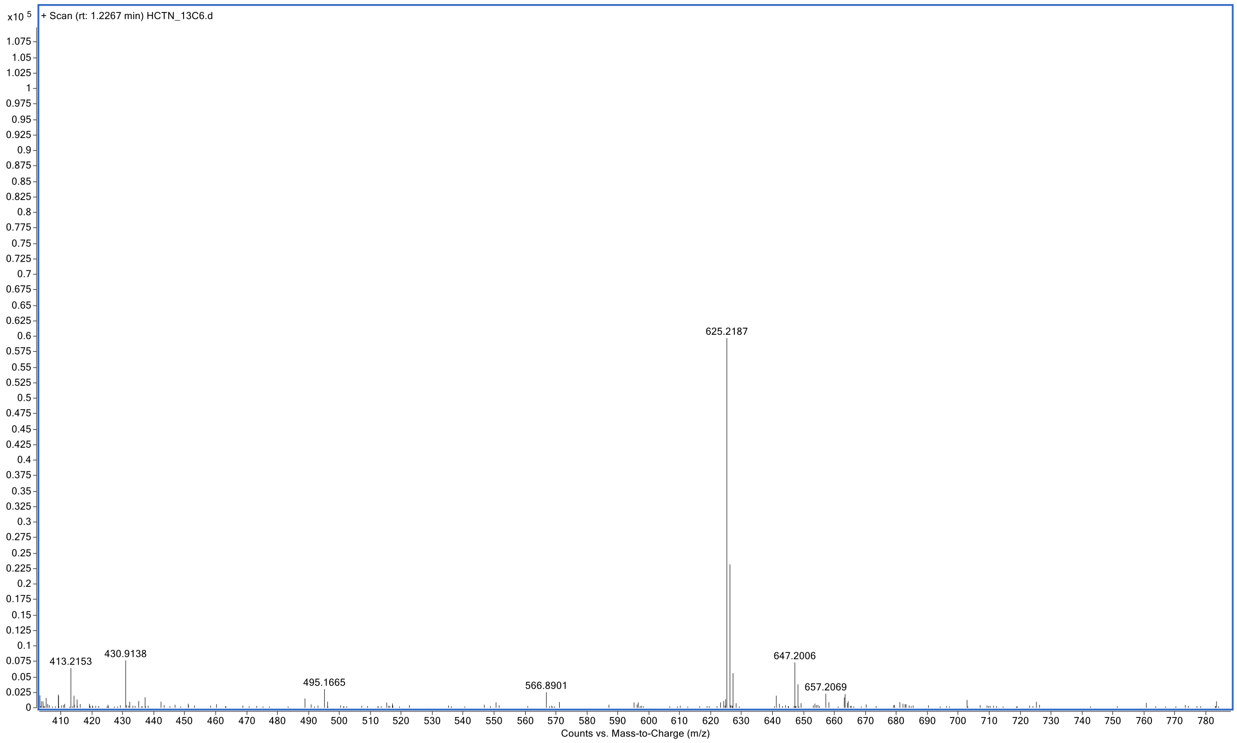

HR-MS(ESI) spectrum of compound **6a**

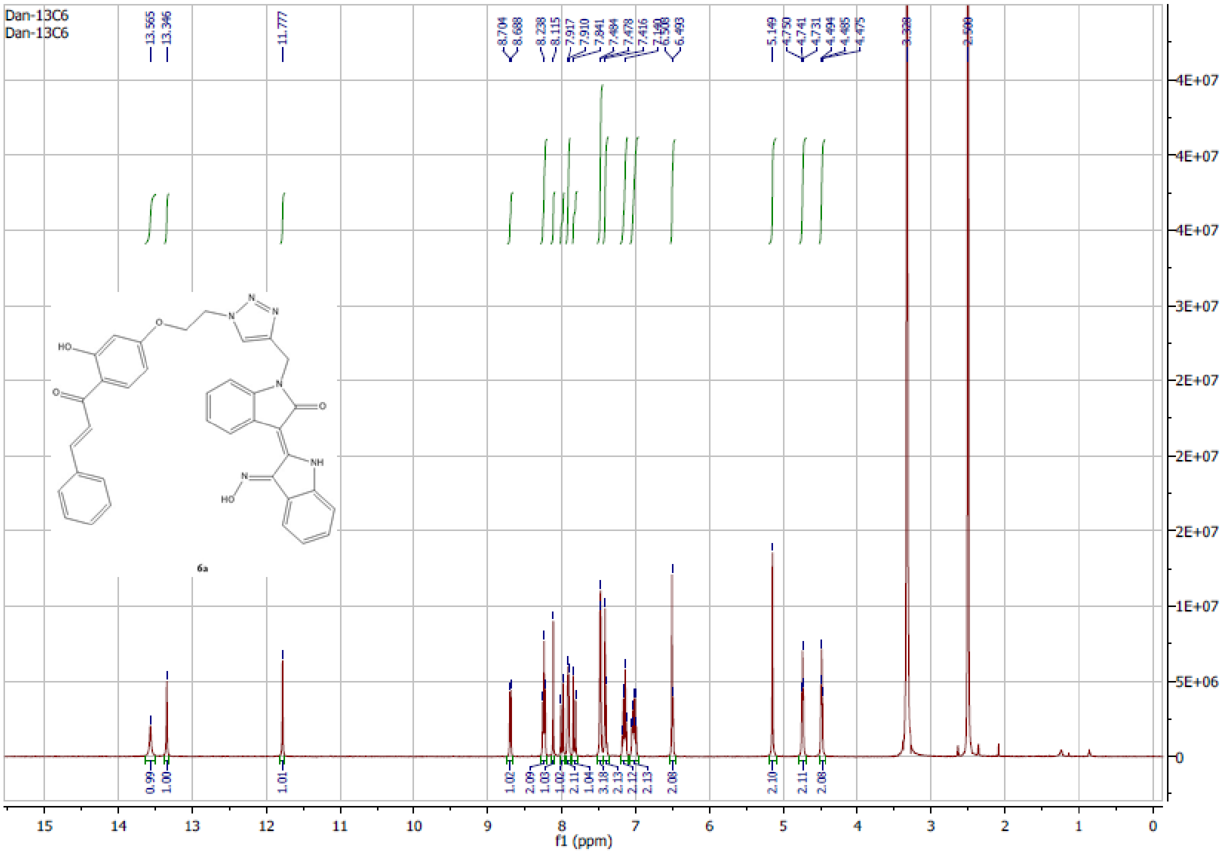

^{1}H-NMR spectrum of compound **6a**

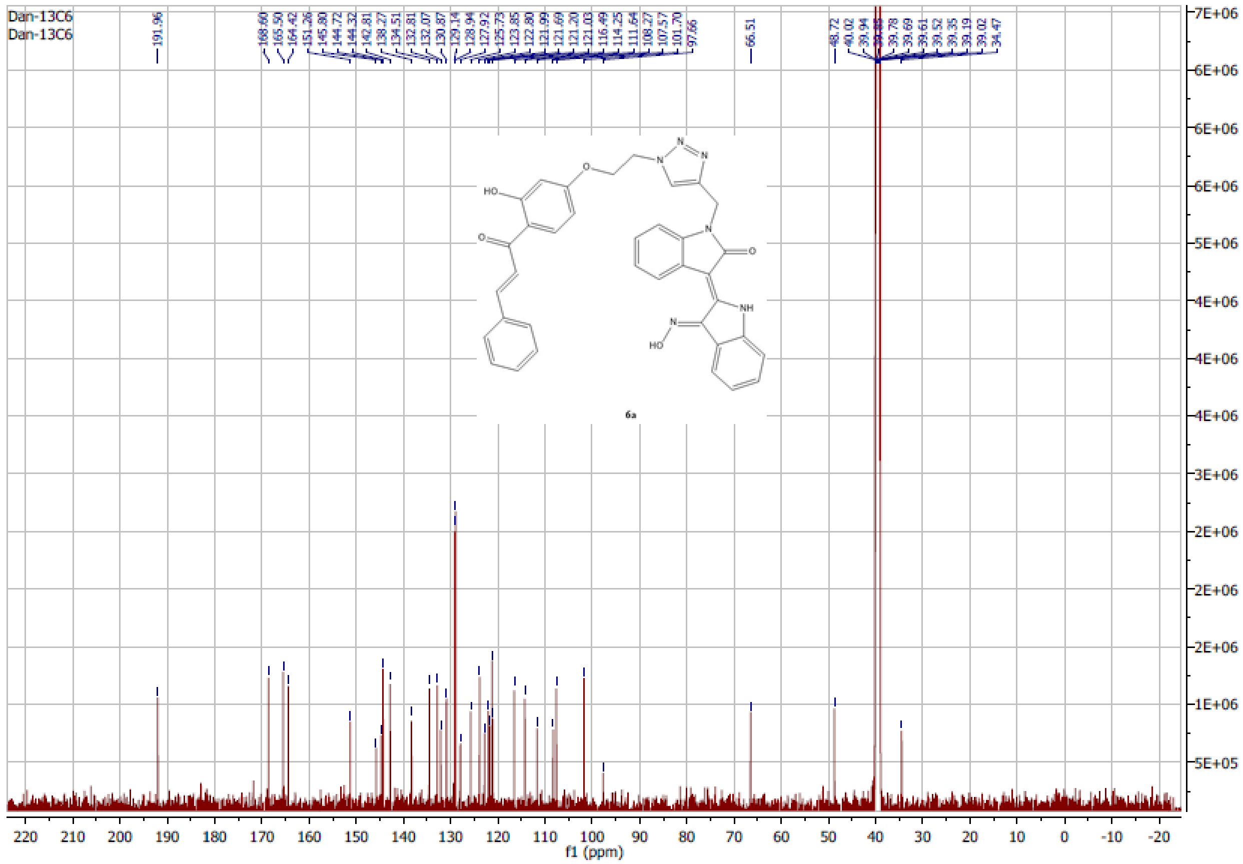

^{13}C-NMR spectrum of compound **6a**

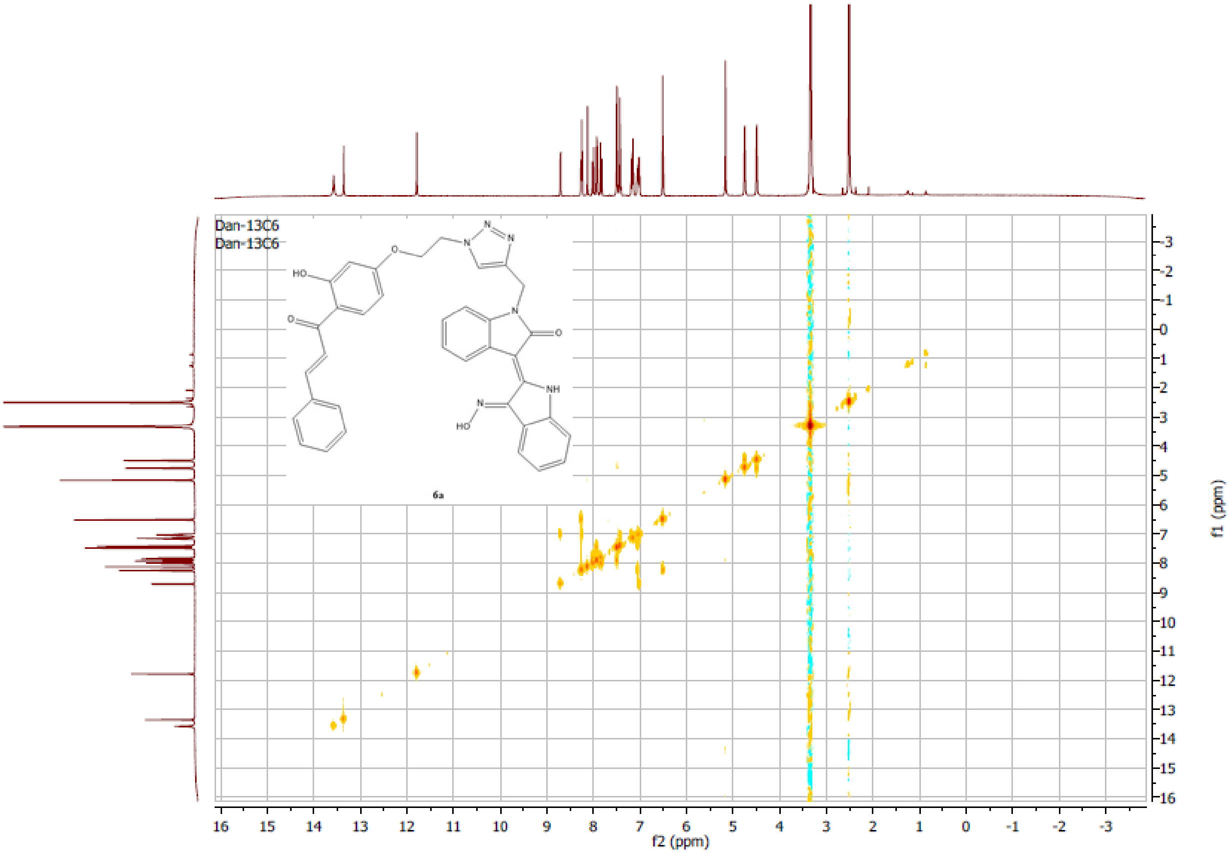

COSY spectrum of compound **6a**

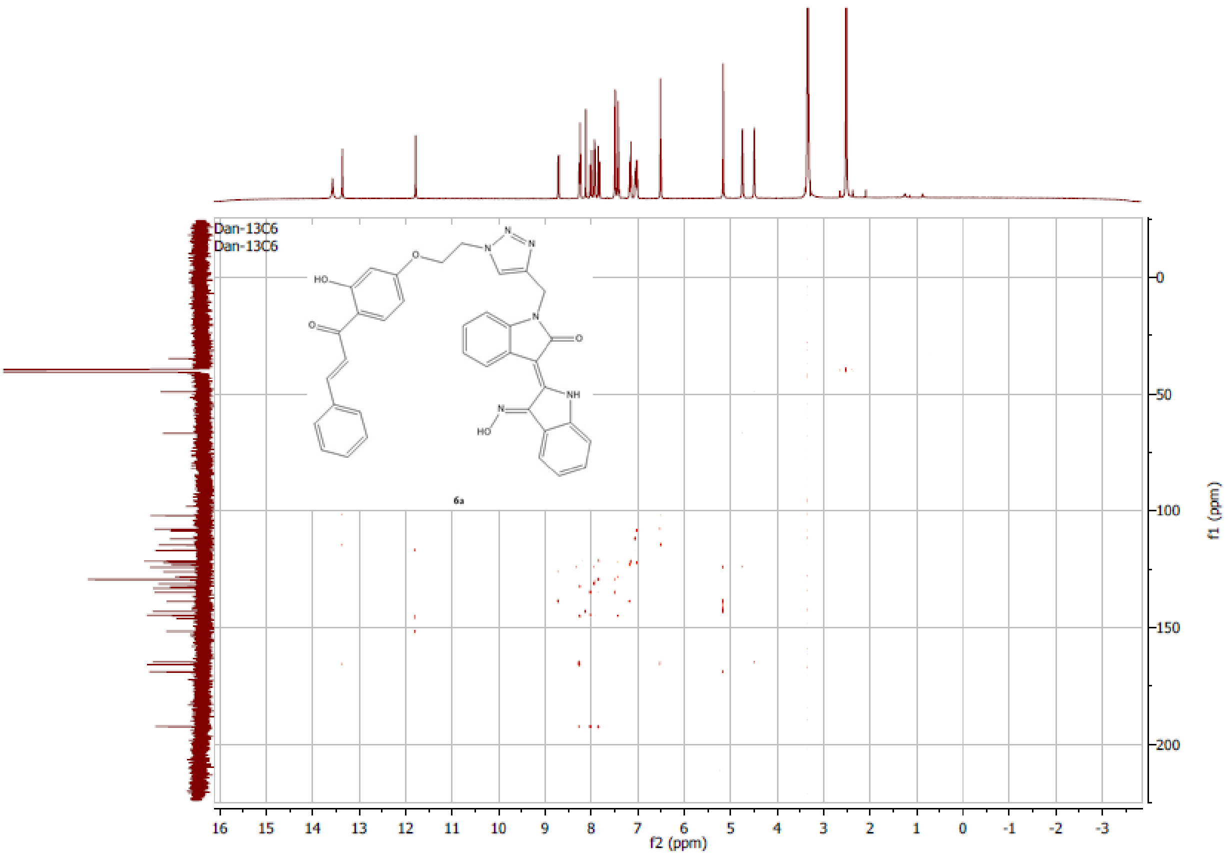

HMBC spectrum of compound **6a**

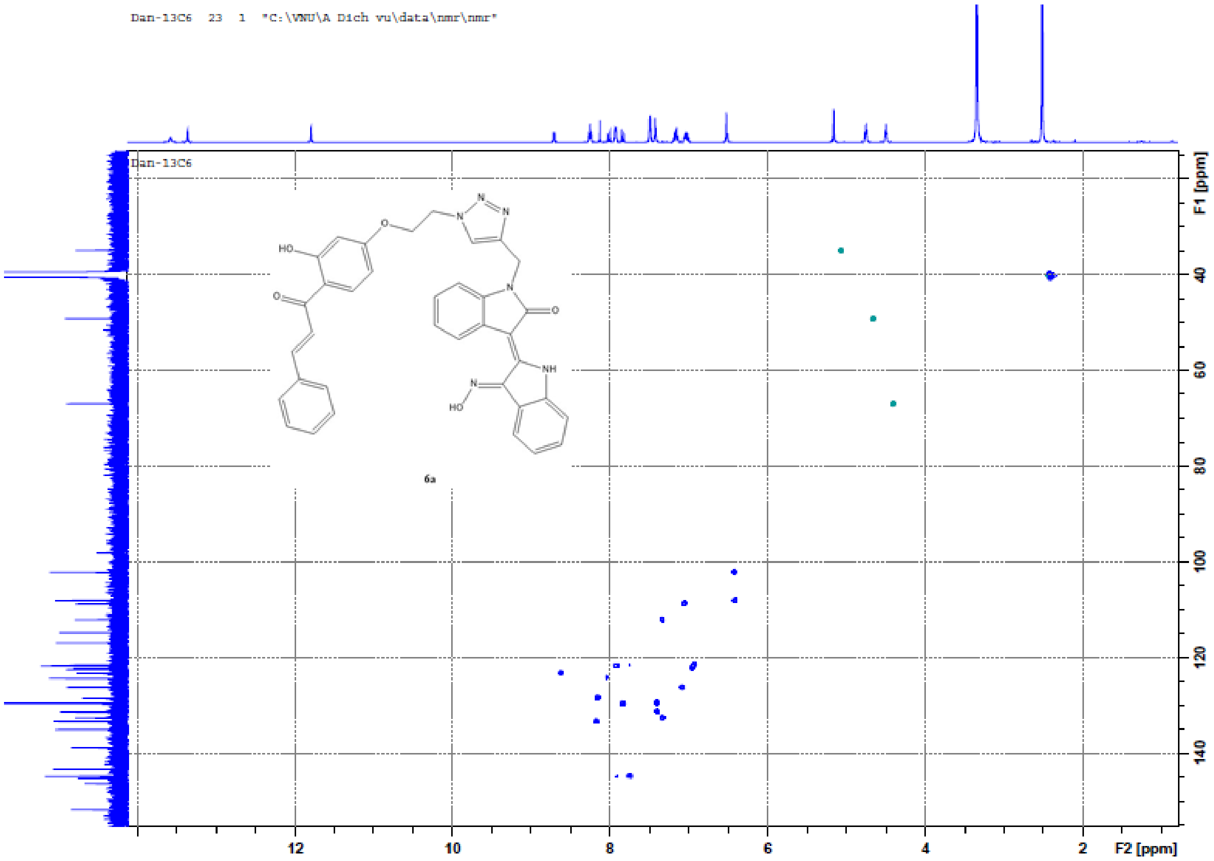

HSQC spectrum of compound **6a**

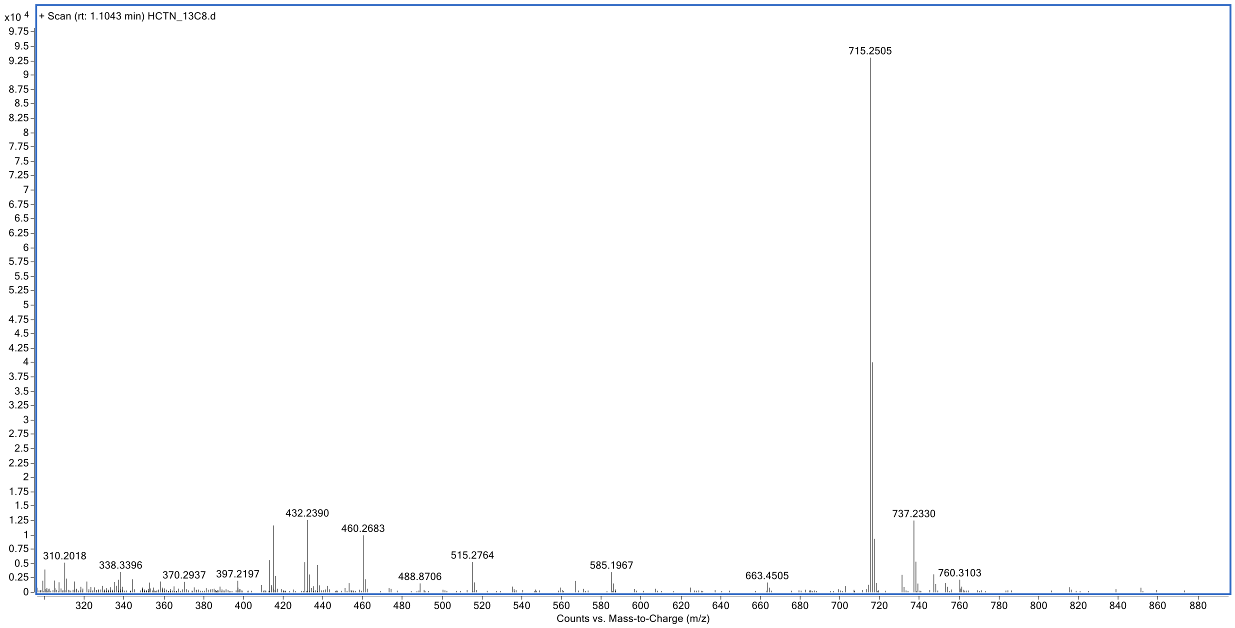

HR-MS(ESI) spectrum of compound **6b**

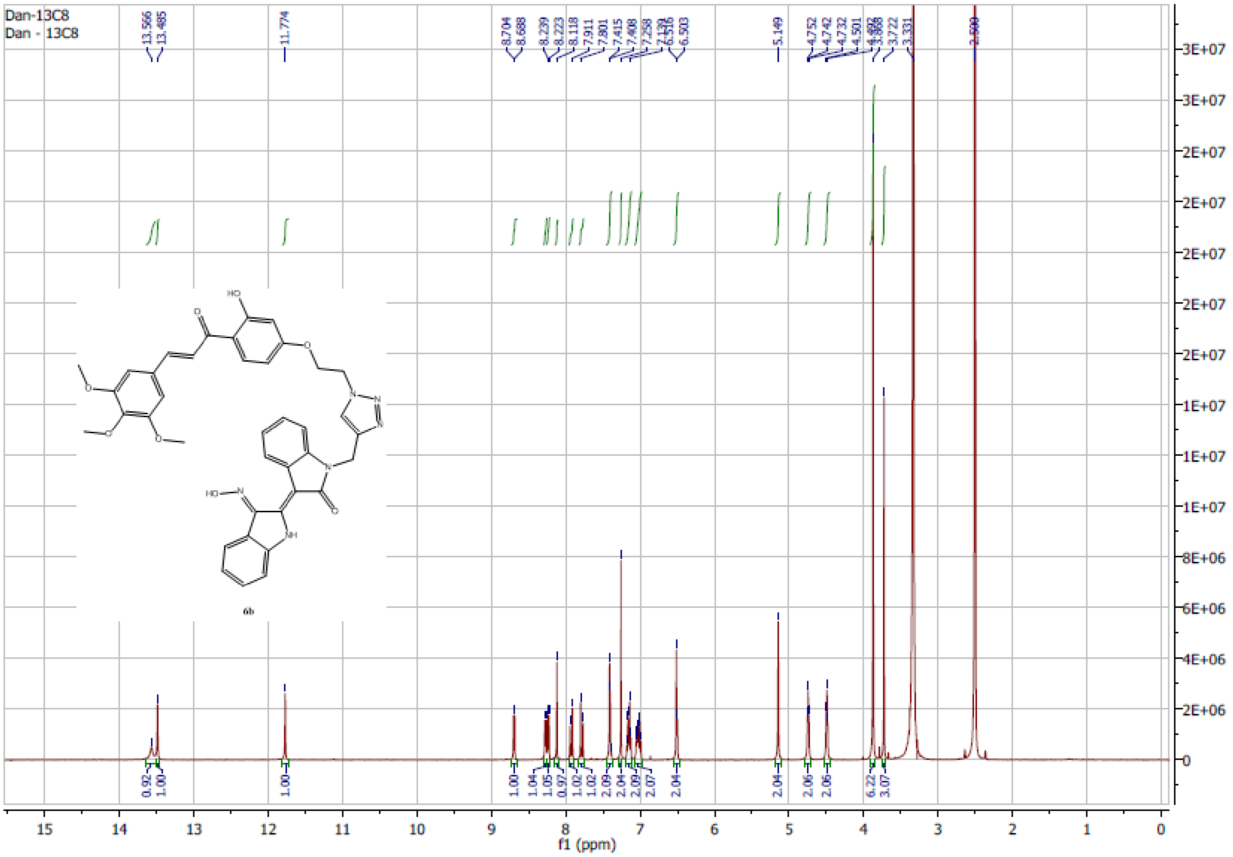

^{1}H-NMR spectrum of compound **6b**

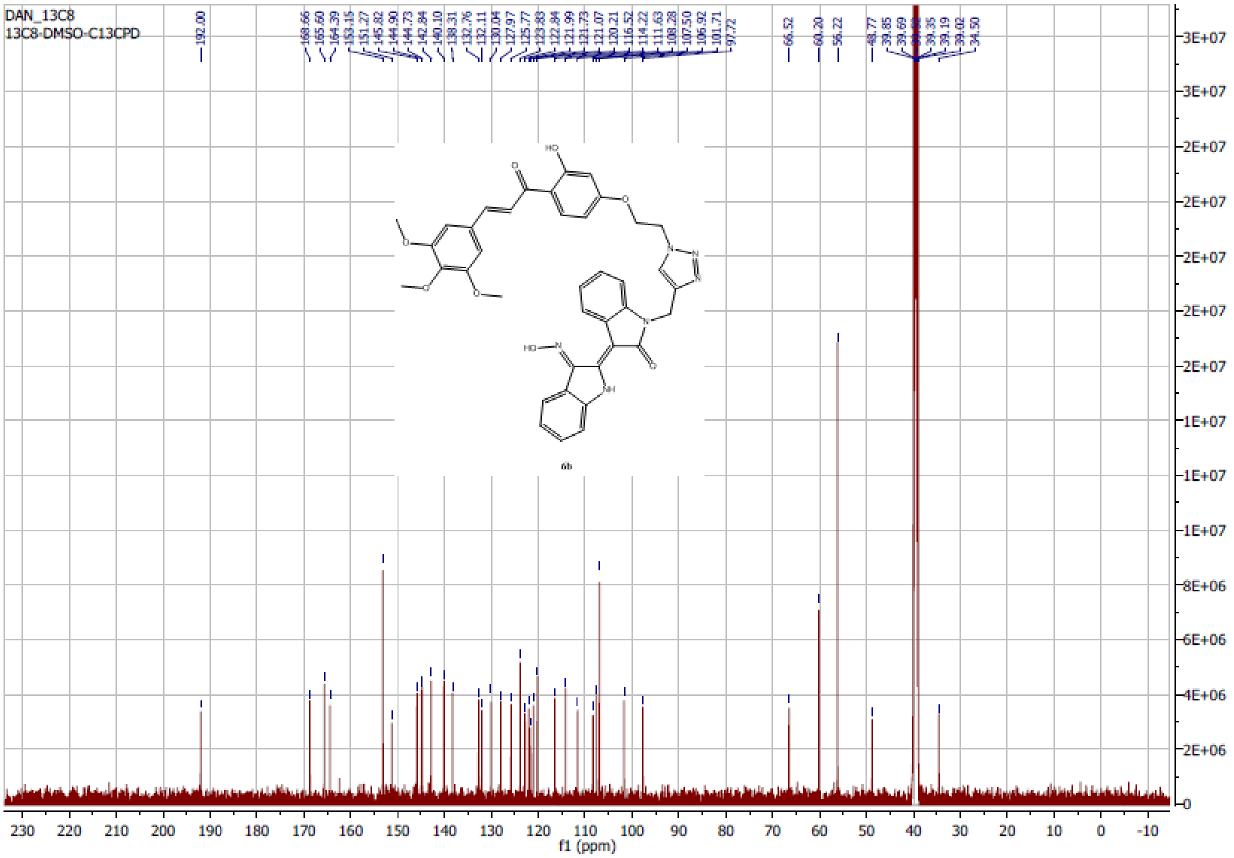

^{13}C-NMR spectrum of compound **6b**

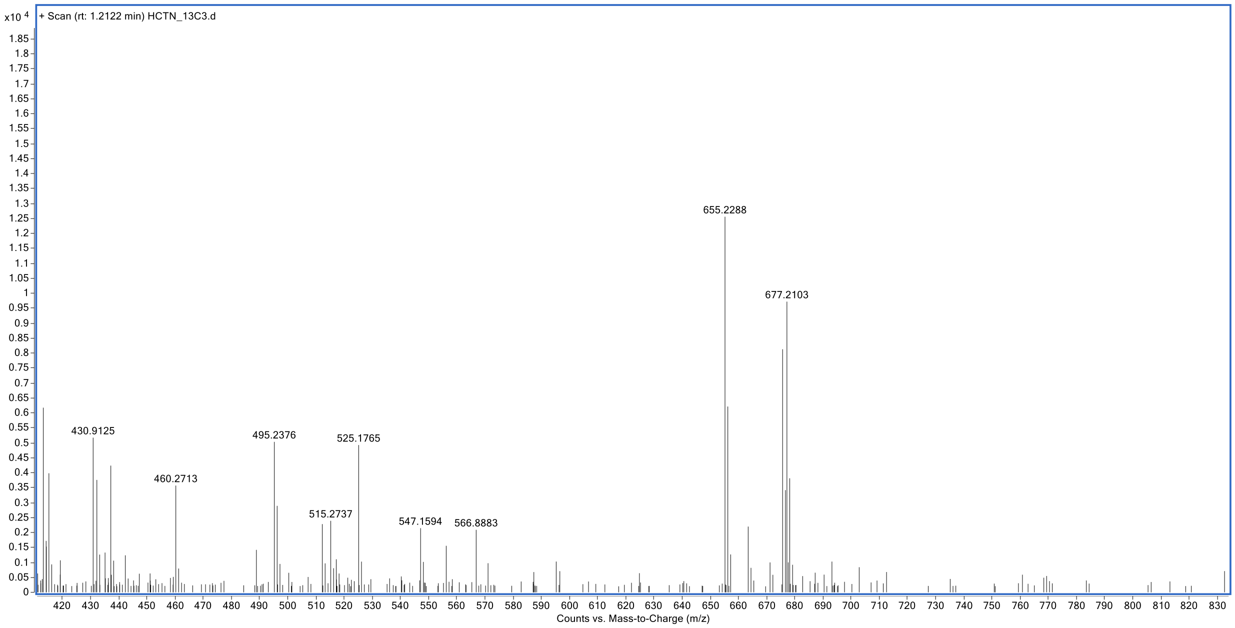

HR-MS(ESI) spectrum of compound **6c**

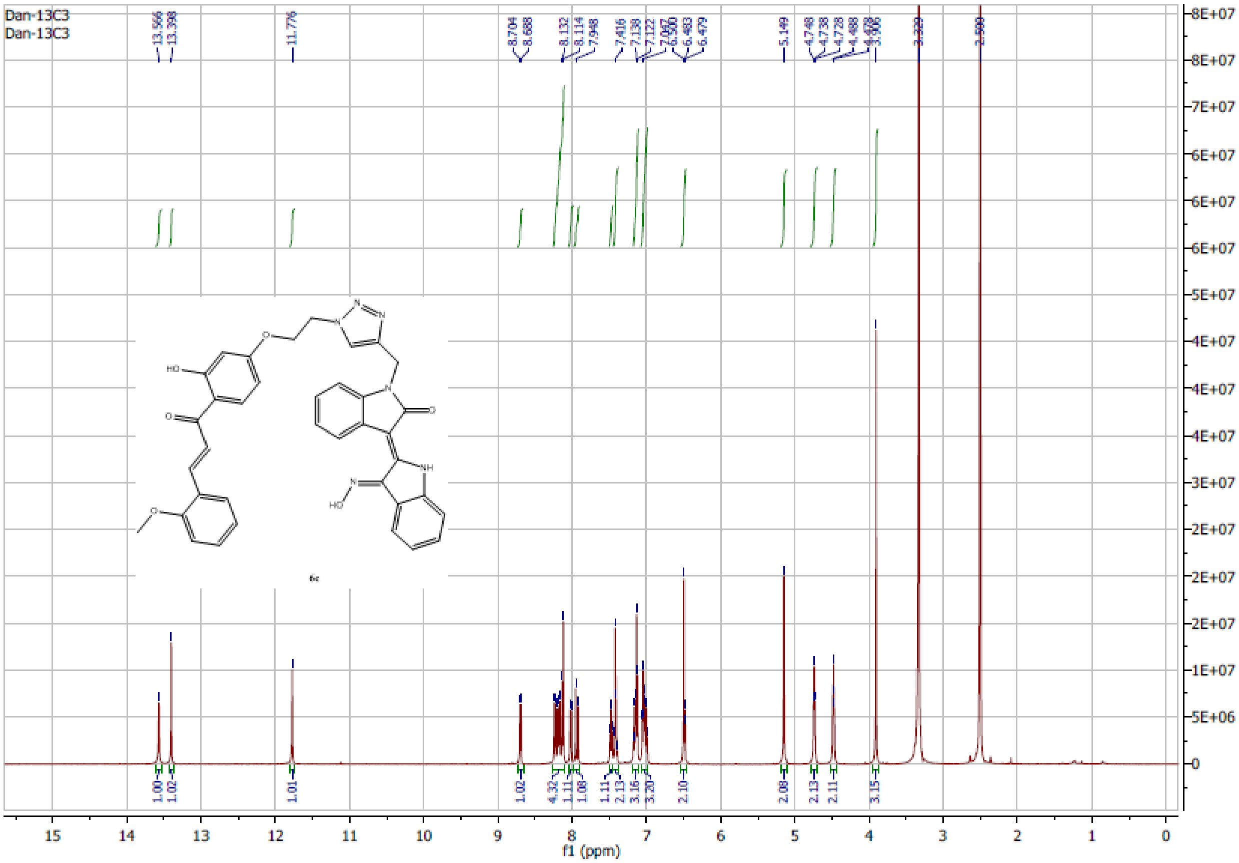

^{1}H-NMR spectrum of compound **6c**

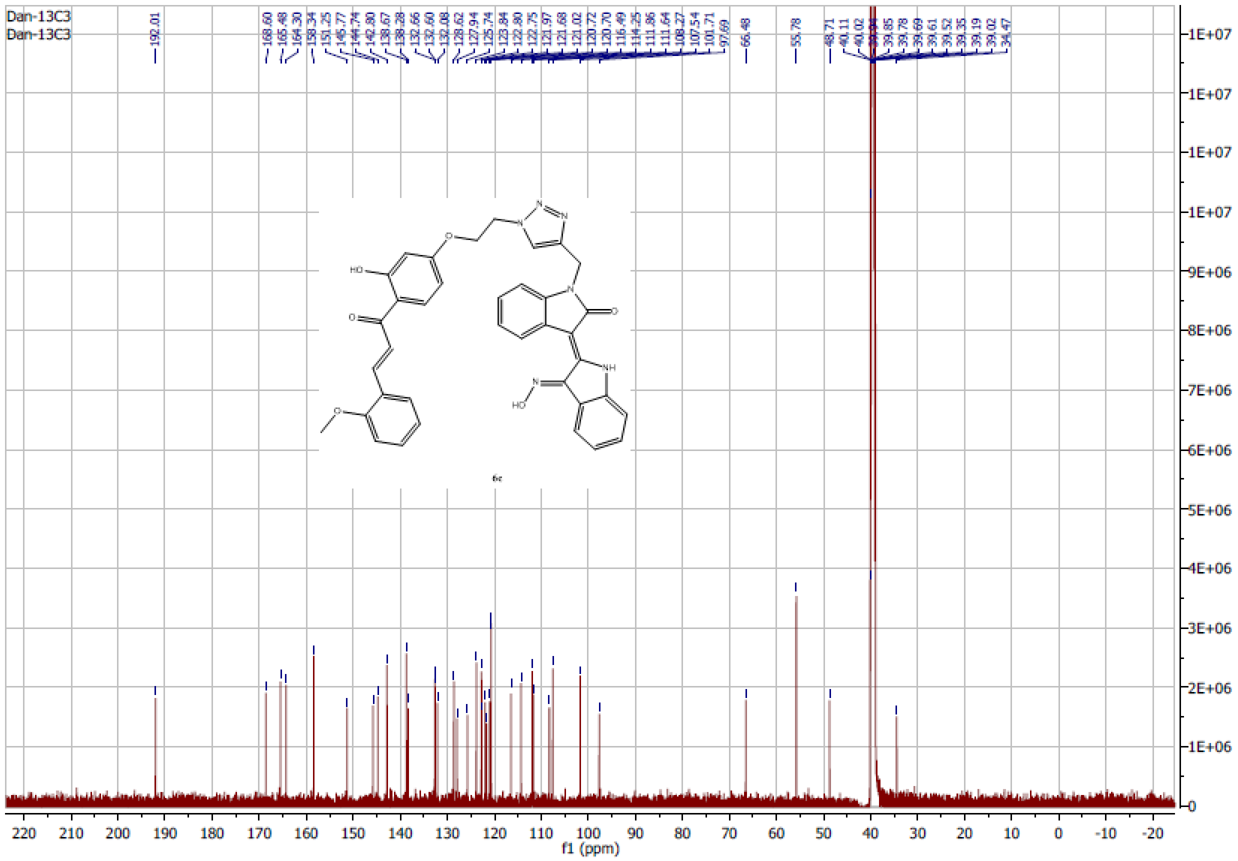

^{13}C-NMR spectrum of compound **6c**

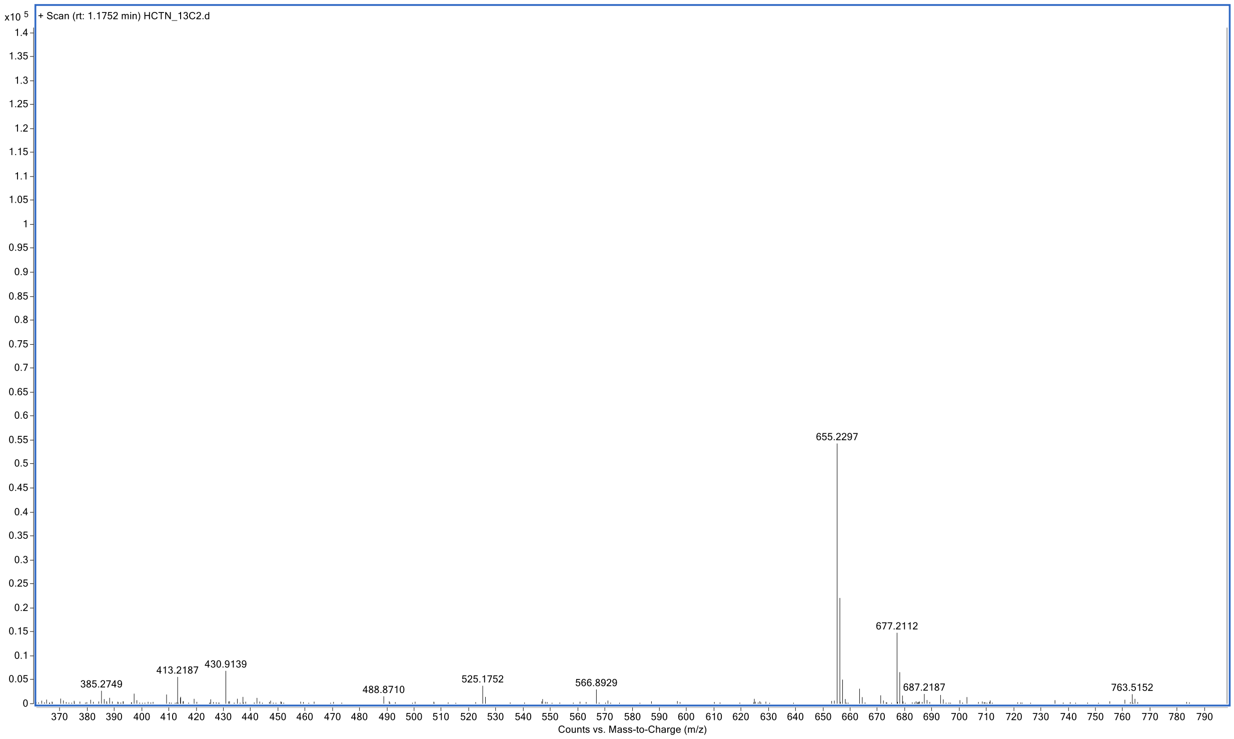

HR-MS(ESI) spectrum of compound **6d**

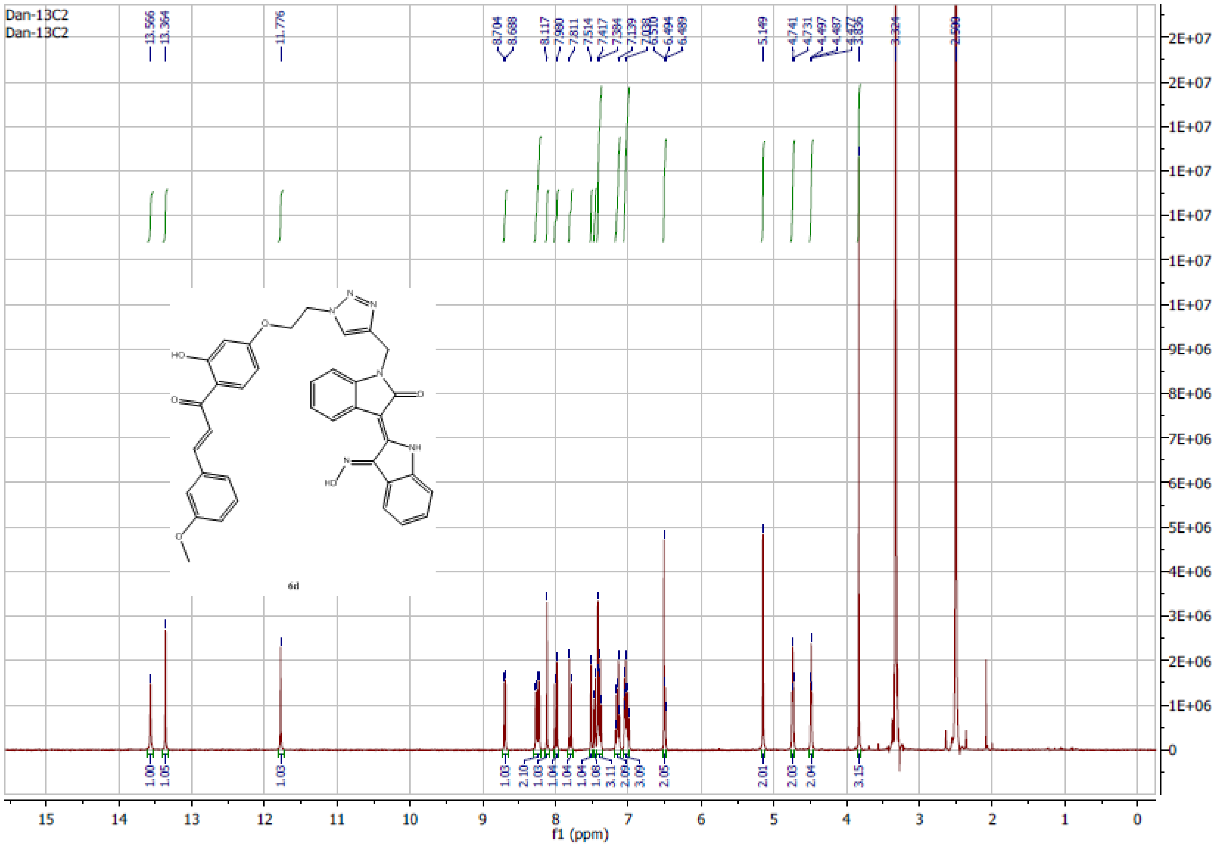

^{1}H-NMR spectrum of compound **6d**

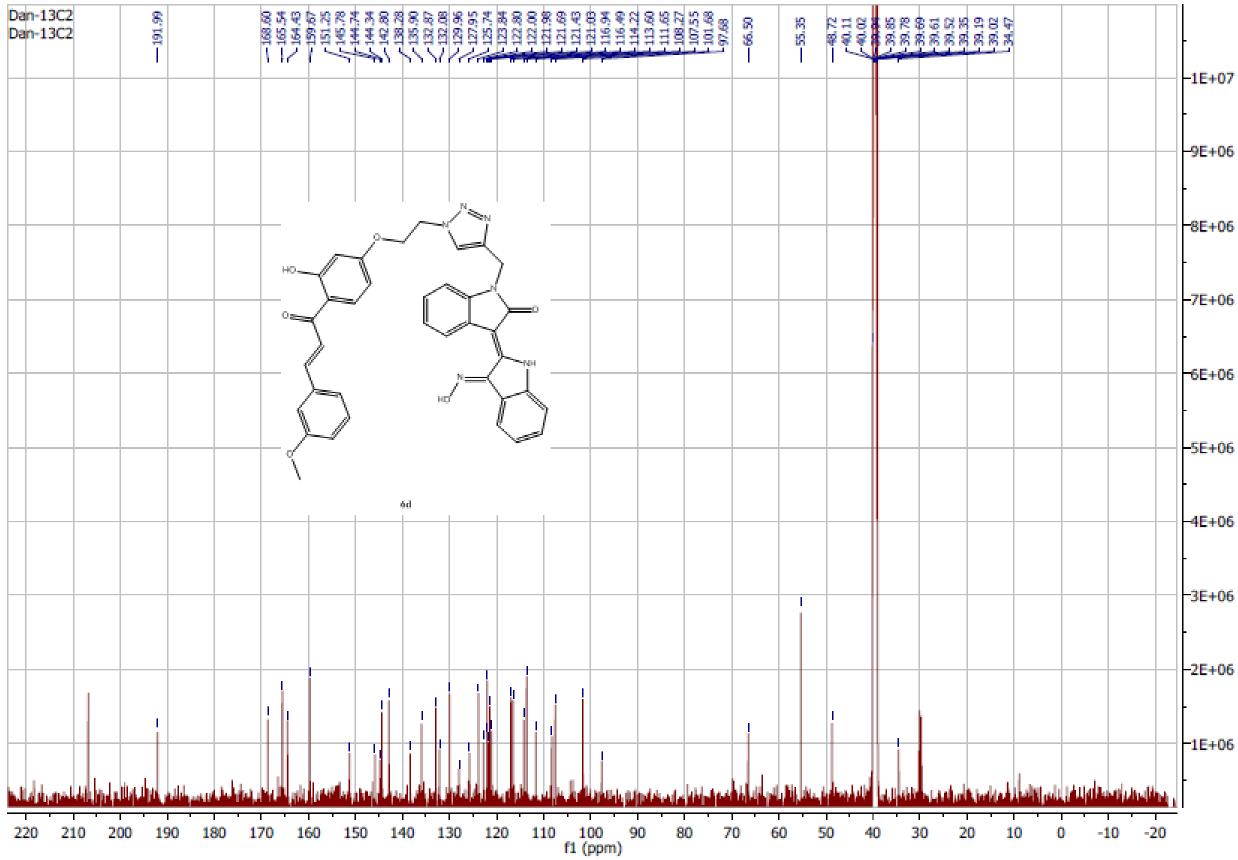

^{13}C-NMR spectrum of compound **6d**

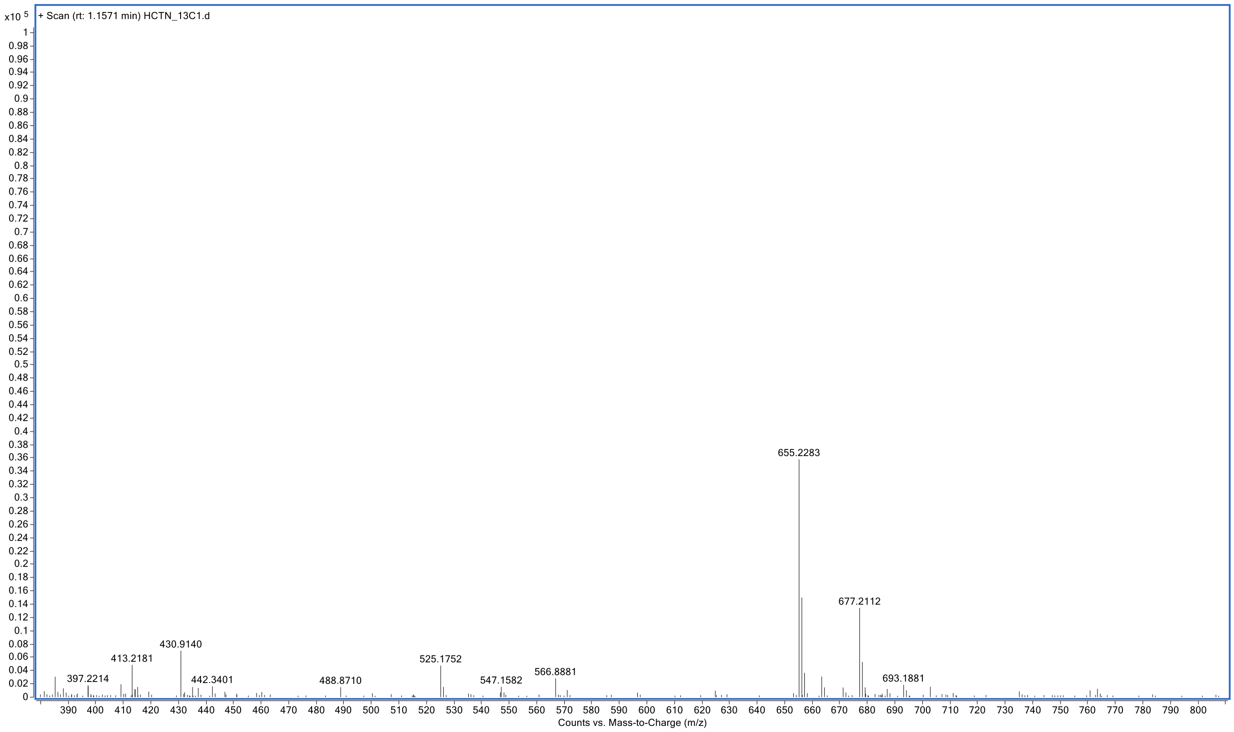

HR-MS(ESI) spectrum of compound **6e**

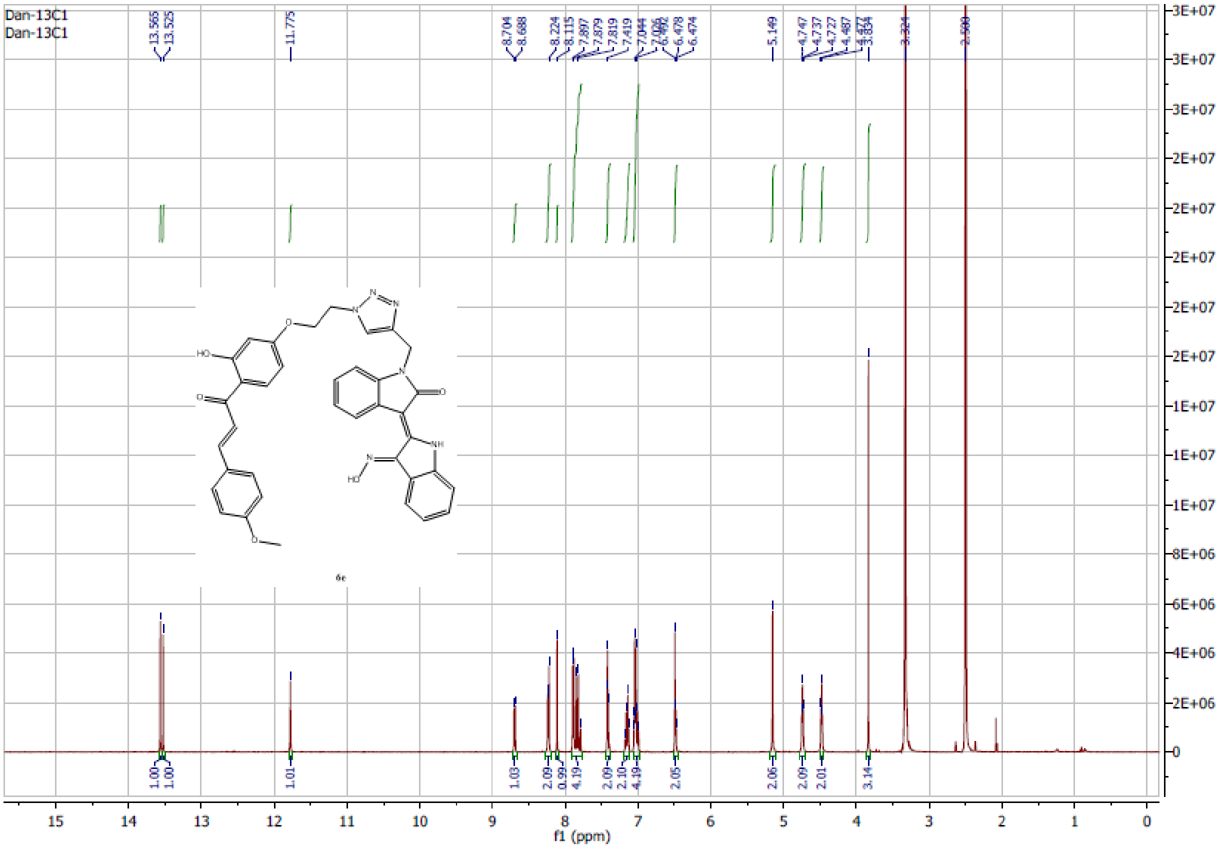

^{1}H-NMR spectrum of compound **6e**

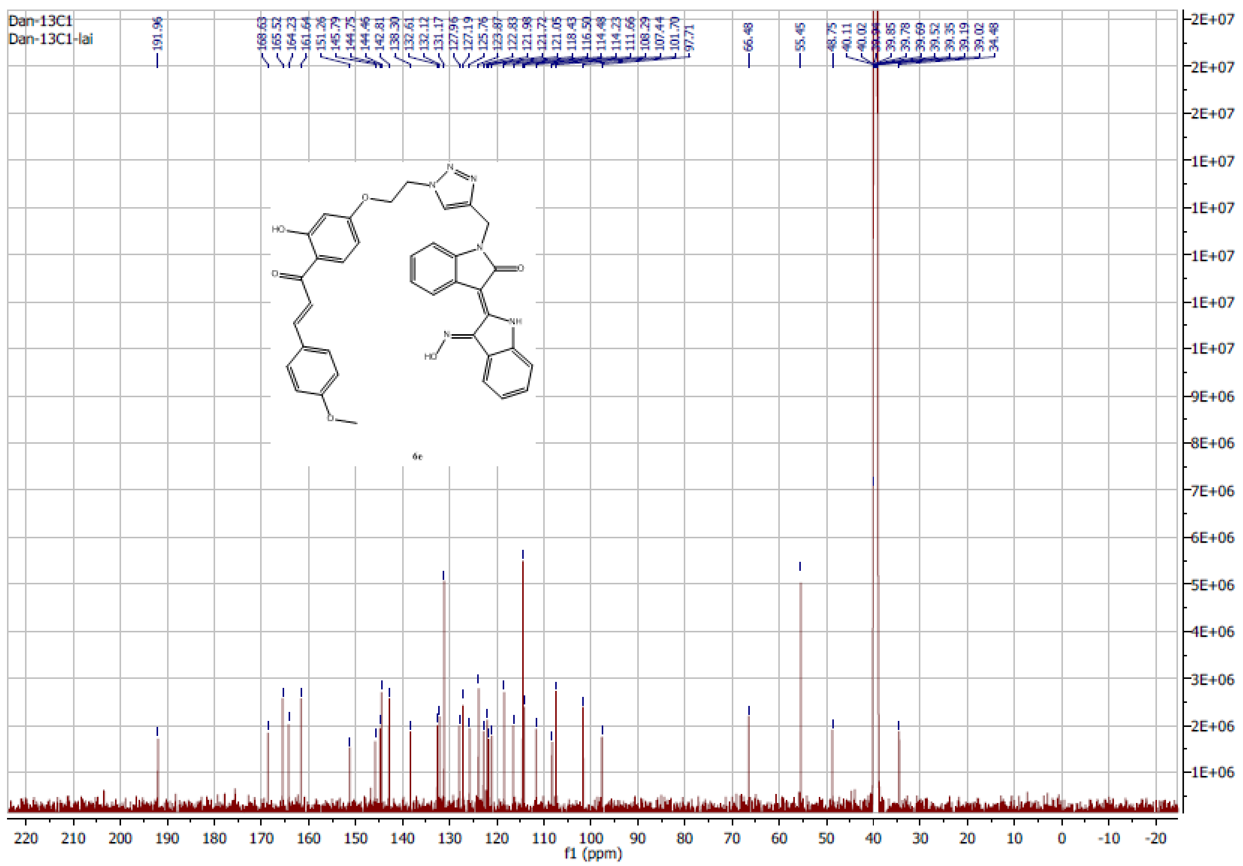

^{13}C-NMR spectrum of compound **6e**

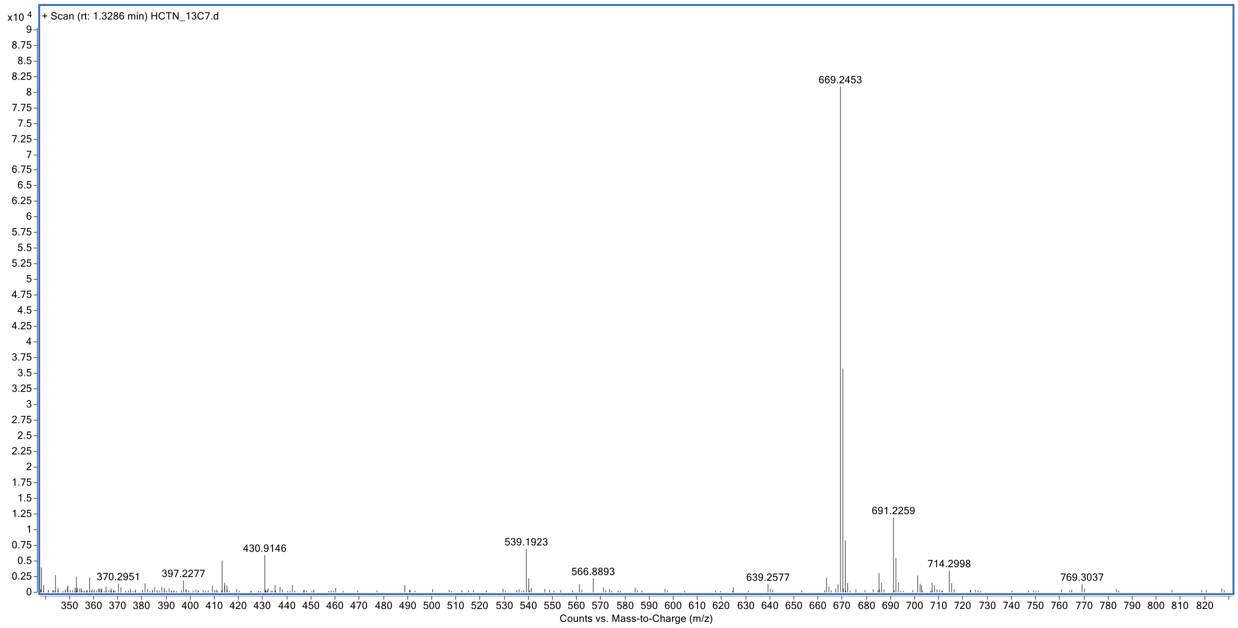

HR-MS(ESI) spectrum of compound **6f**

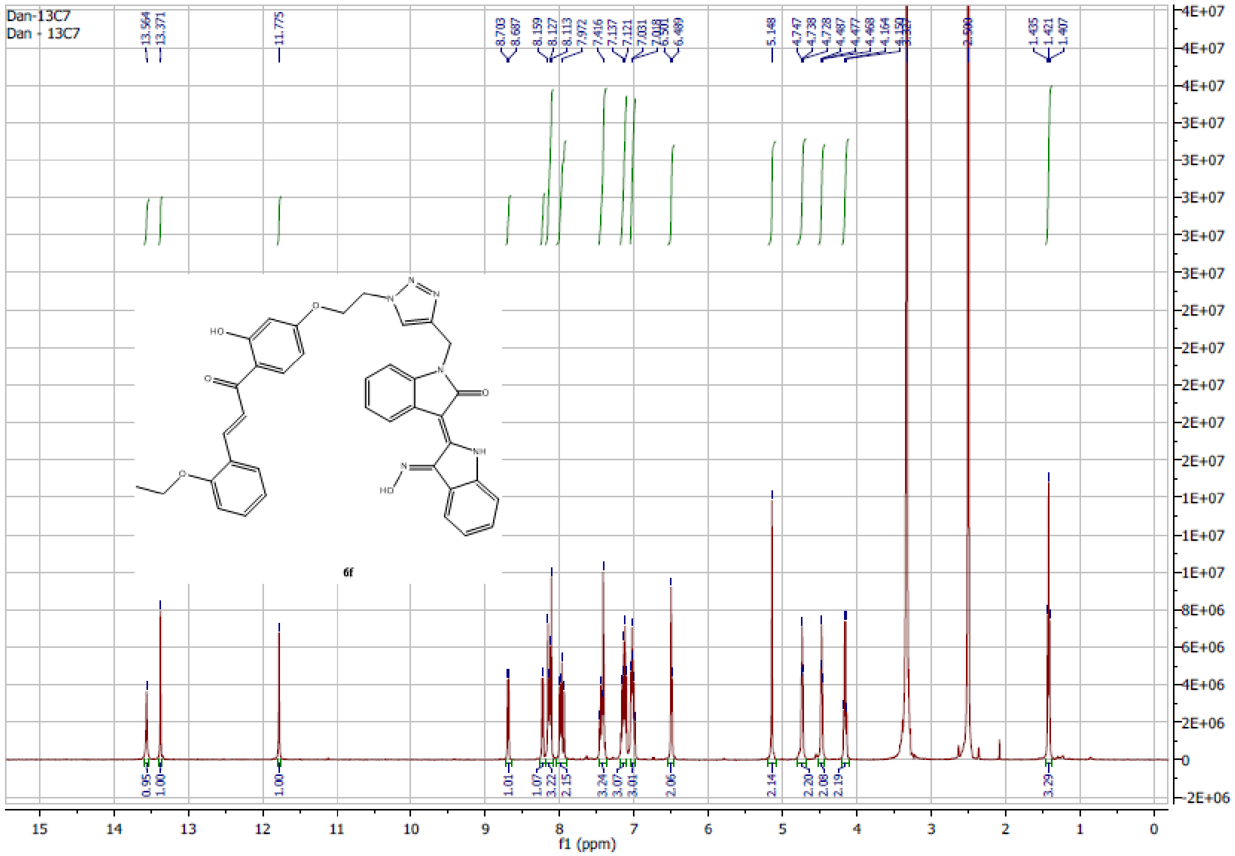

^{1}H-NMR spectrum of compound **6f**

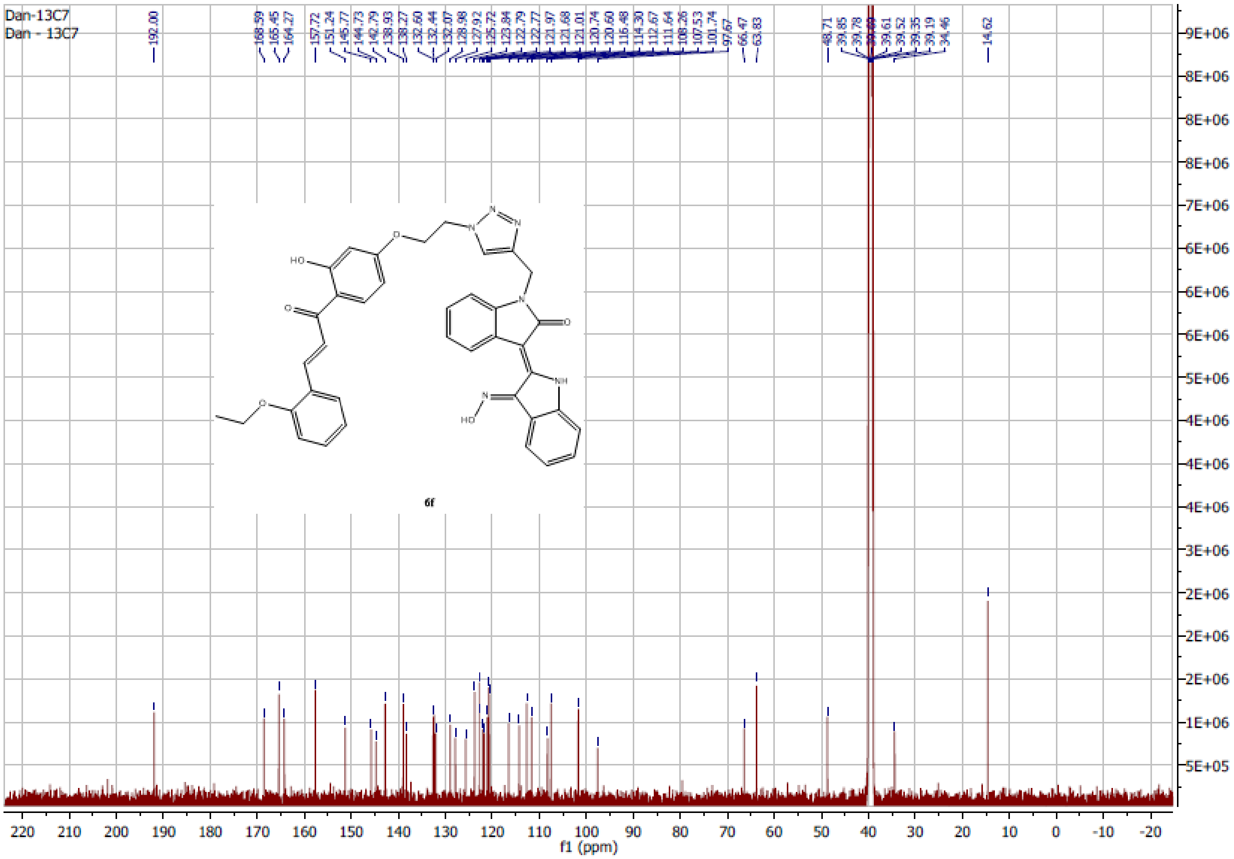

^{13}C-NMR spectrum of compound **6f**

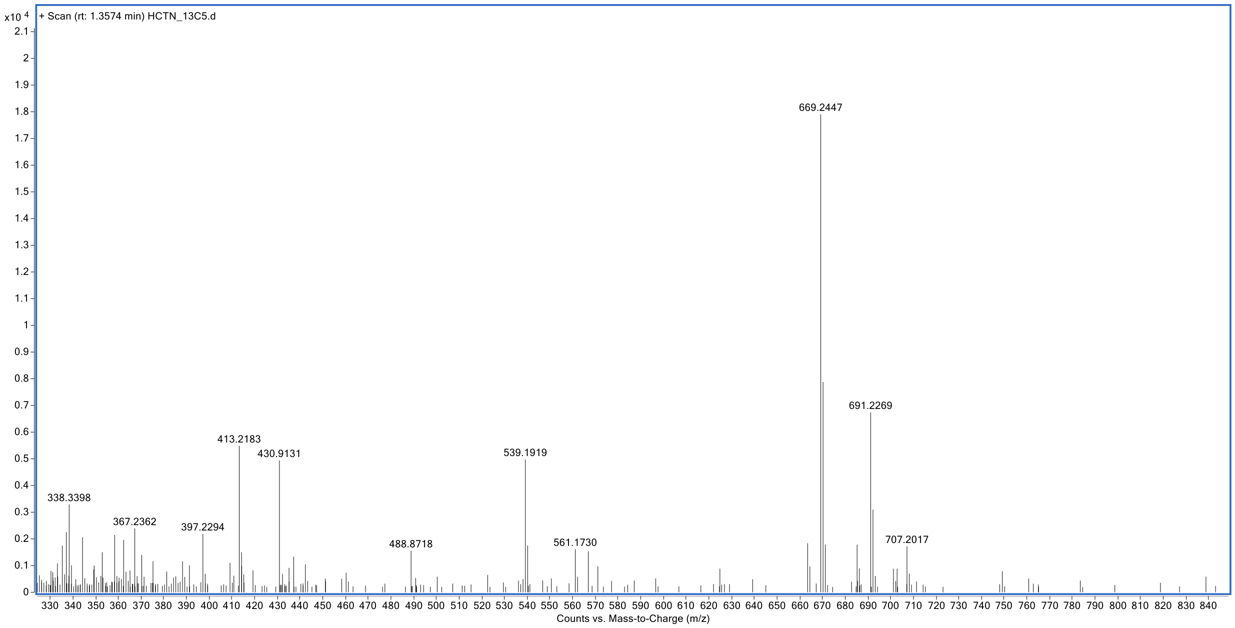

HR-MS(ESI) spectrum of compound **6g**

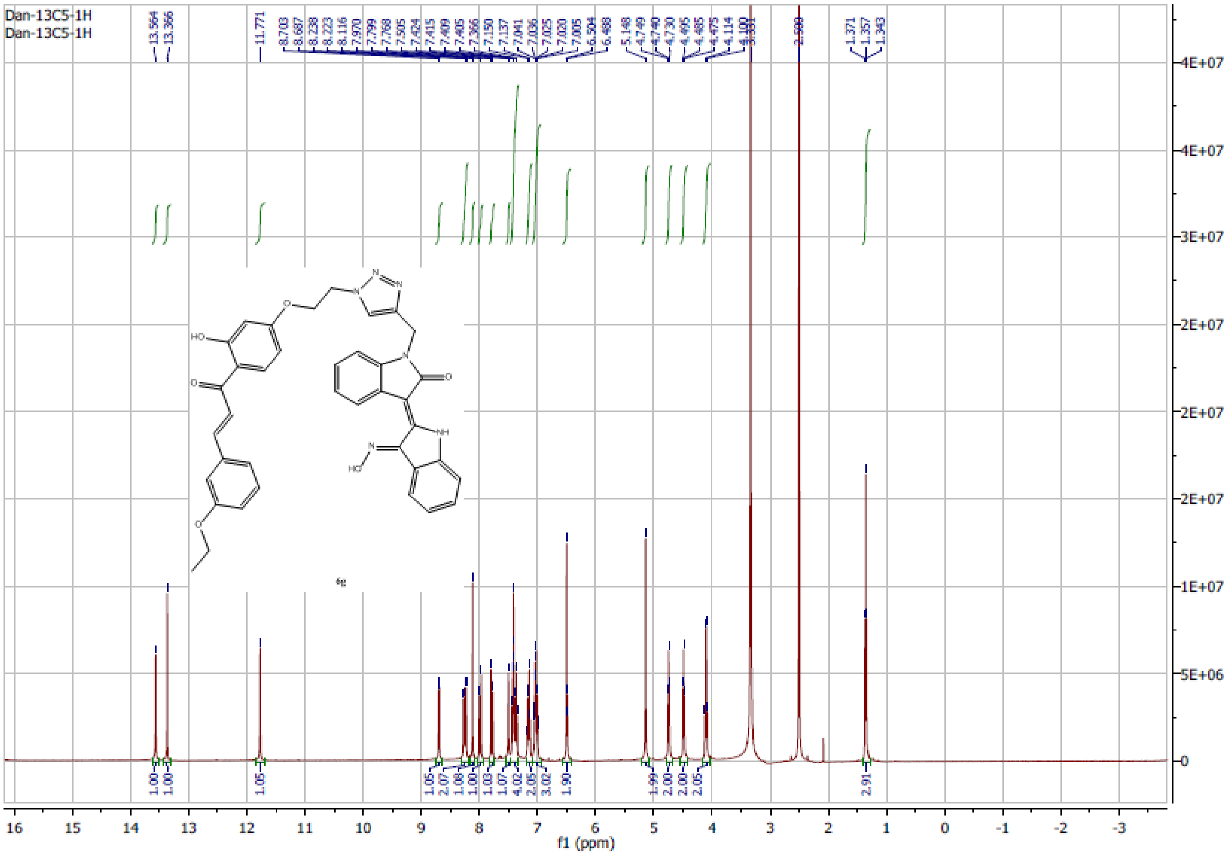

^{1}H-NMR spectrum of compound **6g**

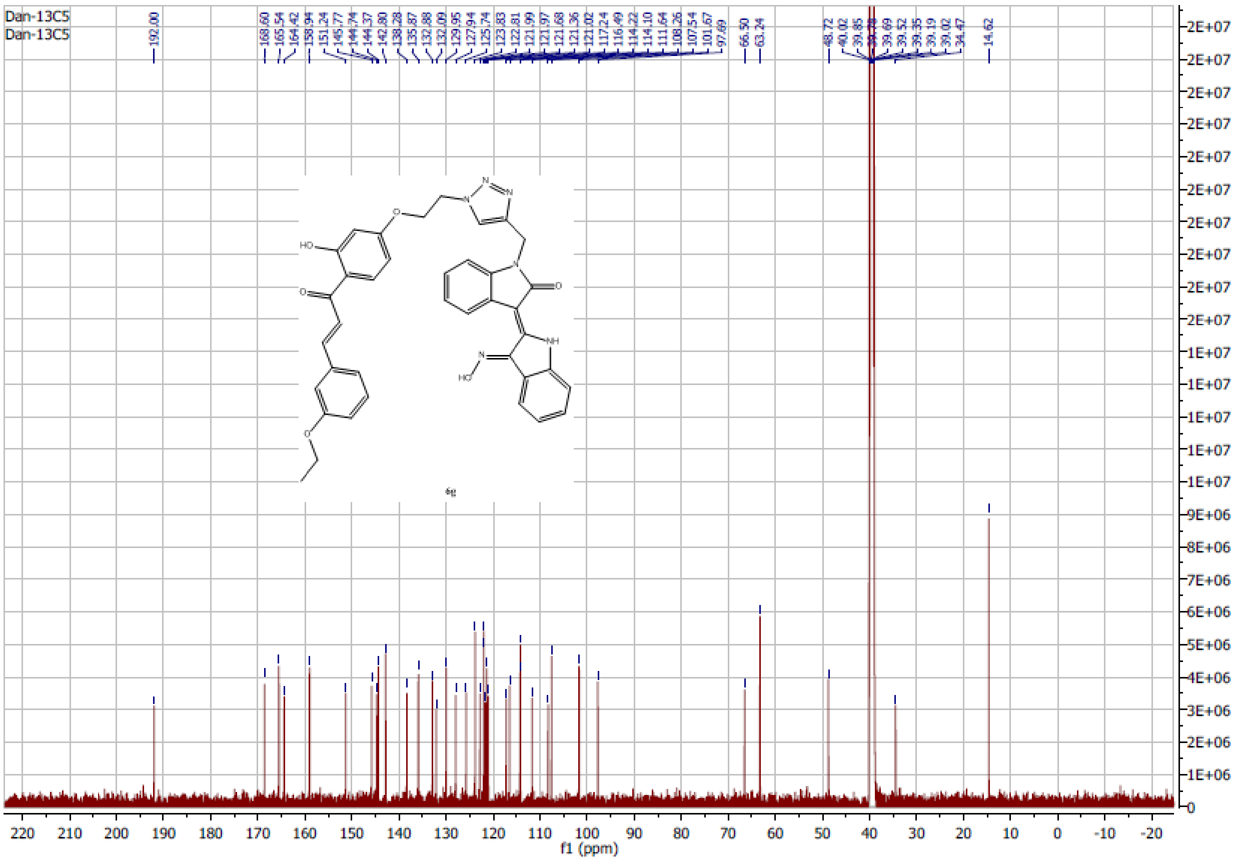

^{13}C-NMR spectrum of compound **6g**

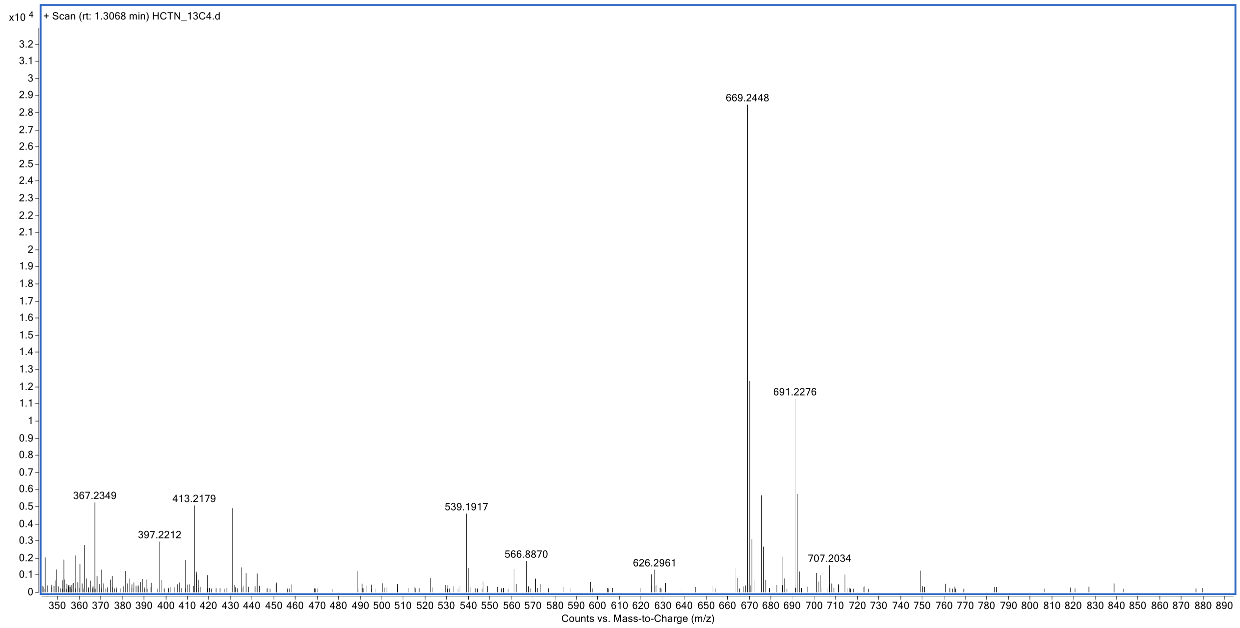

HR-MS(ESI) spectrum of compound **6h**

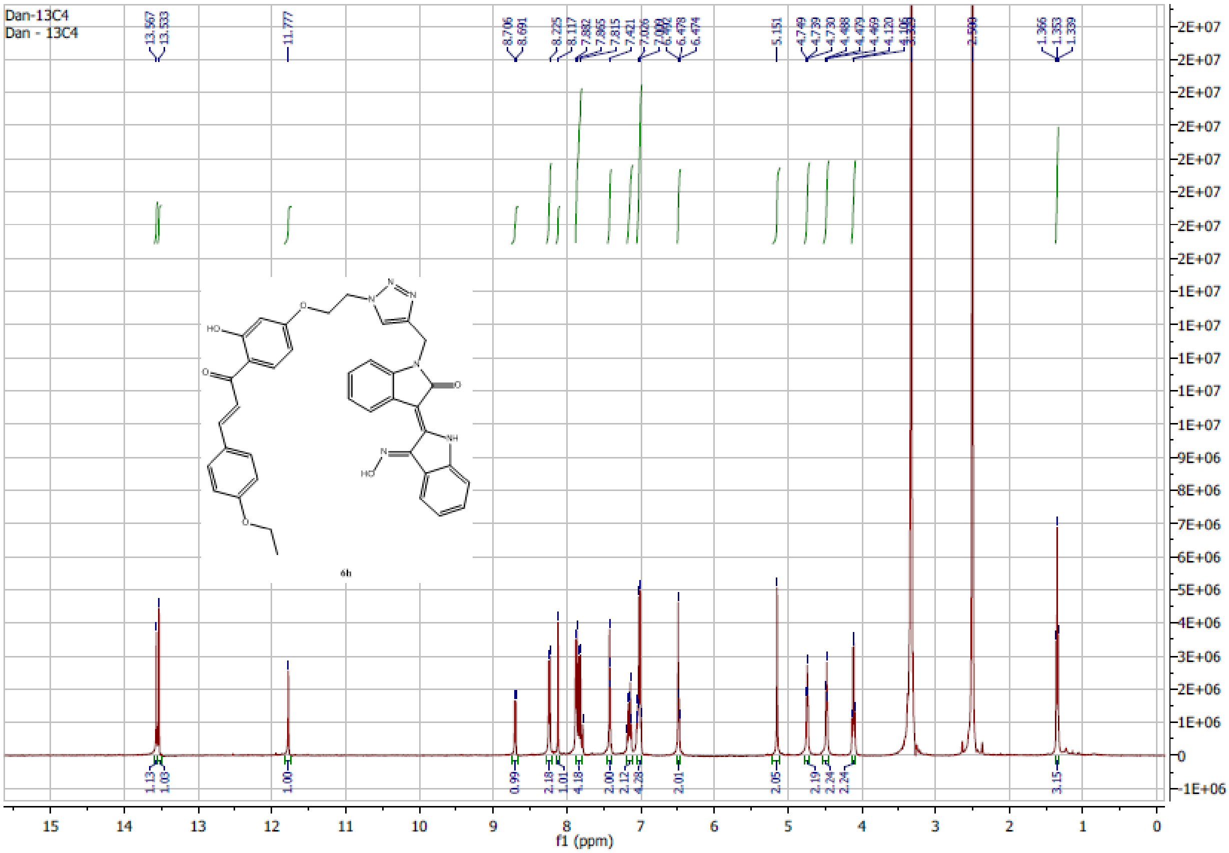

^{1}H-NMR spectrum of compound **6h**

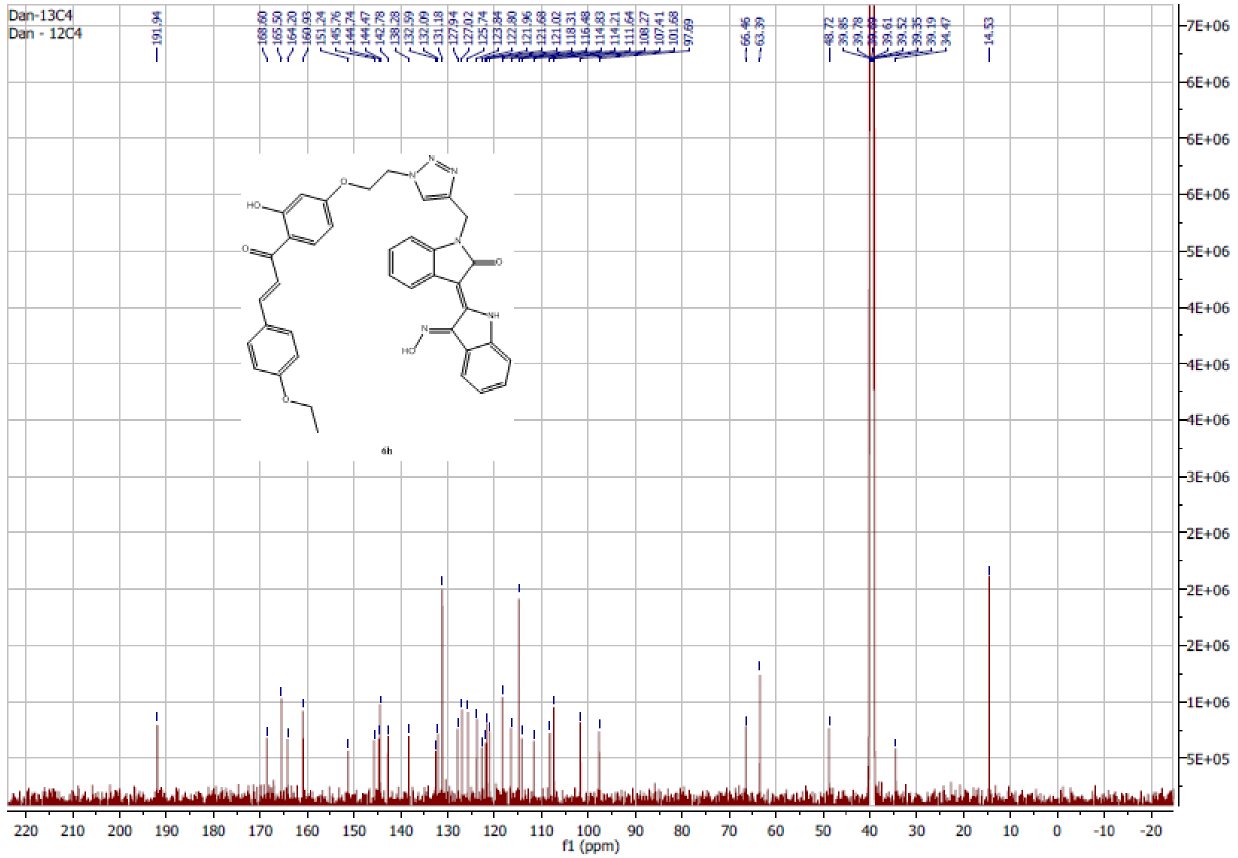

^{13}C-NMR spectrum of compound **6h**

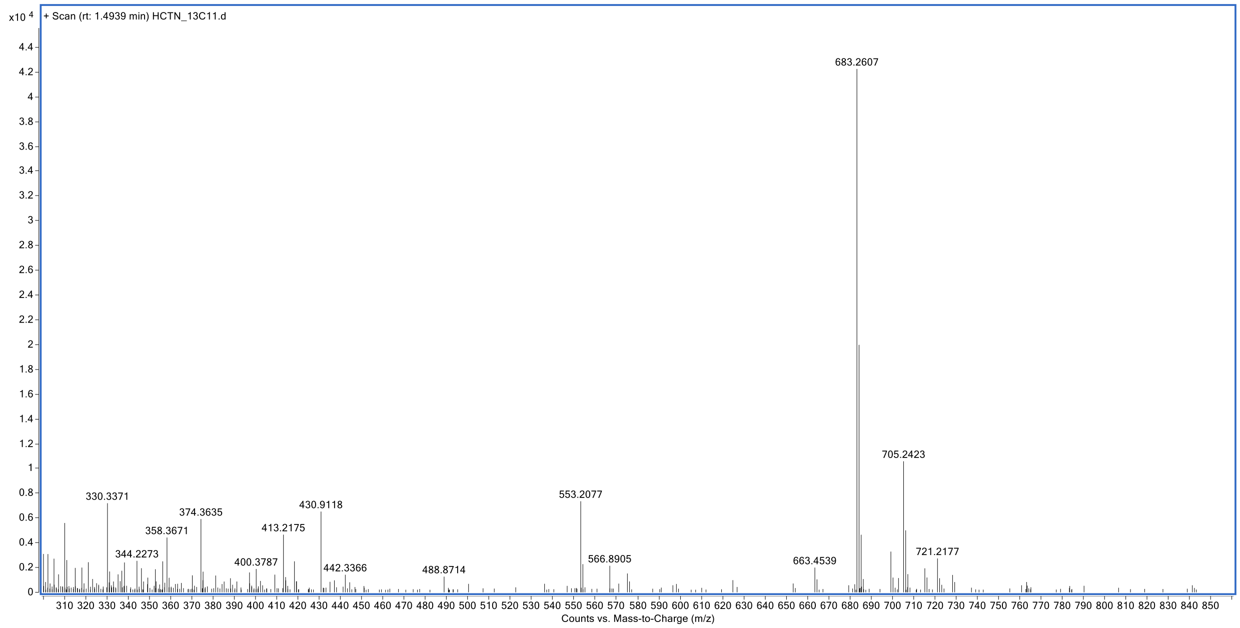

HR-MS(ESI) spectrum of compound **6i**

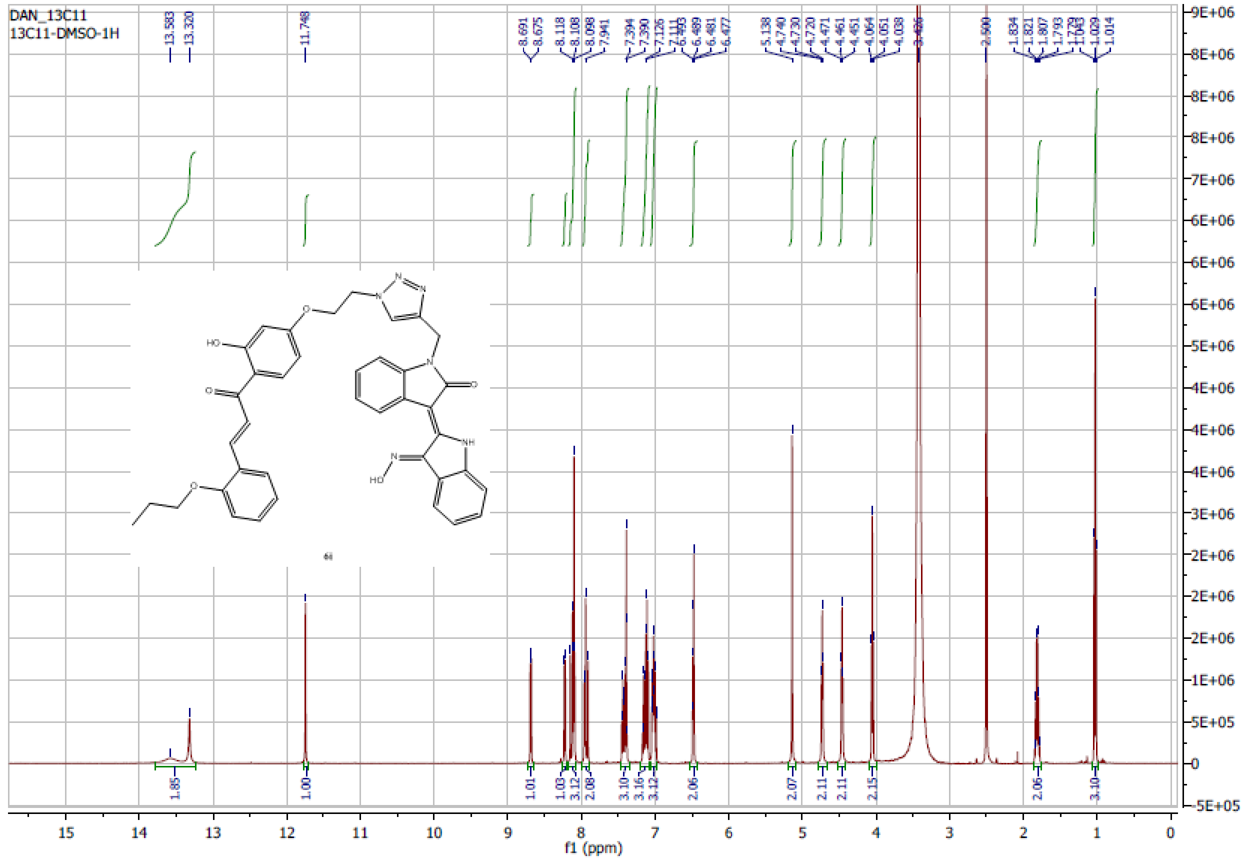

^{1}H-NMR spectrum of compound **6i**

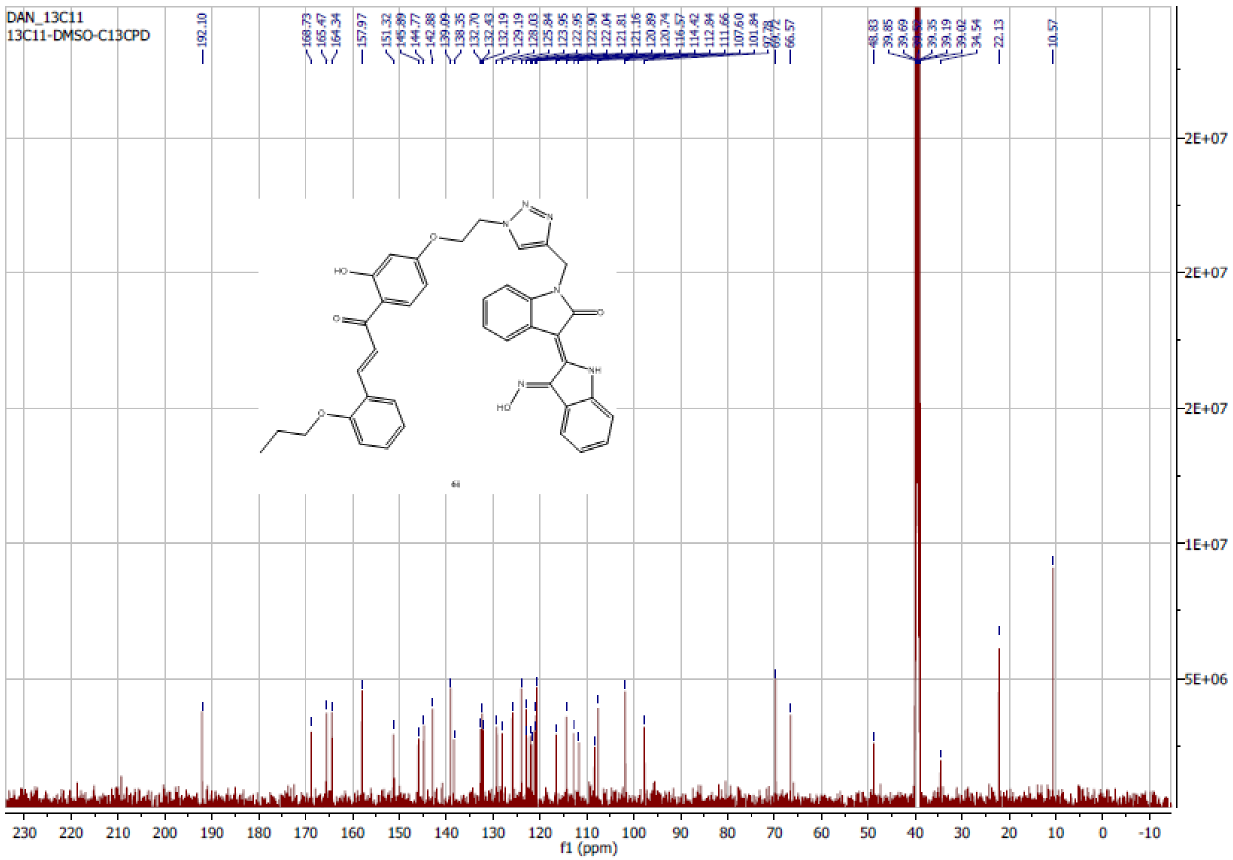

^{13}C-NMR spectrum of compound **6i**

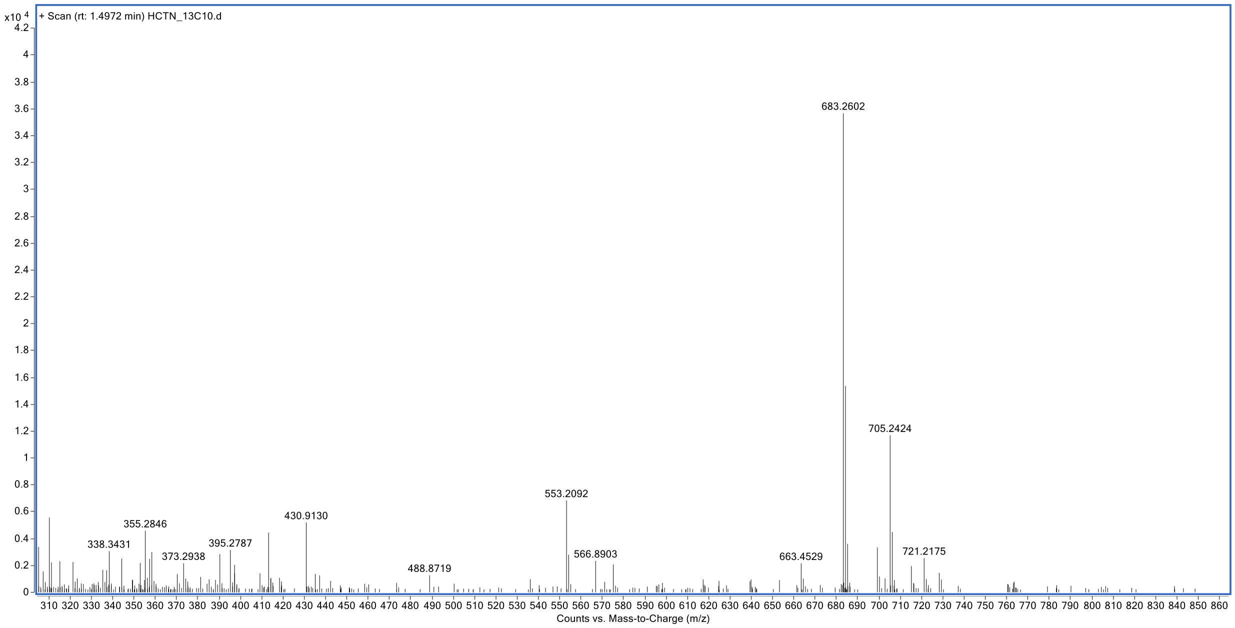

HR-MS(ESI) spectrum of compound **6k**

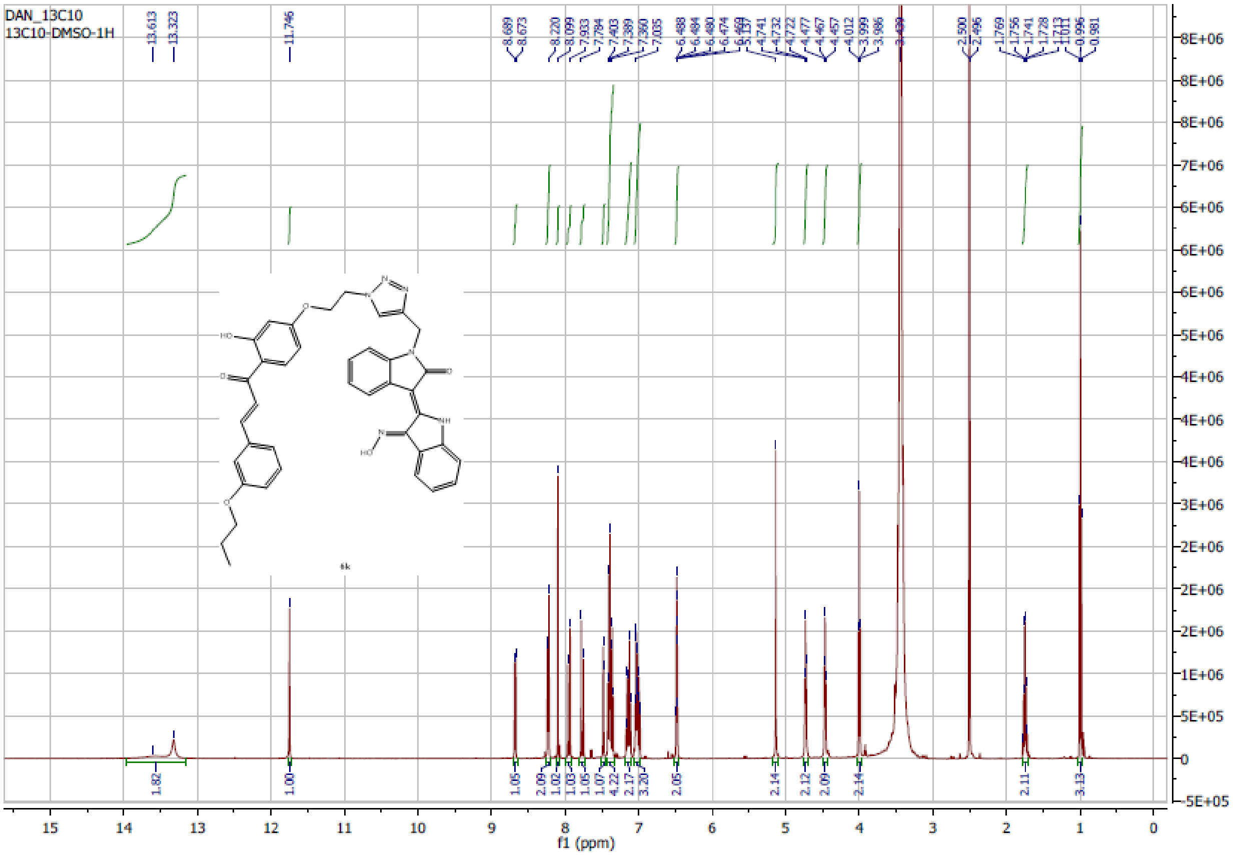

^{1}H-NMR spectrum of compound **6k**

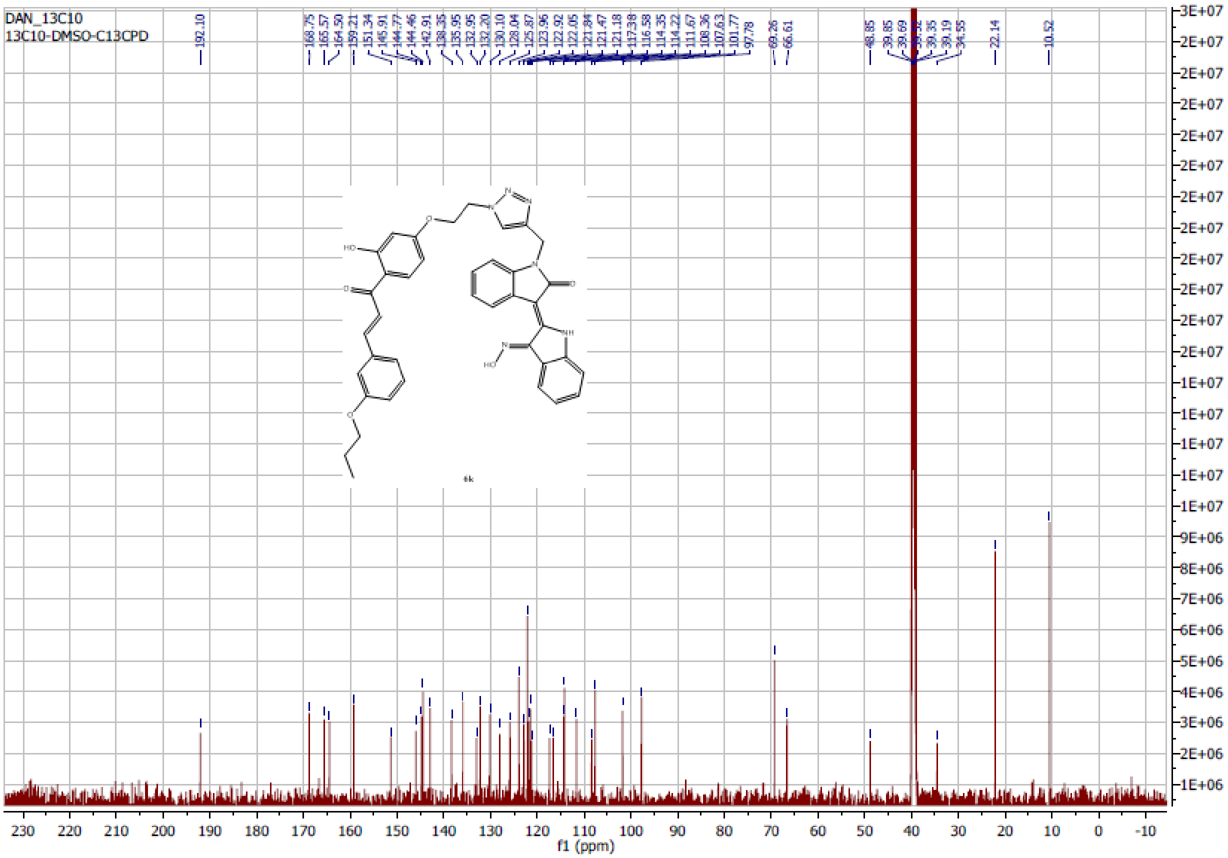

^{13}C-NMR spectrum of compound **6k**

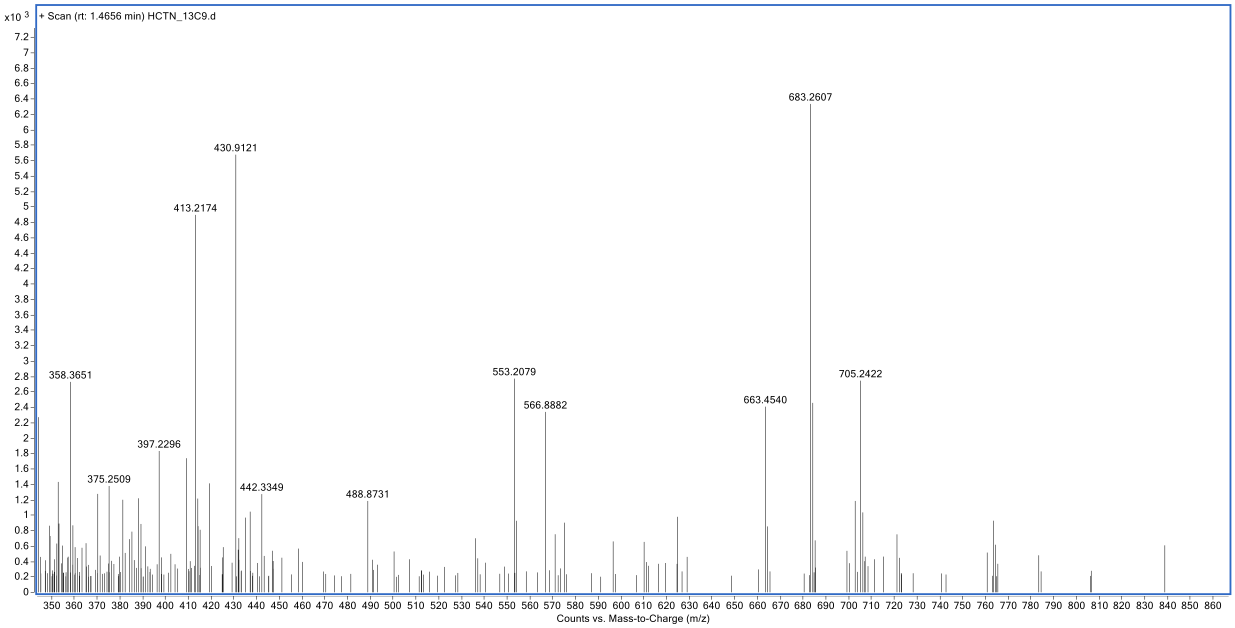

HR-MS(ESI) spectrum of compound **6l**

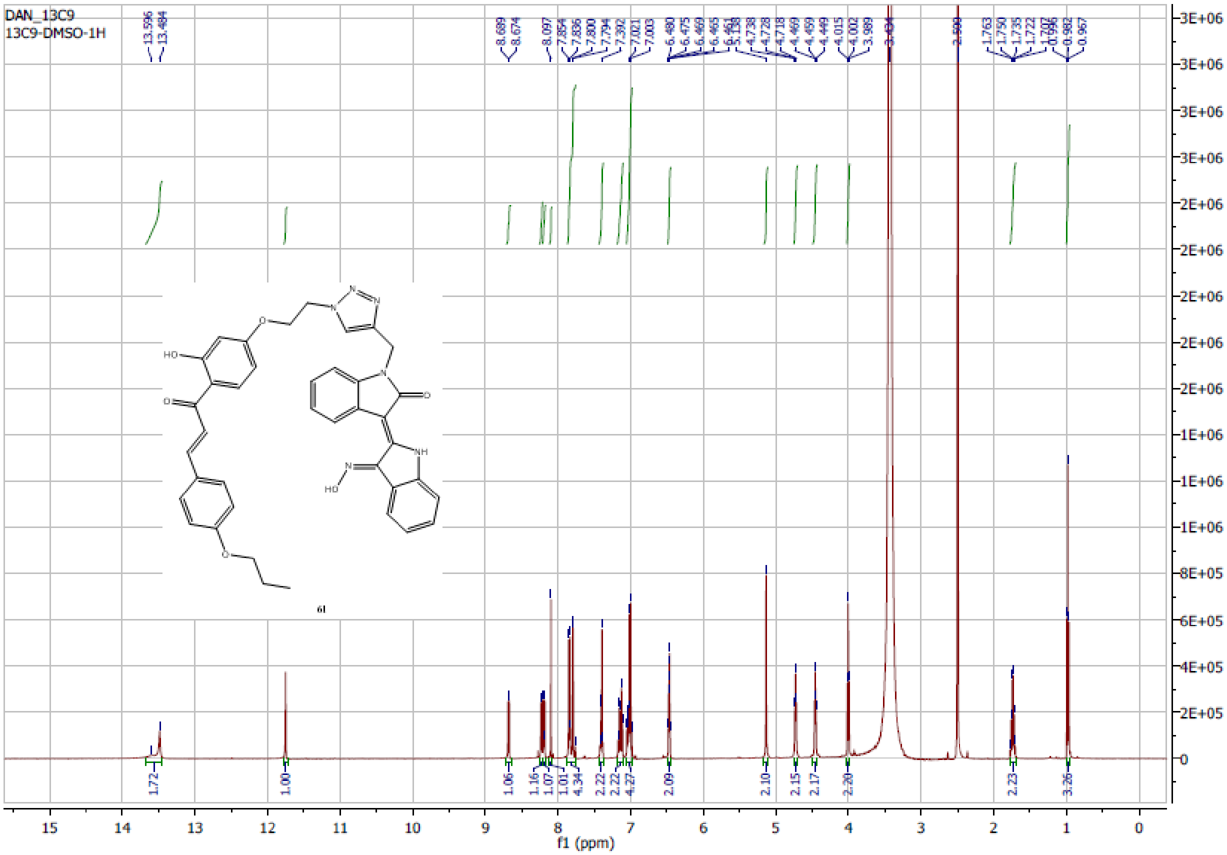

^{1}H-NMR spectrum of compound **6l**

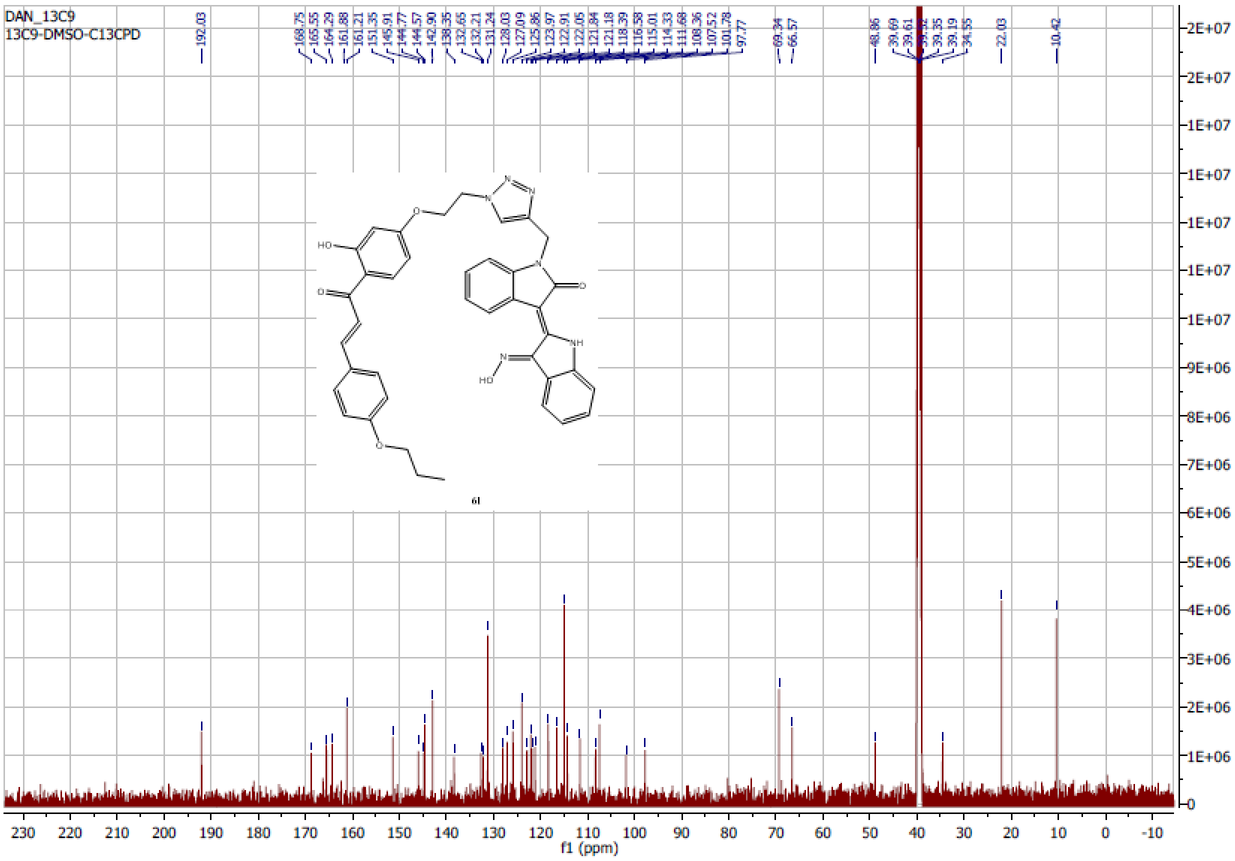

^{13}C-NMR spectrum of compound **6l**

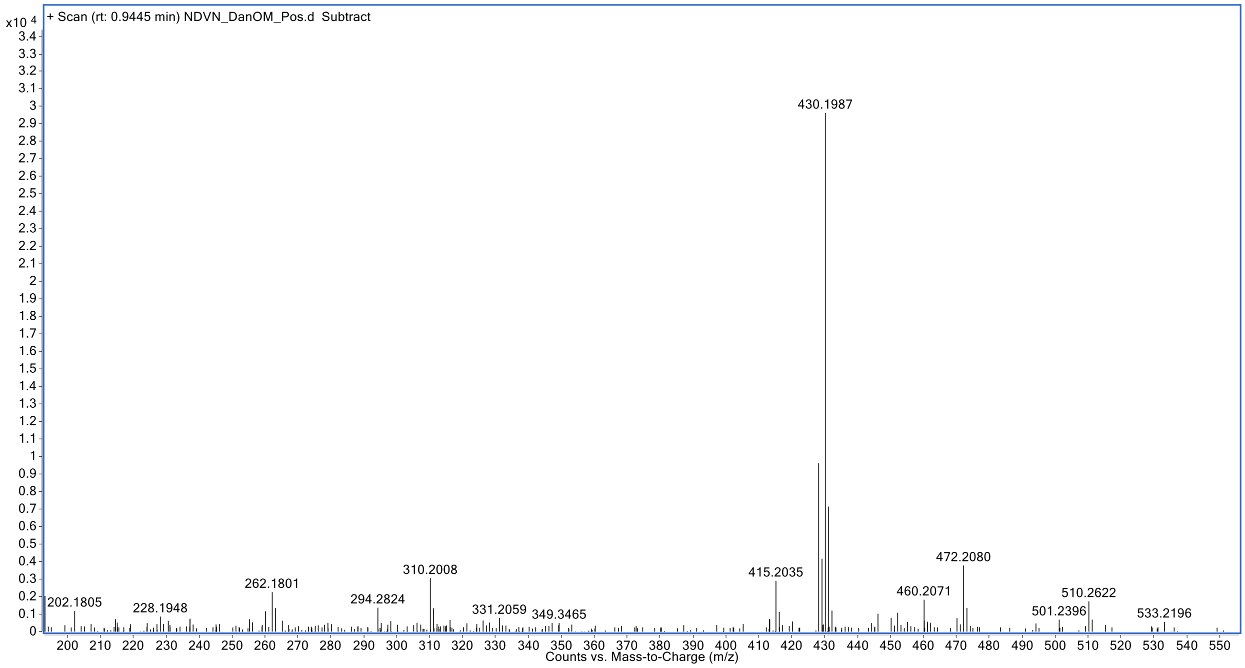

HR-MS(ESI) spectrum of compound **6m**

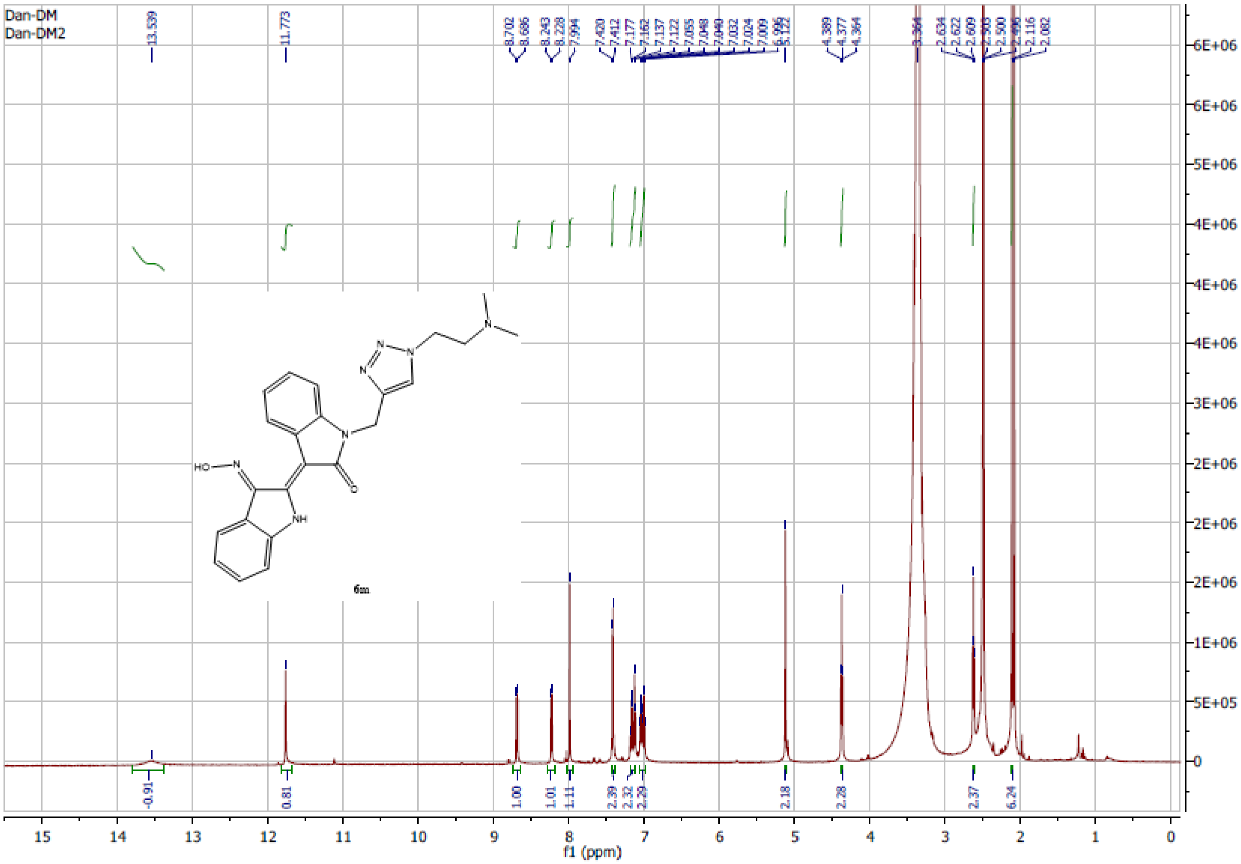

^{1}H-NMR spectrum of compound **6m**

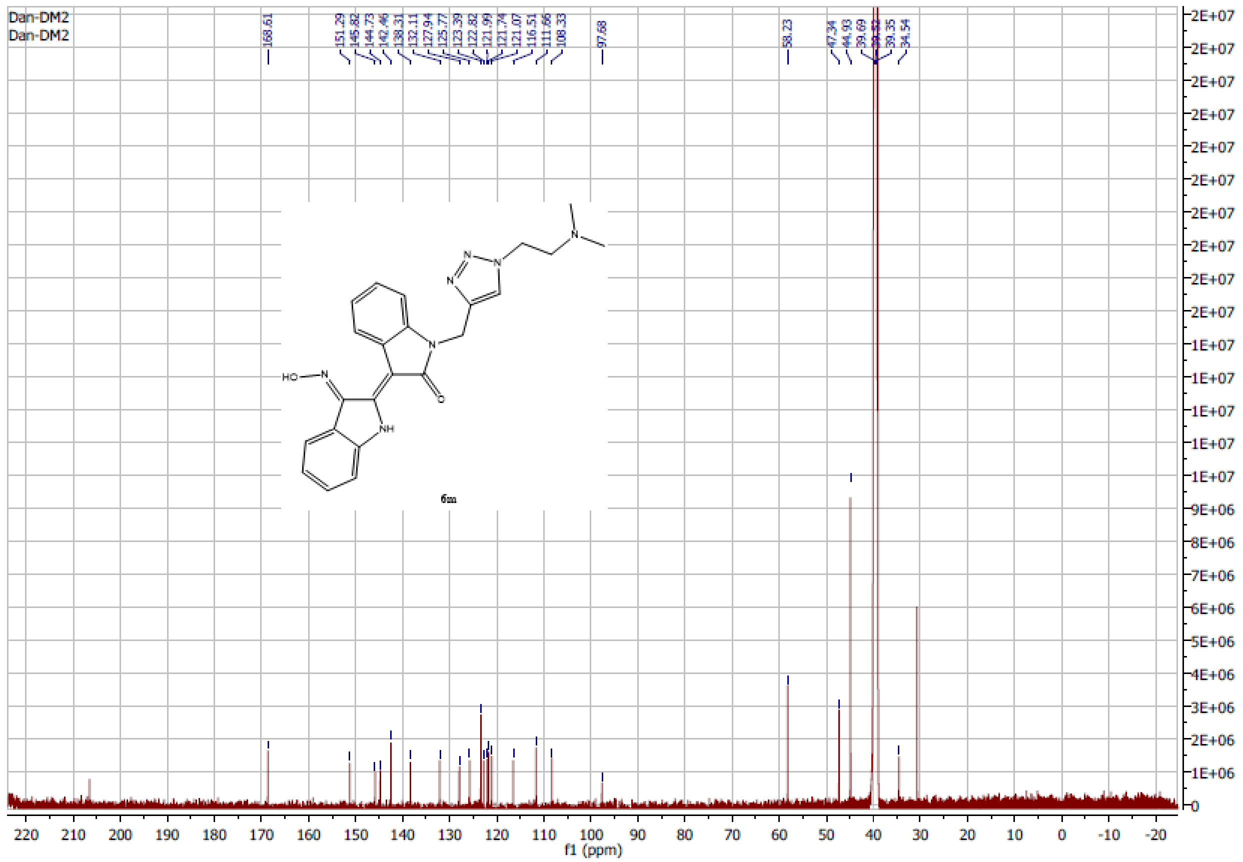

^{13}C-NMR spectrum of compound **6m**

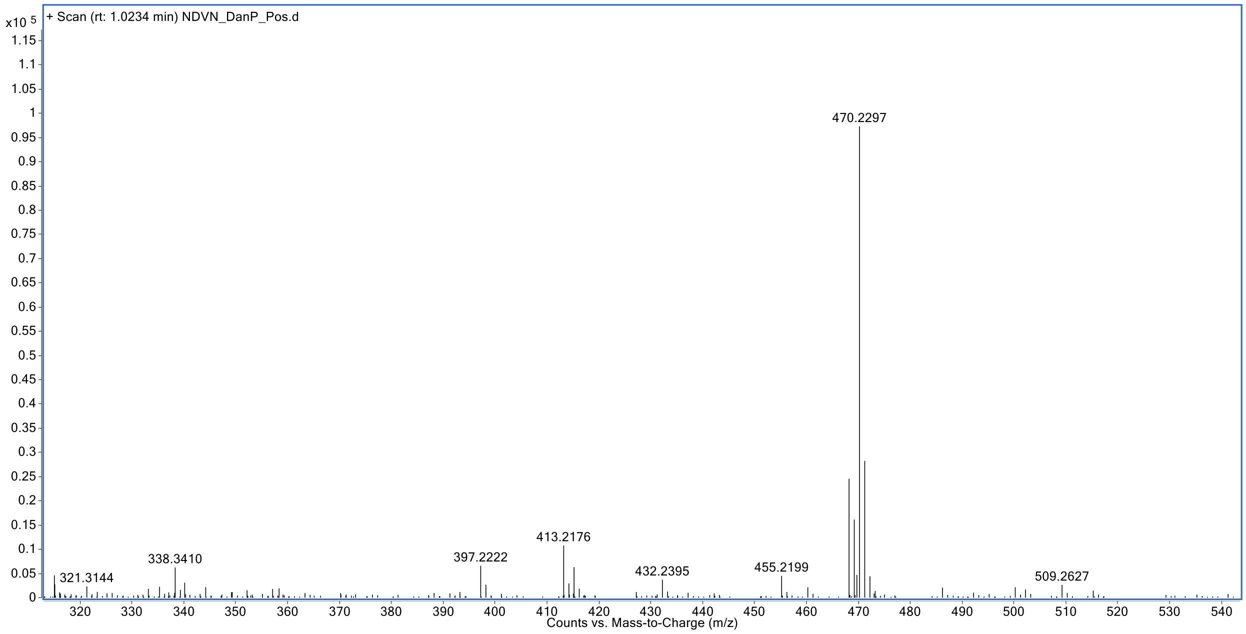

HR-MS(ESI) spectrum of compound **6n**

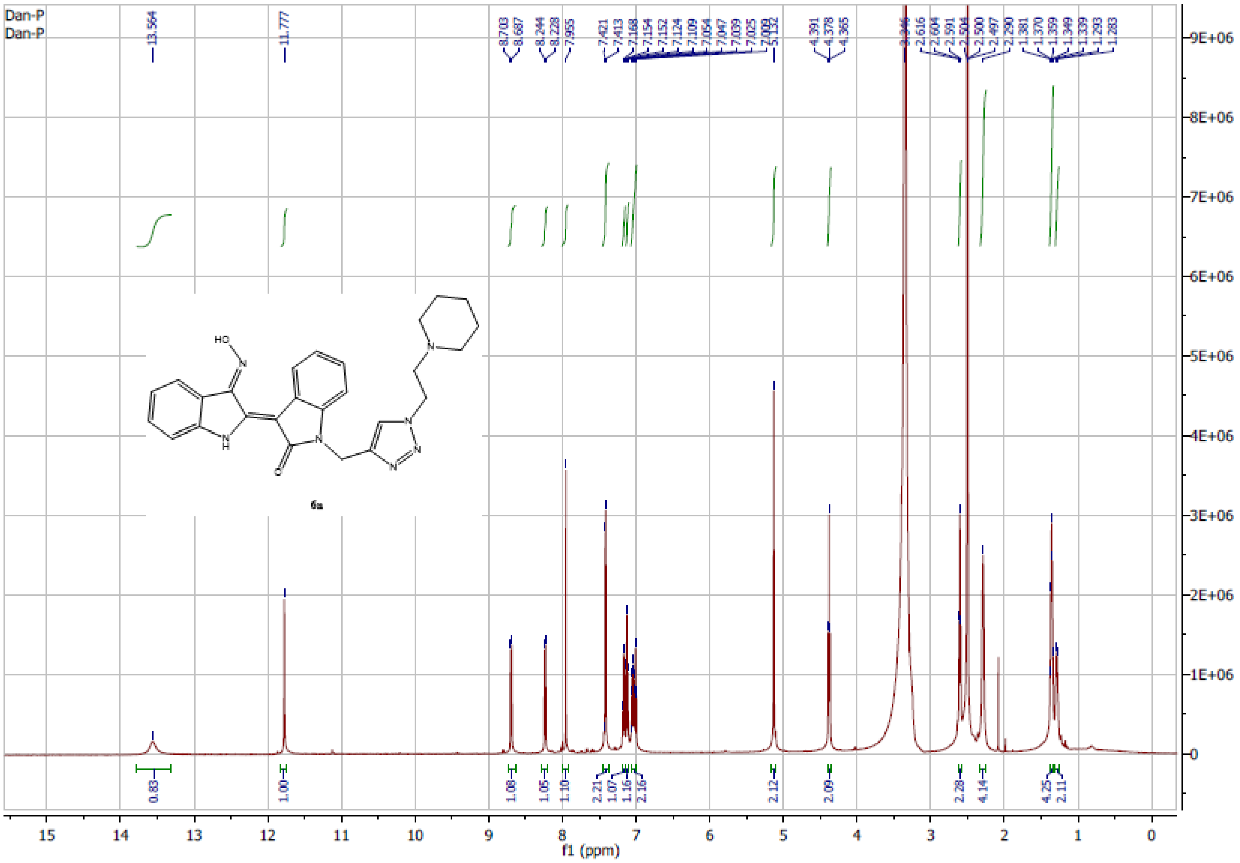

^{1}H-NMR spectrum of compound **6n**

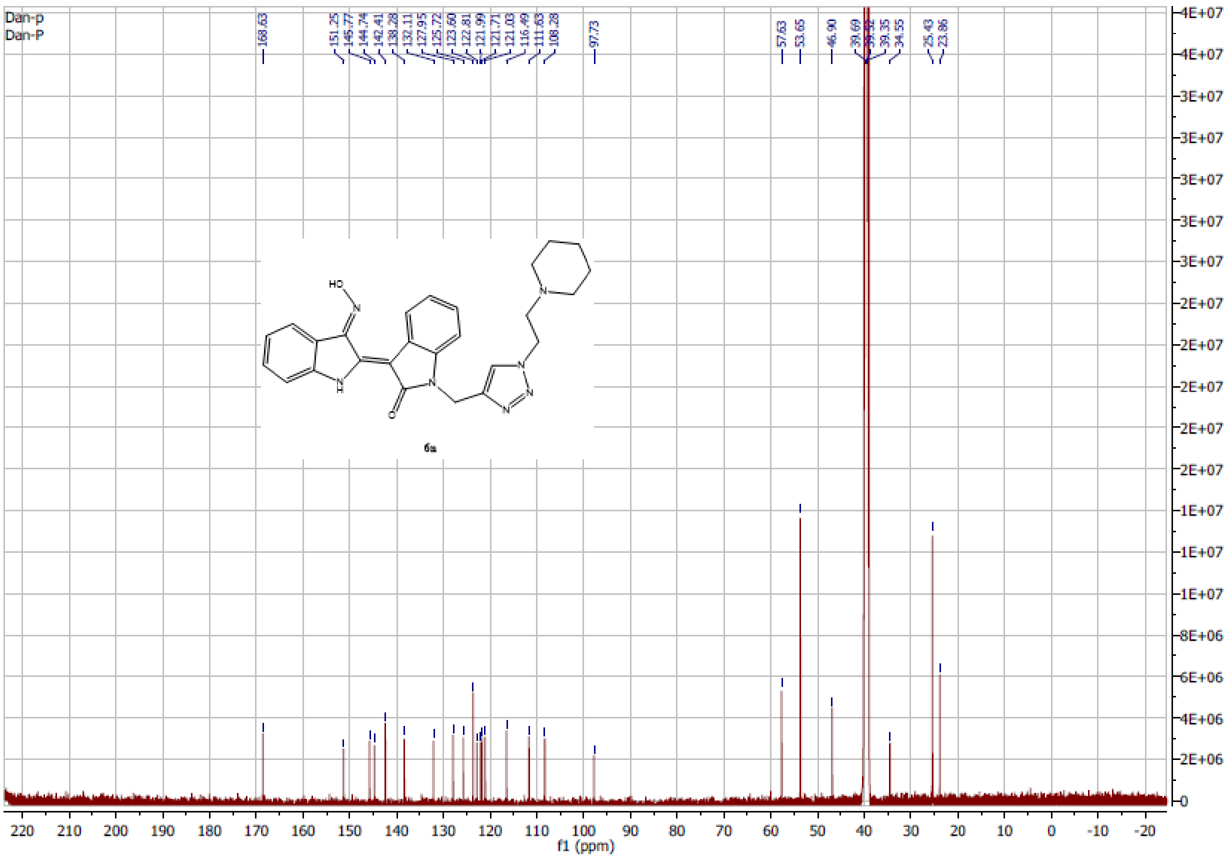

^{13}C-NMR spectrum of compound **6n**

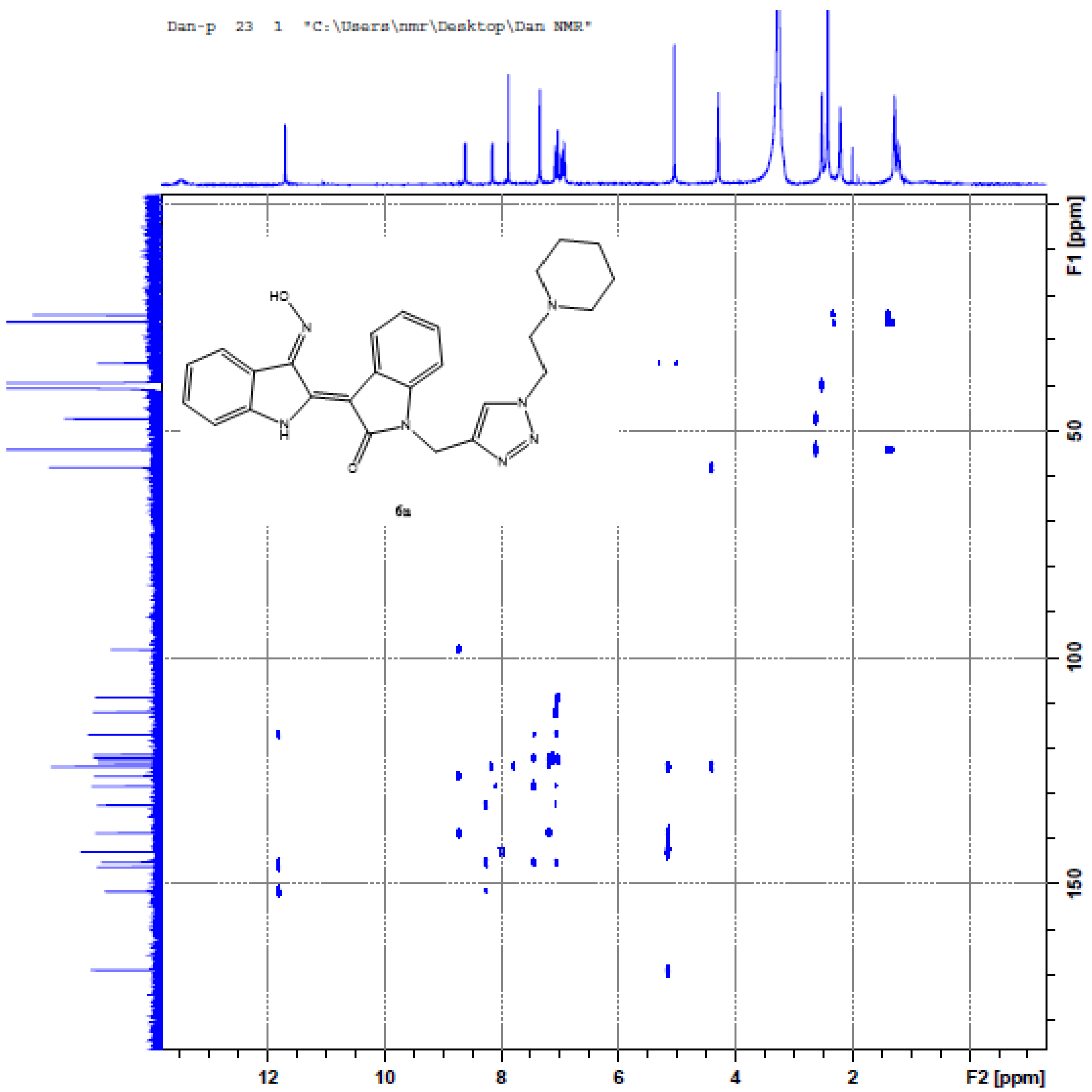

HMBC spectrum of compound **6n**

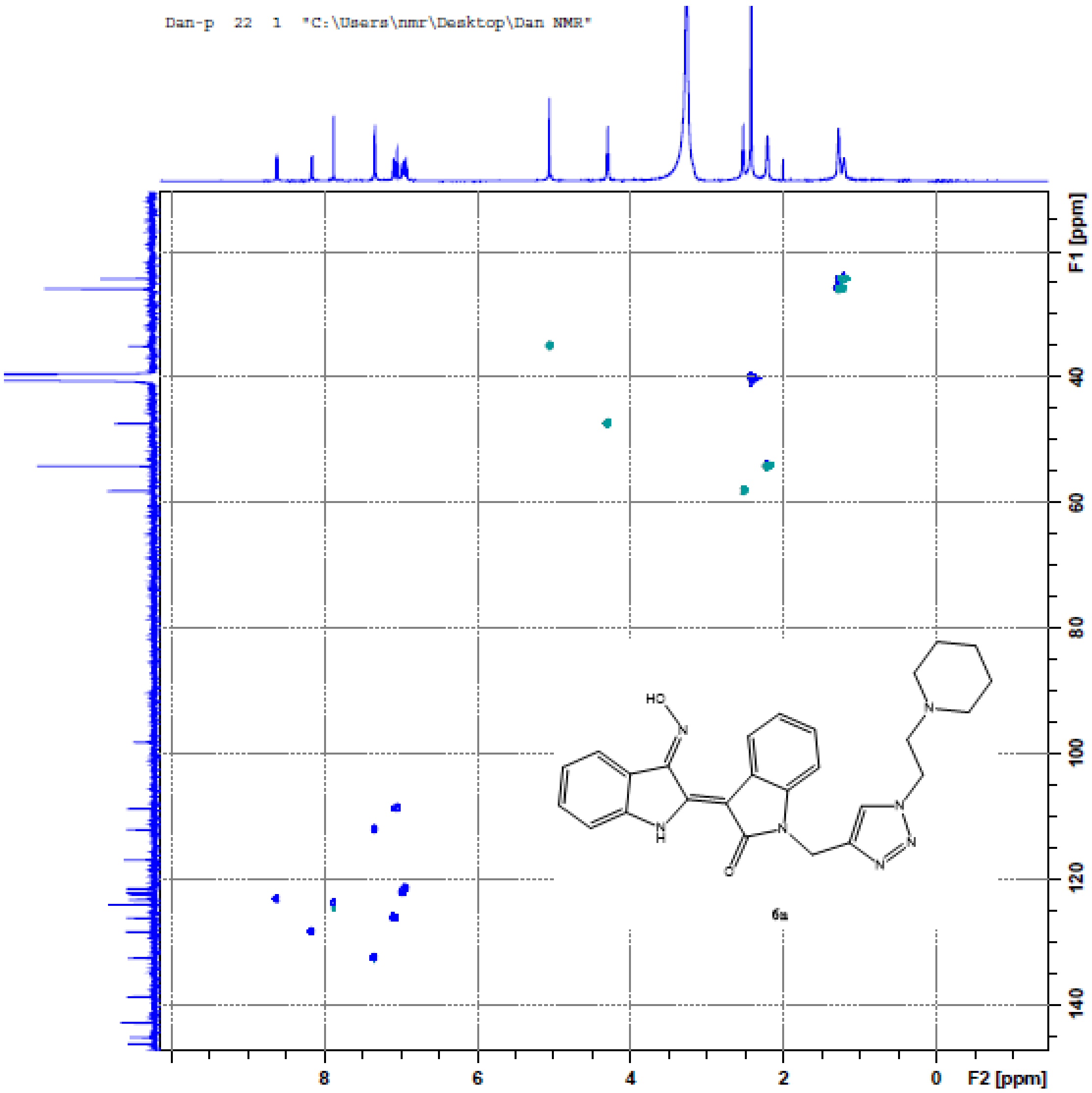

HSQC spectrum of compound **6n**

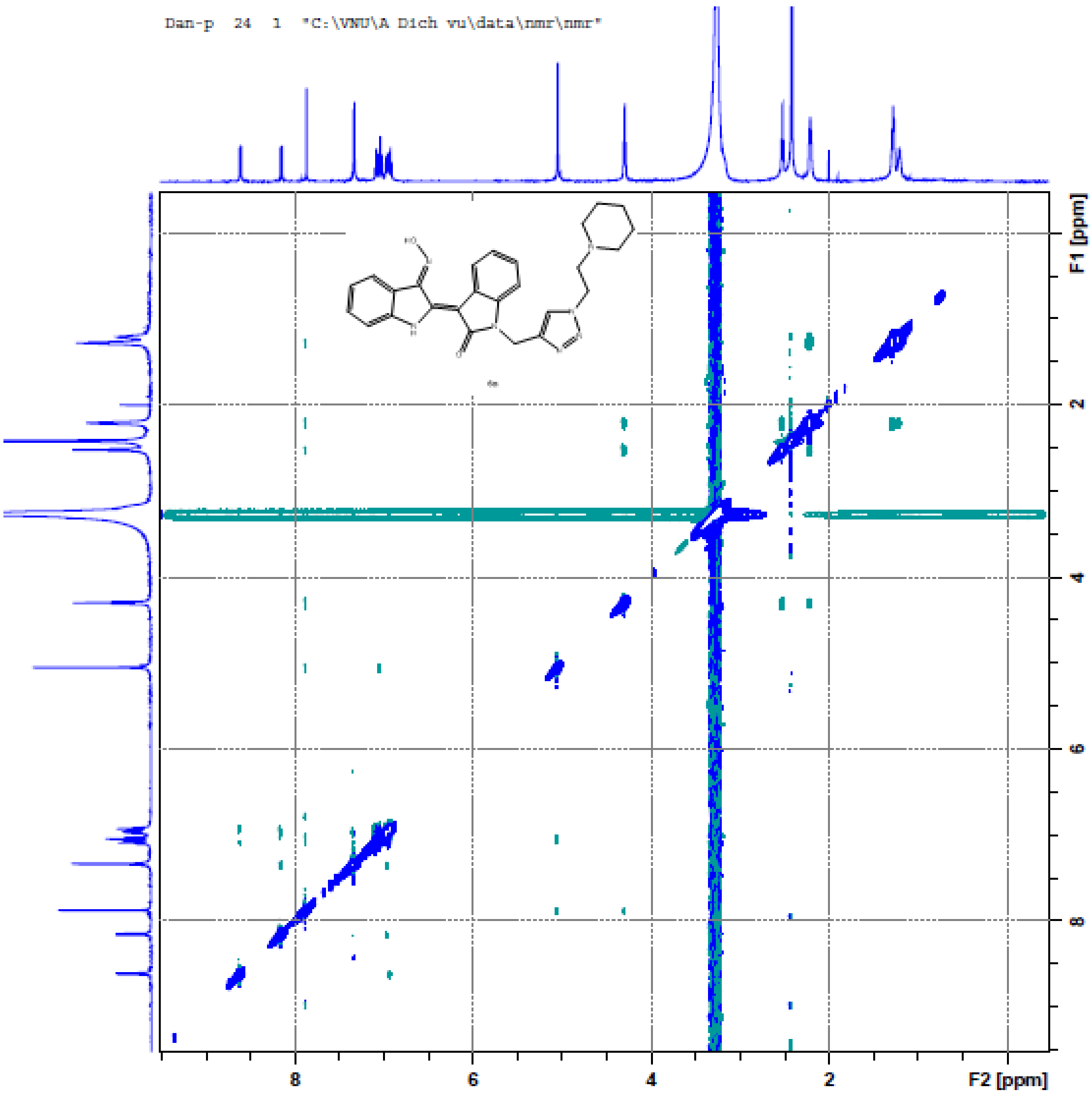

NOESY spectrum of compound **6n**

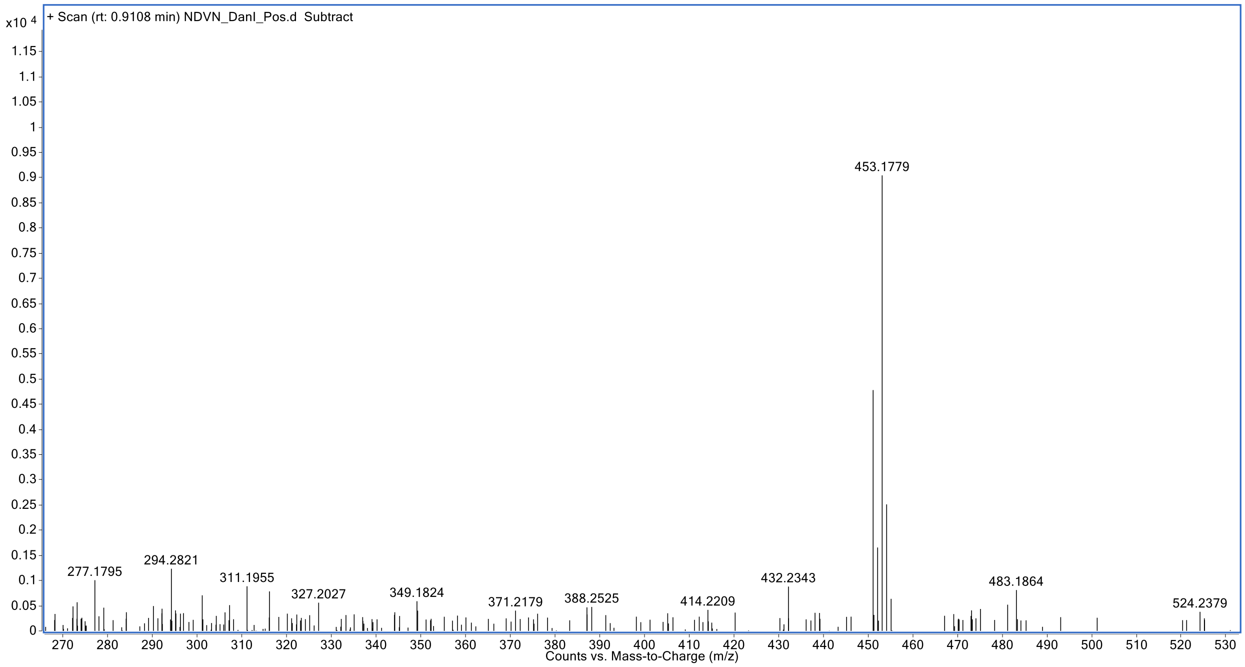

HR-MS(ESI) spectrum of compound **6o**

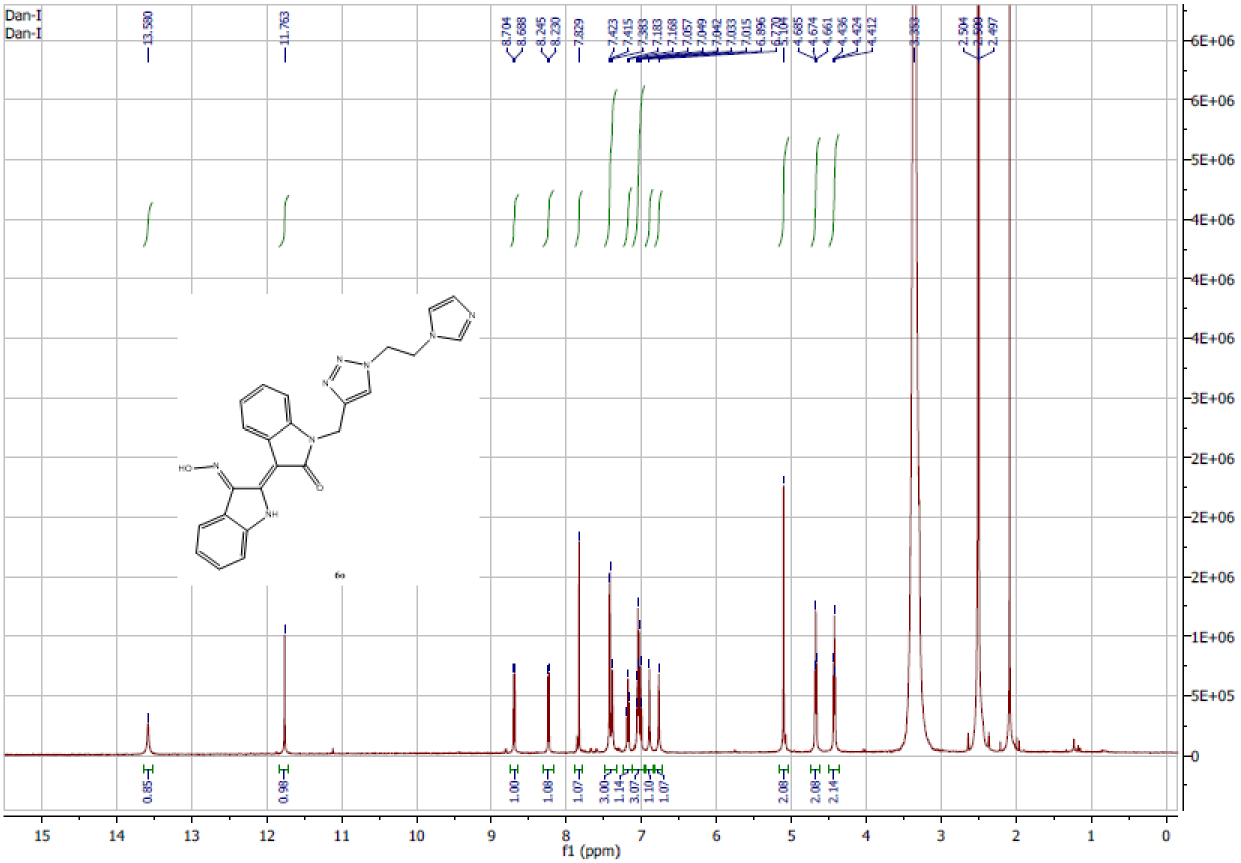

^{1}H-NMR spectrum of compound **6o**

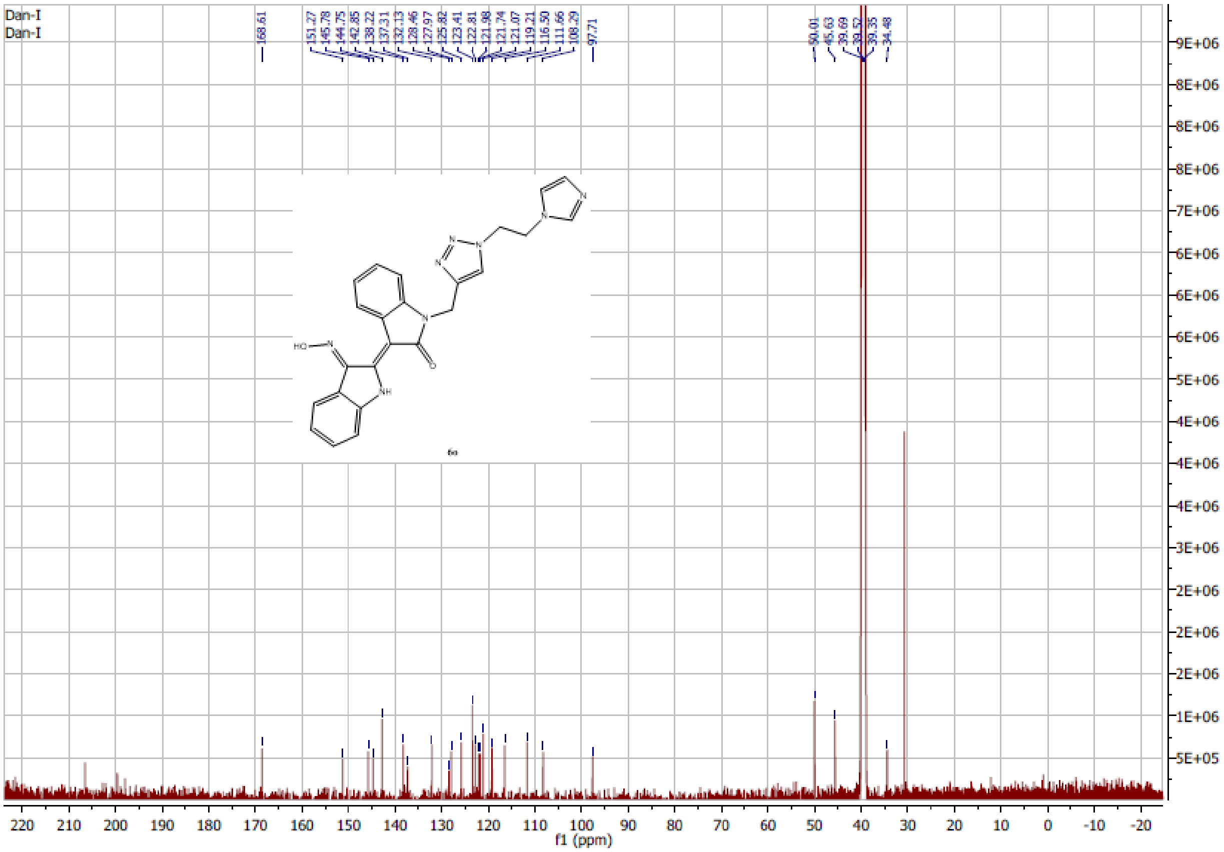

^{13}C-NMR spectrum of compound **6o**

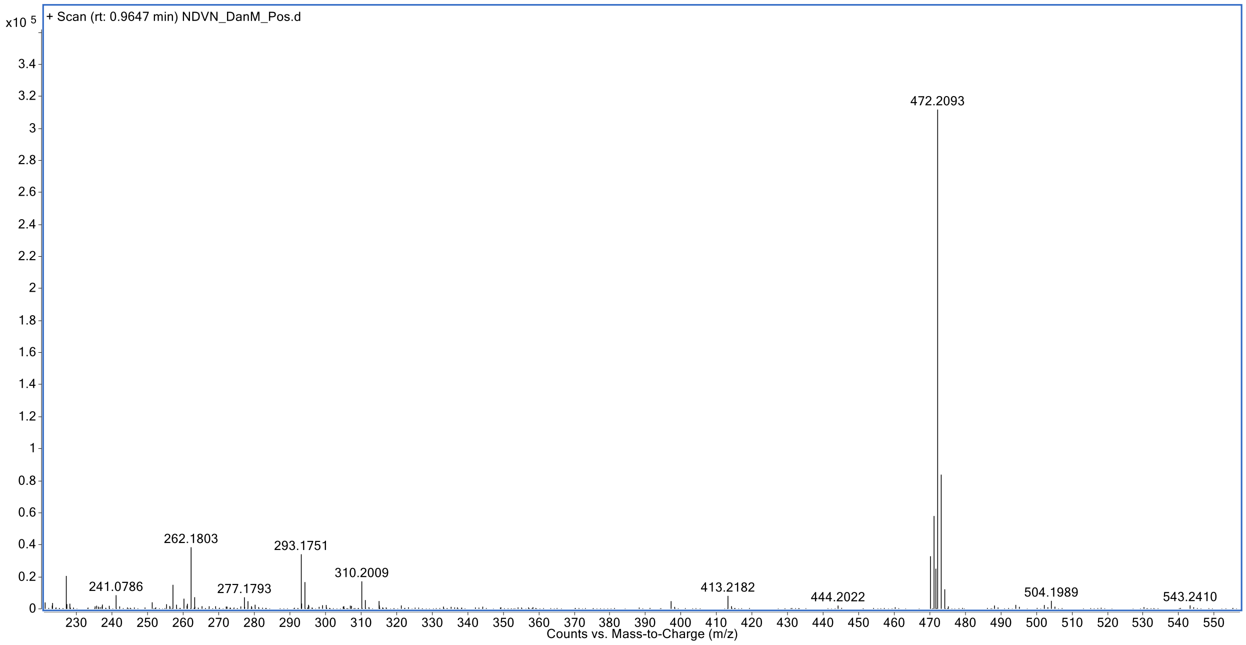

HR-MS(ESI) spectrum of compound **6p**

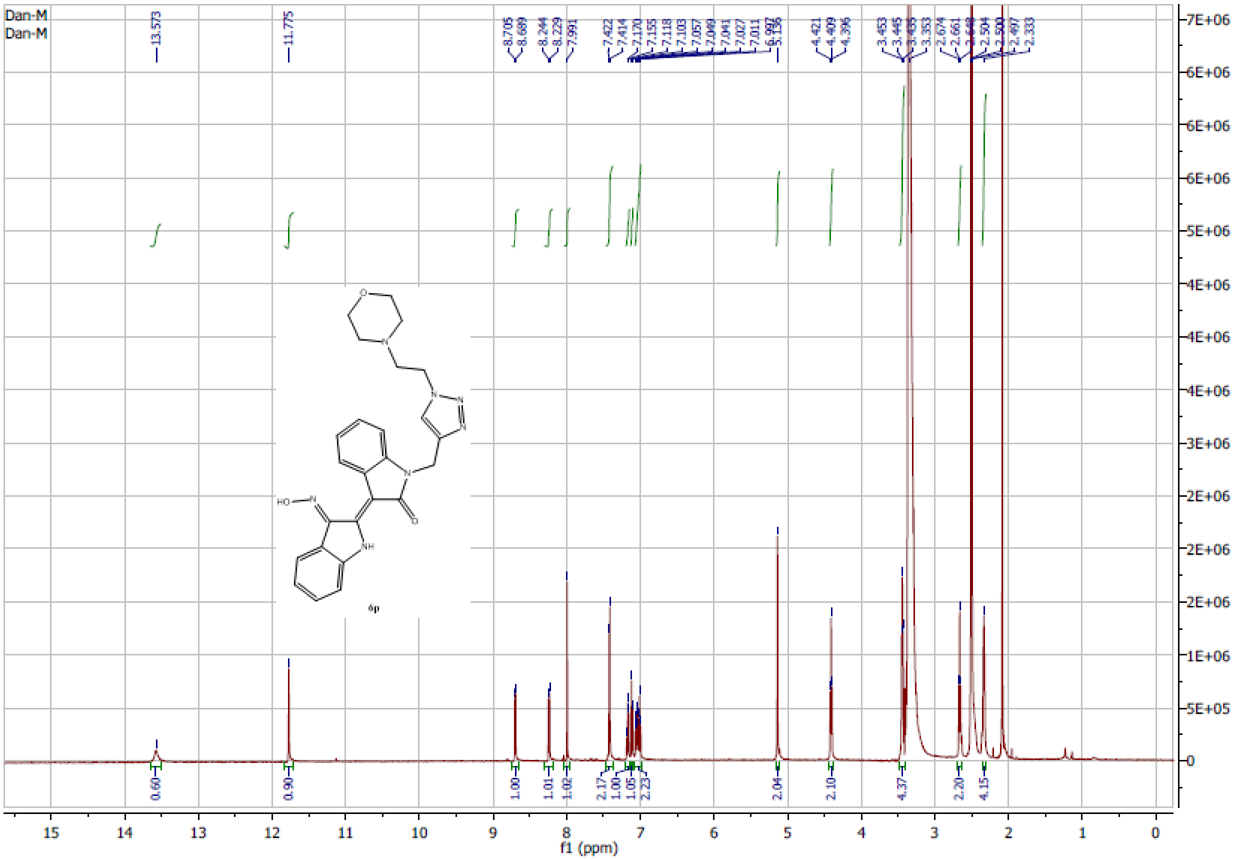

^{1}H-NMR spectrum of compound **6p**

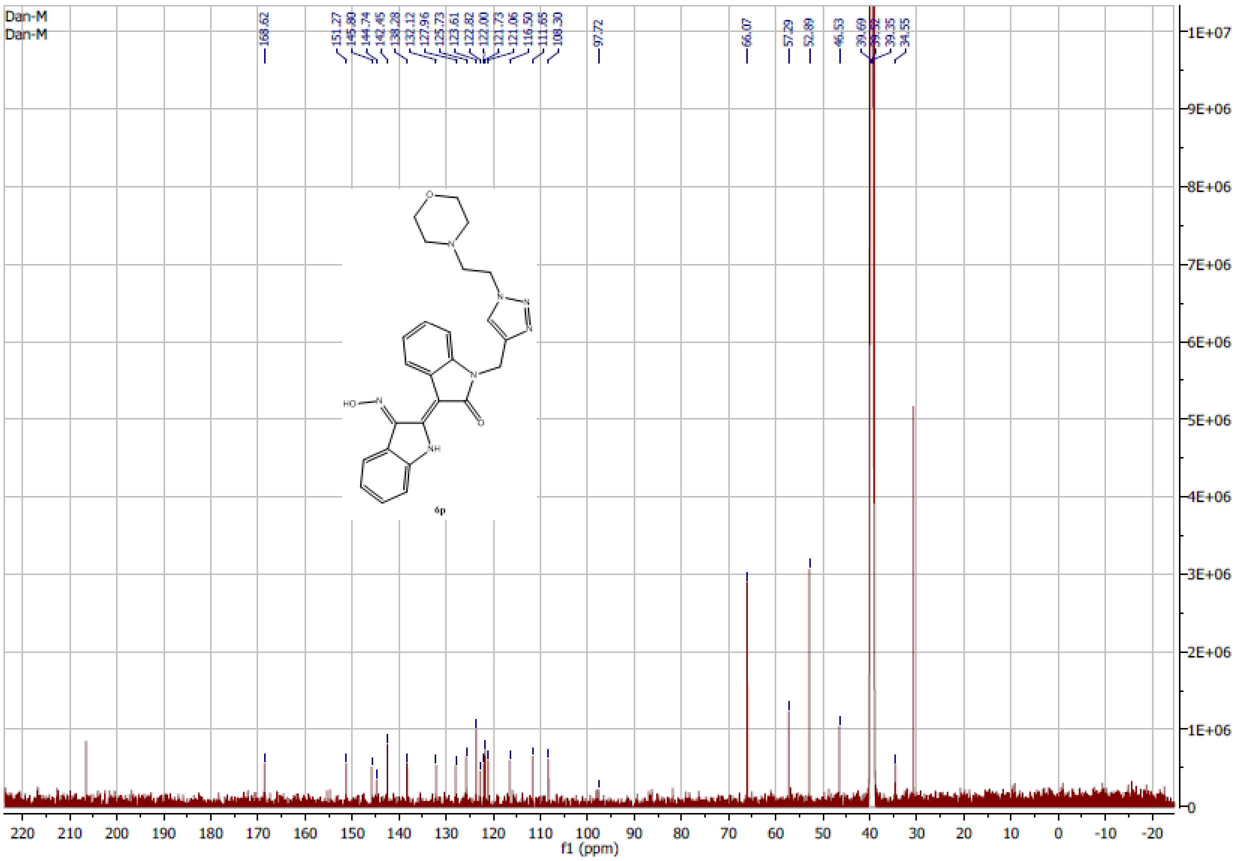

^{13}C-NMR spectrum of compound **6p**

References

1. Morris, G.M., Goodsell, D.S., Halliday, R.S., Huey, R., Hart, W.E., Belew, R.K. & Olson, A.J. Automated docking using a Lamarckian genetic algorithm and an empirical binding free energy function. *J. Comput. Chem* **19**, 1639-1662, https://doi.org/10.1002/(SICI)1096-987X(19981115)19:14<1639::AID-JCC10>3.0.CO;2-B (1998).
2. The PyMOL Molecular Graphics System. DeLano Scientific (San Carlos, CA, USA; 2002).
